# Adaptive robust position control scheme for an electromagnetic levitation system with experimental verification

**DOI:** 10.1371/journal.pone.0315457

**Published:** 2025-02-12

**Authors:** Ziwei Wu, Kuangang Fan, Ping Yi

**Affiliations:** 1 School of Electrical Engineering and Automation, Jiangxi University of Science and Technology, Ganzhou, China; 2 Magnetic Suspension Technology Key Laboratory of Jiangxi Province, Jiangxi University of Science and Technology, Ganzhou, China; 3 Ganjiang Innovation Academy, Chinese Academy of Sciences, Academy of Sciences, Ganzhou, China; University of Shanghai for Science and Technology, CHINA

## Abstract

Electromagnetic levitation technology has several advantages, such as no friction, safety, and reliability. Electromagnetic levitation control, as the core of electromagnetic levitation technology, has attracted people’s attention. The use of other traditional control algorithms frequently results in a decline in the system’s anti-disturbance and tracking performance due to the highly nonlinear, stochastic uncertainty, and time delay characteristics of electromagnetic levitation systems. This work takes the single point electromagnetic levitation ball system as the research object to address the above-mentioned issues. A control method combining an improved whale optimization algorithm with robust sliding mode control and adaptive linear active disturbance rejection (IWOA-SMC-ALADRC) is proposed to achieve stable control of a single point electromagnetic levitation ball. Firstly, a nonlinear model of the electromagnetic levitation ball system was established; Secondly, robust sliding mode control is combined with linear active disturbance rejection control, and an adaptive parameter tuning strategy is introduced for the PD module in LADRC; Meanwhile, an improved whale optimization algorithm was proposed to address the issue of excessive adjustable parameters in the controller; In addition, the stability and convergence of the control algorithm were proven using the Lyapunov equation; Finally, in order to verify the effectiveness of the control method, PID, LADRC, CS-LADRC, and I-LADRC were introduced for simulation analysis and experimental verification. The results indicate that IWOA-SMC-ALADRC has better anti-disturbance and tracking performance.

## 1 Introduction

Electromagnetic levitation, as an emerging technology, has advantages such as zero friction and low energy consumption [1, 2], it has been widely used in the aviation and transportation industries. But, achieving stable operation of electromagnetic levitation is currently a key issue that needs to be addressed [3, 4]. Due to the highly nonlinear, stochastic uncertainty, and time delay characteristics of electromagnetic levitation systems, on the one hand, it increases the difficulty of control, and from another perspective, the electromagnetic levitation system itself is easily affected by external and internal disturbances. So how to achieve effective control? Currently, it is a research hotspot for scholars [5–7].

Ouyang et al. [8] designed an automatic parameter tuning strategy for control and observation bandwidths for LADRC. This control method incorporates adaptive parameter tuning, enhancing the controller’ ability to resist interference and improve performance. However, the other adjustable parameters in the controller are debugged using experience, and the process is relatively cumbersome. Yu et al. [9] applied the pure LADRC control algorithm to the magnetic levitation system to address the problem of excessive overshoot in the application of traditional control algorithms in magnetic levitation systems. The step response analysis, anti-interference analysis, and tracking performance analysis demonstrated that pure LADRC has better dynamic performance and stability compared with the traditional PID algorithms and sliding mode control (SMC), but it does not solve the problem of parameter optimization. Ouyang et al. [10] designed an A-RBF algorithm to address the characteristics of excessive overshoot and slow start-up speed in the application of traditional control algorithms in magnetic levitation systems. By establishing a nonlinear model, high control accuracy was achieved. The study used ITAE and RMSE error indicators to quantify the simulation results, which more intuitively reflected the superiority of A-RBF. However, no comparison term was added in experimental verification. Wei et al. [11] combined the cuckoo search algorithm with LADRC to achieve a stable operation of the maglev system. This method optimizes parameters within a certain search range. However, the control effect has not been tested on the experimental platform. Qin et al. [12] designed an active disturbance rejection control (ADRC) method based on an improved levent differentiator (ILevent), which solves the problem of control performance degradation caused by unknown disturbances and input signal noise in magnetic levitation systems. Nevertheless, this method does not achieve adaptive parameter adjustment. Wei et al. [13] proposed the T-ADRC control method, which has better filtering performance but does not achieve parameter self-tuning. Zhong et al. [14] applied the full format model free adaptive control (FFDL-MFAC) method to a single degree of freedom magnetic levitation system to address the issue of nonlinearity and difficulty in establishing an accurate mathematical model. Although the method achieved good control accuracy, the simulation and experimental verification showed a limitation in the diversity of the input signal. Tian et al. [15] designed a three-step nonlinear controller to ensure that the system output has good dynamic quality when tracking the desired position trajectory changes. The controller consists of a class steady state control, variable reference feedforward control, and error feedback control, and it is derived in a fixed order in steps. The derivation of the three-step nonlinear controller is simple and clear. Meanwhile, the obtained control law structure is hierarchical and avoids the multiple differential problems caused by high-order nonlinearity, but it does not use error indicators to quantify the simulation results. Liu et al. [16] designed a track-based state feedback controller using the pole placement method, achieving a stable operation of the maglev system. Nonetheless, they only used MATLAB for simulation verification. Ma et al. [17] designed an adaptive grey prediction PID composite control strategy based on particle swarm optimization (PSO) for maglev systems, which has been proven to effectively suppress system lag and has good stability and robustness. However, the algorithm has not undergone theoretical stability analysis. Wang et al. [18] implemented high-precision and strong robustness motion control for magnetic levitation ball systems and proposed a proportional differential iterative learning algorithm with a forgetting factor. Although the control method has higher accuracy and robustness compared with PID control and SMC, it does not use non differentiable signals as input signals. Peng et al. [19] studied single point hybrid magnetic levitation balls. After improving the mathematical model of single point hybrid levitation balls, they studied the BP-PID control algorithm based on PSO. This algorithm has good control performance. Nevertheless, the parameter adjustment strategy is limited to finding optimal solutions within the set range, lacking the ability for autonomous parameter adjustment. Tang et al. [20] designed an improved sliding mode maglev control algorithm based on a linear extended state observer (LESO). The improved SMC based on a LESO effectively mitigates overshoot, has faster response speed, and significantly enhances the system anti-disturbance performance and robustness compared with traditional algorithms. This mechanism has theoretical guidance significance for overcoming the engineering implementation difficulties encountered by medium and low-speed maglev trains operating in various working conditions and complex environments. However, the verification of this algorithm has been limited to the numerical simulation stage. Yang et al. [21] proposed an improved LADRC method to address the issue of control performance degradation in single point electromagnetic levitation systems due to strong disturbance. The experimental results showed that the proposed improved LADRC exhibits good position tracking performance. However, sinusoidal signals were not used for analysis and verification in the experimental verification. Sun et al. [22] designed an SMC control with parameter self-tuning function. This control method demonstrates strong stability and anti-interference performance, but it lacks experimental analysis and exhibits a relatively monotonous approach. Hu et al. [23] designed an algorithm that combines SMC and ESO to ensure a stable operation of permanent magnet elect. This control method has good dynamic performance, but the input signal lacks diversity. Zhou et al. [24] implemented ADRC in remote sensing three-axis inertial stability control and achieved good control results, but the simulation project did not correspond to the physical verification project. They also designed a feedback linearization control method to mitigate the vibration phenomena prone to occur in maglev systems. The result indicated that this control method can enhance the stable suspension of the track and achieve certain control effects. However, the control scheme lacks theoretical analysis to substantiate its efficacy. Liu et al. [25] proposed a robust control method for PMM train speed tracking based on parameter self-tuning ADRC to address the challenge of achieving precise and stable speed tracking control during the intelligent driving of PMM trains under complex interference. This control method uses neural networks to adaptively tune the three gains of LESO. The experimental results showed that the BP-ADRC controller has significant advantages in improving the accuracy and robustness of PMM train speed tracking control compared with traditional train speed control methods, proving the effectiveness and feasibility of this method. However, no parameter adjustment plan has been proposed for the parameters in the PD module. Wang et al. [26] proposed an improved ADRC to address the large time delay problem in speed tracking control of maglev trains. The simulation results showed that, under the same road conditions, this control method can accurately track the target speed curve on different road sections compared with the maglev train speed tracking system based on ADRC and 2DOF-PID controllers. This control method also has good reference value for academic research and engineering applications of other motion control problems. However, this control method lacks data analysis, relying solely on simulation results. Wang et al. [27] designed a full state feedback optimal controller to effectively suppress the coupled vibration of maglev vehicle rail and simplified the vehicle rail coupled vibration system into a single iron elastic track model. The verification results indicated that the control algorithm suppresses the coupled vibration of maglev vehicle tracks, while maintaining stable suspension, reducing the system’s excessive dependence on track beam characteristics for stability. However, no convergence analysis was conducted on the controller. Yang et al. [28] designed a control method that combines improved particle swarm optimization algorithm and ADRC to address the issue of train speed tracking control. The simulation results show that compared with traditional algorithms, the ADRC algorithm exhibits advantages such as strong disturbance rejection ability, high tracking accuracy, and fast response speed. This method has certain academic reference significance and practical application value for other speed tracking and even most motion control, but no error analysis has been made. Chen et al. [29] provided a detailed introduction to the structure and working principle of the maglev system and established the physical and corresponding mathematical models of the system. The stability of the system and the selection of control schemes were analyzed using the physical and mathematical models of the system. Finally, a system simulation model was established in the MATLAB/Simulink environment to study the trajectory tracking of the control system. A backstepping control method based on two different V functions was proposed to design a nonlinear controller and achieve fast, accurate, and stable trajectory tracking control of the maglev system. The simulation experiments were conducted to analyze and compare the tracking performance of the two control methods. Reasonable suggestions were provided for the overall control scheme design. The experiments showed that both controllers can stably achieve control objectives, but no data analysis was conducted. Shen et al. [30] designed a flexible magnet based on full state feedback considering the vibration of track plates suspension control algorithm, combined with traditional rigid control algorithms that do not consider track plate vibration method for comparison. analysed the impact of track plate stiffness on the stability of magnetic levitation systems explored the magnetic levitation system under different control algorithms in the presence of track foundation excitation unified response. The results indicate that a rigid controller without considering the vibration of the track plate has an impact on the track the requirement for plate stiffness is high, and it is sensitive to track foundation excitation, which can easily cause vibration oscillation and instability; The flexible control algorithm considering the vibration of the track plate requires the stiffness of the track plate seeking a lower level, stability can still meet the requirements under the influence of basic incentives. However, this work needs to be further verified through actual track systems.

In this study, we use a control method that combines robust sliding mode control and LADRC to achieve stable operation of the electromagnetic levitation ball system. We designed an adaptive parameter adjustment strategy for the linear active disturbance rejection PD module, which adjusts parameters in real time based on the error of system. Meanwhile, use the Improved Whale Optimization Algorithm (IWOA) to confirm the remaining adjustable parameters in the controller. This control method solves the problem of reduced disturbance resistance and tracking performance of traditional control algorithms in the application of electromagnetic levitation ball systems, further promoting the application of LADRC in the field of electromagnetic levitation. The rest of this paper is organized as follows. Section 2 establishes the mathematical model of the electromagnetic levitation ball system. Section 3 presents the designed controller that combines sliding mode control with ALADRC. Section 4 introduces an IWOA. Section 5 provides the Lyapunov equation to analyze the theoretical stability and convergence of the designed algorithm. Section 6 conducts a simulation analysis on the control method. Section 7 performs an experimental verification of the proposed algorithm on the electromagnetic levitation experimental platform. Section 8 provides our findings.

## 2 Electromagnetic levitation ball model

### 2.1 Introduction to the system operating principles

**Fig 1:**
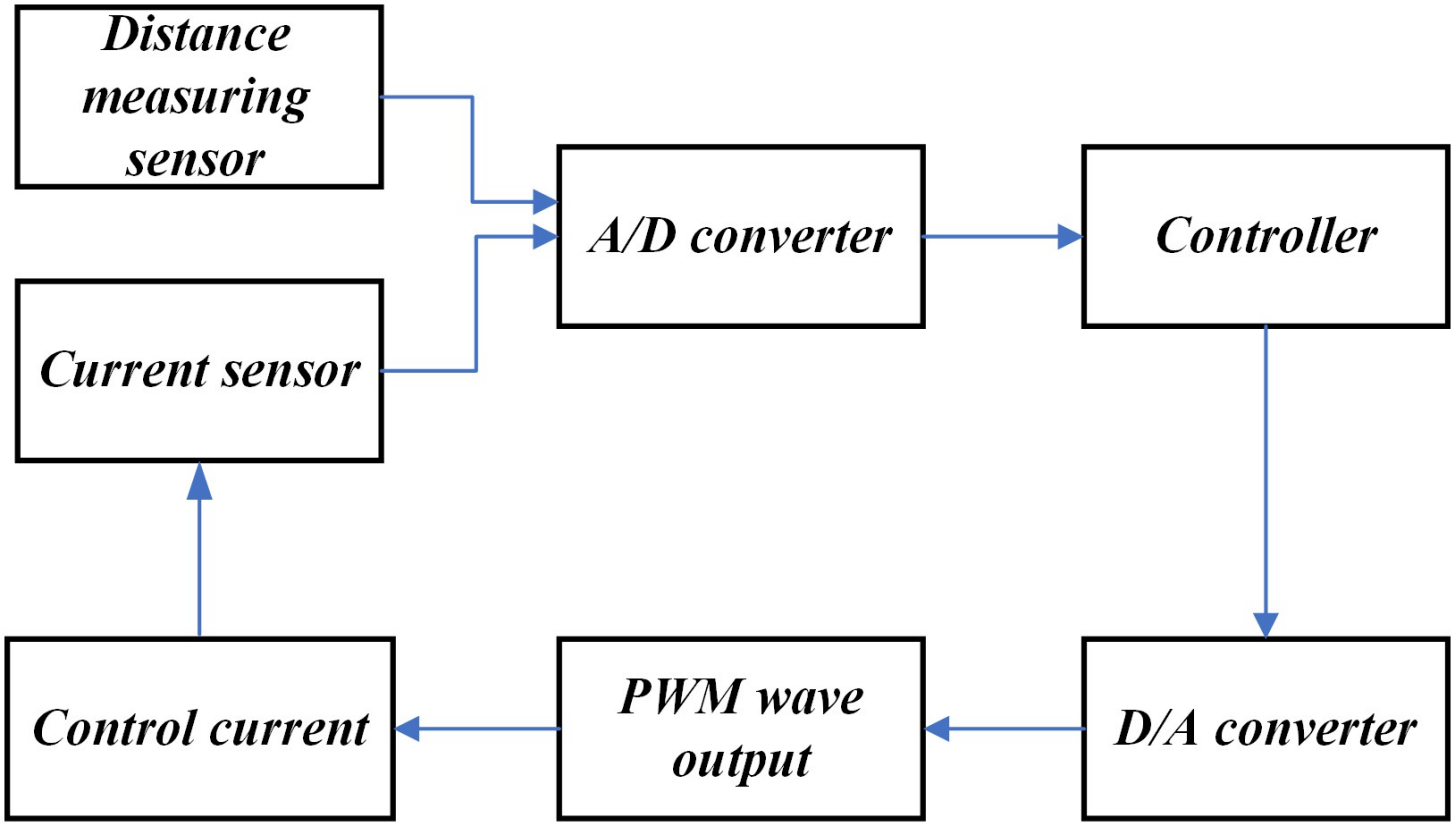
Block diagram of the operating principle of electromagnetic levitation system.

Before establishing the model of the electromagnetic levitation ball system, the operating principle of the system must be explained. First, various sensors detect and obtain real-time position and current signals of the magnetic levitation ball. After the signals are processed by signal processing and driving circuits, they are converted into the corresponding voltage signals through A/D converters. Second, the obtained voltage signal is fed back and used as an input for the suspension controller. The suspension controller utilizes a certain suspension control algorithm for calculation and a D/A converter to obtain appropriate output control instructions. Finally, the signal is converted into a PWM signal with a certain duty cycle, and the PWM signal is transmitted to the suspension chopper in the form of PWM waves. The suspension chopper then converts the signal into the required current value in the electromagnetic device, thereby reducing or increasing the required electromagnetic force at the suspension point, causing it to be subjected to downward or upward force. Consequently, the magnetic levitation ball moves either downward or upward, ensuring that the force between the two is equal, and the system operates. The operating principal block diagram is shown in Fig 1.

The electromagnetic levitation ball system belongs to a closed-loop control system. Accordingly, stable levitation is achieved by taking the difference in levitation height between the electromagnet and the magnetic levitation ball as an input. Real-time feedback is continuously provided to the control center. Finally, output an appropriate current value to control the controlled object. Due to the inherent characteristics of the electromagnetic levitation ball system, its balance is extremely unstable, which is determined by the nonlinearity of the electromagnetic force generated by the electromagnetic field itself. The magnitude of electromagnetic force generated by electromagnetic fields is inversely proportional to the square of the suspended air gap between suspended objects. When the current in the coil is constant, the larger the suspended air gap value, the smaller the electromagnetic force generated by the electromagnetic field; The smaller the suspension air gap value, the greater the electromagnetic force generated by the electromagnetic field. If the suspension air gap value increases, then the coil current in the electromagnetic field also increases, thereby reducing the suspension air gap value and further increasing it; If the suspended air gap value decreases, the coil current in the electromagnetic field also decreases, thereby reducing the suspended air gap value and further decreasing it. By continuously adjusting feedback to change the current in the coil, the change in coil current can indirectly affect the electromagnetic force generated by the electromagnet, thereby keeping the electromagnetic levitation ball system in a dynamic and stable equilibrium at all times. Therefore, the designed suspension control algorithm must also improve the robustness and stability of the controlled object, and the algorithm must have the advantage of short adjustment time.

### 2.2 Establishing mathematical models

Considering the high non-linearity of the electromagnetic suspension system, we need to make the following assumptions before establishing the model:

(1) Assuming the absence of magnetic resistance in the iron core;
(2) Assuming a uniform distribution of magnetic field in the air gap;
(3) Assuming that the magnetic field in the iron core is uniformly distributed.

**Fig 2:**
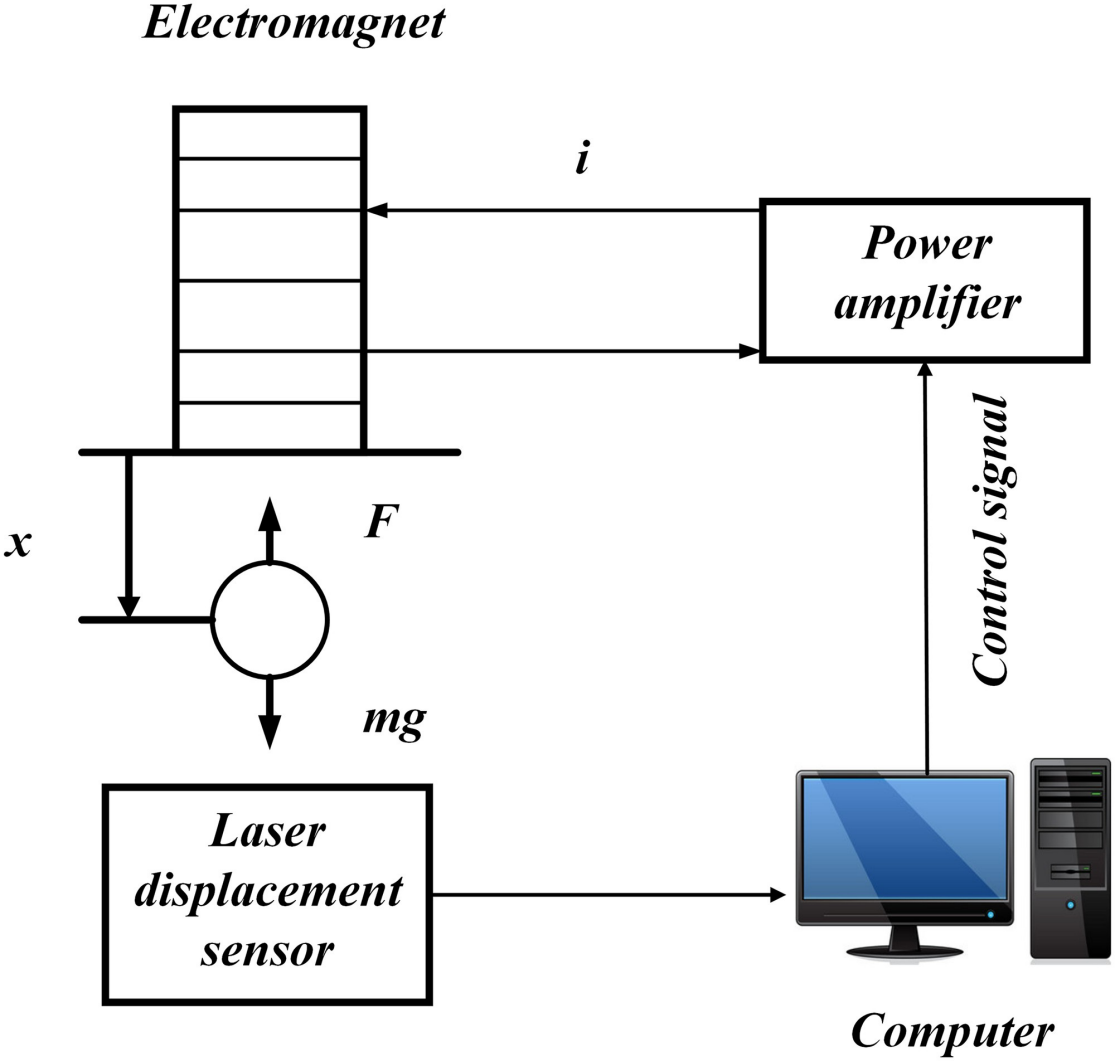
Electromagnetic levitation system.

We have simplified the diagram to facilitate the establishment of the system model. The simplified diagram is shown in Fig 2:

After simplification, the electromagnetic levitation ball system consists of laser displacement sensors, power amplifiers, computers, and electromagnets. First, the laser displacement sensor emits laser to obtain real-time position information of the magnetic levitation ball, which is converted into the corresponding signals through an A/D converter and continuously fed back to the computer. Secondly, the computer control end obtains output instructions through corresponding control algorithms, which are then transmitted to the power amplifier; Finally, it generates corresponding electromagnetic force by controlling the current value in the coil.

The nonlinear dynamic equation of the magnetic levitation ball system can be obtained from Fig 2 as follows:

$$\begin{array}{c} \left\{ \begin{array}{c} m\frac{d^{2}x\left(t\right)}{dt^{2}}=F\left(i,x\right)+mg,\\ F\left(i,x\right)=\frac{\mu_{0}AN^{2}}{4}{\left(\frac{i}{x}\right)}^{2},\\ mg+F(i_{0},x_{0})=0,\\ U(t)=Ri(t)+L_{1}\frac{di(t)}{dt} \end{array}\right. \end{array}$$ (1)

In Eq (1), *U*(*t*) is the instantaneous voltage applied to both ends of the electromagnetic coil; *x* is the distance from the surface of the steel ball to the surface of the electromagnet, which is the suspended air gap; *x*_0_ is the distance indicated by the surface of the steel ball to the electromagnet when the steel ball is at the equilibrium point; *m* is the weight of the steel ball; *i* is the amount of current flowing through the electromagnetic coil; *i*_0_ is the current value that flows through the electromagnetic coil when the steel ball is at the equilibrium point; *F*(*i*, *x*) is the instantaneous electromagnetic force; *F*(*i*_0_, *x*_0_) is the electromagnetic force experienced by the steel ball at the critical position; *K*_*a*_ is the gain of the power amplifier; *N* is the number of turns of the electromagnetic coil; *A* is the magnetic conductivity cross-sectional area; and *μ*_0_ is the vacuum magnetic permeability.

According to Eq (1), the electromagnetic suction force *F*(*i*, *x*) is related to the current *i* and the position *x* of the steel ball, and their relationship is nonlinear. The electromagnetic force is Taylor expanded at the equilibrium point (*i*_0_, *x*_0_). After the higher-order term is removed, the electromagnetic force can be expressed as Eq (2)

$$\begin{array}{c} F(i,x)=F(i_{0},x_{0})+\frac{\mu_{0}N^{2}i_{0}A}{2x_{0}^{2}}(i-i_{0})-\frac{\mu_{0}N^{2}i_{0}^{2}A}{2x_{0}^{3}}(x-x_{0}) \end{array}$$ (2)

Substituting Eq (2) into Eq (1) yields:

$$\begin{array}{c} m\frac{d^{2}x(t)}{dt^{2}}=\frac{\mu_{0}N^{2}i_{0}A}{2x_{0}^{2}}i-\frac{\mu_{0}N^{2}i_{0}^{2}A}{2x_{0}^{3}}x \end{array}$$ (3)

Applying Laplace transform to Eq (3) yields:

$$\begin{array}{c} s^{2}X(s)=\frac{1}{m}\frac{\mu_{0}N^{2}i_{0}A}{2x_{0}^{2}}I(s)-\frac{1}{m}\frac{\mu_{0}N^{2}i_{0}^{2}A}{2x_{0}^{3}}X(s) \end{array}$$ (4)

The following expression exists due to the power amplifier:

$$\begin{array}{c} K_{a}=\frac{U(s)}{I(s)} \end{array}$$ (5)

Substituting Eq (5) into Eq (4) yields:

$$\begin{array}{c} s^{2}X(s)=\frac{1}{m}\frac{\mu_{0}N^{2}i_{0}A}{2x_{0}^{2}}U(s)-\frac{1}{m}\frac{\mu_{0}N^{2}i_{0}^{2}A}{2x_{0}^{3}}X(s) \end{array}$$ (6)

According to Eq (6), the expression of the controlled object in simulation is presented as follows:

$$\begin{array}{c} G\left(s\right)=\frac{\frac{1}{m}\frac{\mu_{0}N^{2}i_{0}A}{2x_{0}^{2}K_{a}}}{s^{2}+\frac{1}{m}\frac{\mu_{0}N^{2}i_{0}^{2}A}{2x_{0}^{3}}} \end{array}$$ (7)

Let *x* = *x*_1_, ${\dot{x}}_{1}=x_{2}$, and *y* = *x*_1_. The electromagnetic levitation system can be represented as follows:

$$\begin{array}{c} \left\{ \begin{array}{c} \dot{x}=Ax+Bu+Cd\left(t\right)\\ y=Dx \end{array}\right. \end{array}$$ (8)

where

$$\begin{array}{c} A=[\begin{array}{cc} 0 & 1\\ 0 & 0 \end{array}],B=[\begin{array}{c} 0\\ b_{0} \end{array}],C=[\begin{array}{c} 0\\ 1 \end{array}],D=[\begin{array}{c} 1\\ 0 \end{array}] \end{array}$$

where *x*_1_ is the suspended air gap of the steel ball, *x*_2_ is the shaking rate of the steel ball, and *d*(*t*) is the total disturbance of the system.

The significance and parameter values of the specific parameters of the magnetic levitation system are shown in Table 1.

**Table 1:** Specific parameter values of the electromagnetic levitation ball system.

Evaluation Indicators	Values
**The mass of steel ball**	*m* = 0.094(*kg*)
**Coil tums**	*N* = 2450
**Current in the coil at balance**	*i*_0_ = 0.5752(*A*)
**The suspension gap when the steel ball is balanced**	*X*_0_ = 0.01(*m*)
**Magnetic pole area**	*A* = *π* × 10^−4^ (*m*^2^)
**Permeability of vacuum**	*μ*_0_ = 4*π* × 10^−7^ (*H*/*m*
**Ball radius**	*r* = 0.00125 (*m*)
**Coil resistance**	*R* = 13.8(Ω)

## 3 Controller design

**Fig 3:**
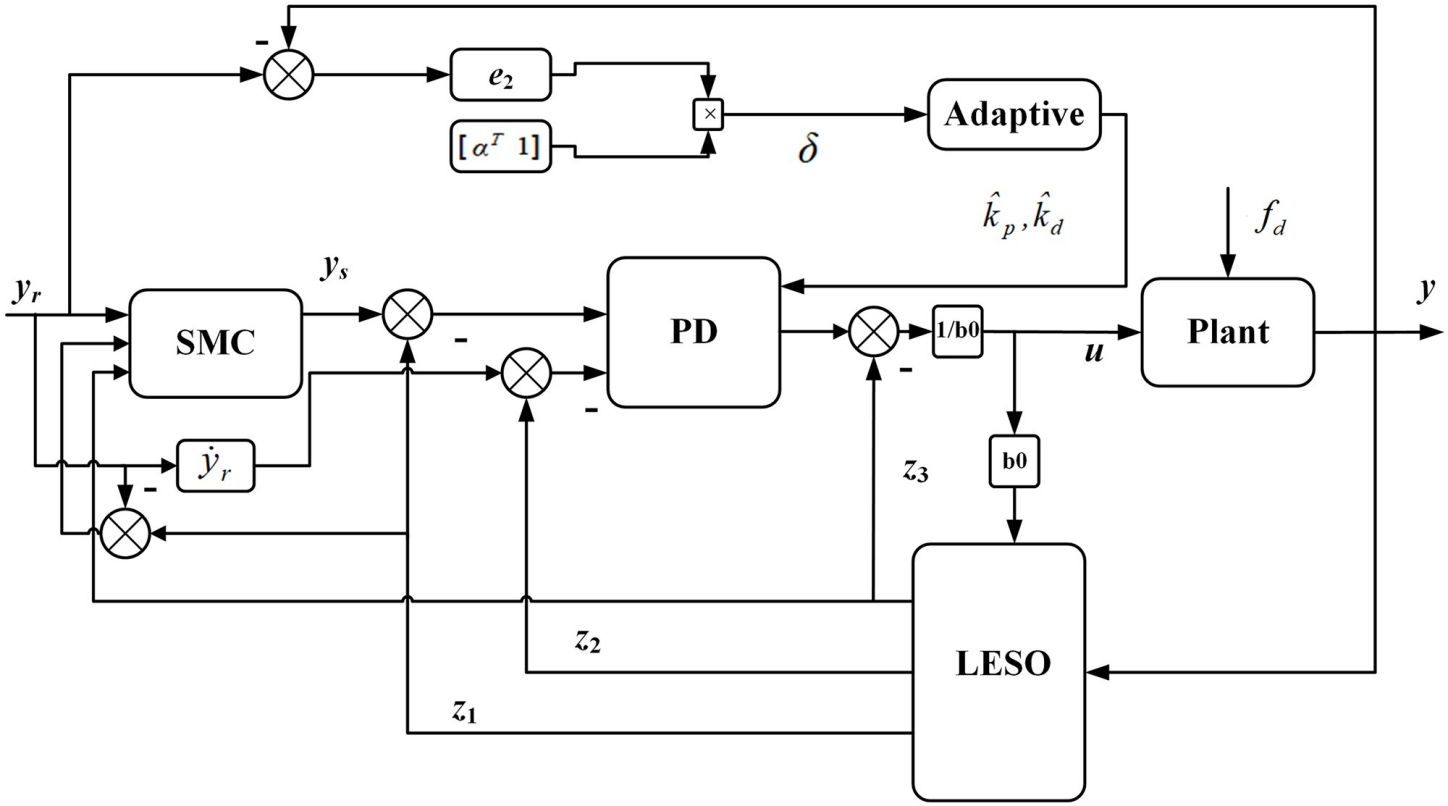
Controller structure.

An SMC-ALADRC control method is designed to address the issues of anti-disturbance performance and poor tracking performance in the application of traditional control methods in electromagnetic levitation ball systems. This solution combines the two major technological advantages of SMC and LADRC. SMC improves the robustness and anti-disturbance performance and overcomes the problem of low control accuracy caused by the bandwidth limitation of LADRC. The LESO can estimate the external disturbances and unmodeled internal dynamics of the electromagnetic levitation ball system as the total disturbance of the system in real time and compensate for the total disturbance estimated by the LESO through the PD module. An adaptive parameter adjustment strategy is introduced in the PD module of LADRC to simplify parameter tuning. This strategy can adaptively adjust parameters *k*_*p*_ and *k*_*d*_ according to the system’s operating conditions. The controller structure is shown in Fig 3.

### 3.1 Robust SMC design

The robust sliding surface can be expressed as Eq (9)

$$\begin{array}{c} s=w_{1}e_{1}+{\dot{e}}_{1} \end{array}$$ (9)

where *w*_1_ is a positive constant, *e*_1_ = *z*_1_ − *y*_*r*_ is the error between the evaluation signal output by the LESO and the input signal of the system, and *y*_*r*_ is the input signal of the system. We can obtain the following expression by differentiating the sliding surface:

$$\begin{array}{c} \dot{s}=w_{1}{\dot{e}}_{1}+{\ddot{e}}_{1}=w_{1}{\dot{e}}_{1}+z_{3}+b_{0}u-{\ddot{y}}_{r} \end{array}$$ (10)

The control law of sliding mode can be expressed as follows:

$$\begin{array}{c} y_{s}=y_{seq}+y_{ssw} \end{array}$$ (11)

where *y*_*seq*_ represents the equivalent part, *y*_*ssw*_ represents the switching part, and they are represented as follows:

$$\begin{array}{c} y_{seq}={\ddot{y}}_{r}-w_{1}{\dot{e}}_{1}-z_{3} \end{array}$$ (12)

$$\begin{array}{c} y_{ssw}=-w_{2}s \end{array}$$ (13)

Therefore, the sliding mode control law *y*_*s*_ can be expressed as:

$$\begin{array}{c} y_{s}={\dot{y}}_{r}-w_{1}{\dot{e}}_{1}-z_{3}-w_{2}s \end{array}$$ (14)

where *w*_2_ is a positive parameter.

### 3.2 Design of LADRC

The control signal can be represented as follows:

$$\begin{array}{c} u=k_{p}\left(y_{s}-z_{1}\right)+k_{d}\left({\dot{y}}_{r}-z_{2}\right)+{\ddot{y}}_{r}-\frac{z_{3}}{b_{0}} \end{array}$$ (15)

where *k*_*p*_ and *k*_*d*_ are the proportional and differential coefficients in the PD module, respectively; *b*_0_ is the adjustable gain; *u* is the control signal outputted by the controller; *y*_*s*_ is the control signal of SMC; and *z*_1_, *z*_2_, and *z*_3_ are the state evaluations of the controlled object by LESO. *z*_1_ evaluates the output of the system, *z*_2_ evaluates the differential of the system’s output, and *z*_3_ evaluates the total disturbance received by the system, which can designed as follows:

$$\begin{array}{c} \left\{ \begin{array}{c} {\dot{z}}_{1}=z_{2}+\beta_{1}\left(y-z_{1}\right)\\ {\dot{z}}_{2}=z_{3}+\beta_{2}\left(y-z_{1}\right)+b_{0}u\\ {\dot{z}}_{3}=\beta \left(y-z_{1}\right) \end{array}\right. \end{array}$$ (16)

where *β*_1_, *β*_2_, and *β*_3_ represent the adjustable gains in LESO, and the values of the three adjustable gains are determined by the observed bandwidth *ω*_*o*_. The relationship between the adjustable gains and the observed bandwidth can be expressed as $\beta_{1}=3\omega_{o},\beta_{2}=3\omega_{o}^{2},\text{and}\;\beta_{2}=\omega_{o}^{3}$, and *y* is the output value of the system. And it is worth noting that the design of PD module and LESO module can refer to reference [9].

**Remark 1**: Linear extended state observer (LESO) is the core part of linear active disturbance rejection, it mainly takes the external interference and internal disturbance of the system as a new state variable, and then takes the input and output of the controlled object as the input of the LESO, and evaluates all the state variables of the control system through the state observer. Its characteristic polynomial can be expressed as Eq (17):

$$\begin{array}{c} D\left(s\right)=s^{3}+\beta_{1}s^{2}+\beta_{2}s+\beta_{3}={\left(s+\omega_{o}\right)}^{3} \end{array}$$ (17)

where $\beta =[3\omega_{o}\;3\omega_{o}^{2}\;\omega_{o}^{3}]$, and *ω*_*o*_ represents the observation bandwidth in the LESO module. The initial control signal *u*_0_ can be represented as Eq (18):

$$\begin{array}{c} u_{0}=k_{p}\left(y_{s}-z_{1}\right)+k_{d}\left({\dot{y}}_{r}-z_{2}\right)+{\dot{y}}_{r} \end{array}$$ (18)

### 3.3 Design of the adaptive parameter adjustment law

We have designed the following adaptive laws for parameters *k*_*p*_ and *k*_*d*_ in the PD module:

$$\begin{array}{c} {\dot{\hat{k}}}_{p}=[\delta k_{p}\left(y_{s}-z_{1}\right)\vartheta ]/{\tilde{k}}_{p} \end{array}$$ (19)

$$\begin{array}{c} {\dot{\hat{k}}}_{d}=[\begin{array}{c} \delta k_{d}\left({\dot{y}}_{r}-z_{2}\right)\zeta \end{array}]/{\tilde{k}}_{d} \end{array}$$ (20)

where *ϑ* and *ζ* are positive parameters, and *δ* is the error filter and can be represented as follows:

$$\begin{array}{c} \delta =[\begin{array}{cc} \alpha^{T} & 1 \end{array}]e_{2} \end{array}$$ (21)

where *e*_2_ = *y* − *y*_*r*_ represents the error between the input and output signals of the control system, and *α* = *t*_1_ is an appropriate coefficient. When *δ* → 0, it satisfies *e*_2_ → 0. The expression after differentiation of *δ* is as follows:

$$\begin{array}{c} \dot{\delta }={\dot{x}}_{2}-{\ddot{y}}_{r}+[\begin{array}{ccc} 0 & & \alpha^{T} \end{array}]e_{2}=-a\delta +k_{p}\left(y_{s}-z_{1}\right)+k_{d}\left({\dot{y}}_{r}-z_{2}\right) \end{array}$$ (22)

**Remark 2**: To prevent ${\tilde{k}}_{p}\;\text{and}\;{\tilde{k}}_{d}$ from being equal to zero, the expressions for ${\dot{\hat{k}}}_{p}\;\text{and}\;{\dot{\hat{k}}}_{d}$ are rewritten in integral form, where ${\dot{\hat{k}}}_{p}\;\text{and}\;{\dot{\hat{k}}}_{d}$ can be expressed as follows:

$$\begin{array}{c} {\hat{k}}_{p}=\int_{0}^{t}[\delta k_{p}\left(y_{s}-z_{1}\right)\vartheta ]/{\tilde{k}}_{p}d\tau +k_{p0} \end{array}$$ (23)

$$\begin{array}{c} {\hat{k}}_{d}=\int_{0}^{t}\left[\delta k_{d}\left(y_{r}-z_{2}\right)\zeta \right]/{\tilde{k}}_{d}d\tau +k_{d0} \end{array}$$ (24)

where ${\hat{k}}_{p}$ is the evaluation value of *k*_*p*_; ${\hat{k}}_{d}$ is the evaluation value of *k*_*d*_; and *k*_*p*0_ and *k*_*d*0_ are positive parameters. The existence of these two parameters is to ensure that ${\tilde{k}}_{p}=k_{p}-{\hat{k}}_{p}$ and ${\tilde{k}}_{d}=k_{d}-{\hat{k}}_{d}$ are not zero.

## 4 IWOA

Due to the presence of too many adjustable parameters in the controller, it is difficult to debug it through experience. Therefore, we introduce an improved whale algorithm to optimize the parameters of the control system: *b*_0_, *ω*_*o*_, *k*_*p*0_, *k*_*d*0_, *α*, *ζ*, *ϑ*, *k*_*p*_, *k*_*d*_.

### 4.1 Traditional whale algorithm

Whale algorithm is an intelligent optimization algorithm that evolves from the process of whale hunting. This algorithm has simple principles, easy operation, and strong stability. At the performance optimization level, the whale algorithm exhibits better solving accuracy and stability compared with other traditional algorithms. Whale predation adopts a bubble net predation method, which has the following three manifestations:

(1) Surround the prey

Whales can identify the position of their prey and surround it. This process is represented by the following equation:

$$\begin{array}{c} \vec{D}=|\begin{array}{cc} \vec{C} & \vec{X^{\prime }(t)}-\vec{X(t)} \end{array}| \end{array}$$ (25)

$$\begin{array}{c} \vec{X(t+1)}=\vec{X^{\prime }(t)}-\vec{A}\vec{D} \end{array}$$ (26)

where $\vec{A}\;\text{and}\;\vec{C}$ represent the coefficient vectors, and $\vec{X^{\prime }\left(t\right)}$ represents the position vector of the current optimal solution. The calculation method for $\vec{A}\;\text{and}\;\vec{C}$ is as follows:

$$\begin{array}{c} \vec{A}=\vec{2a}\vec{r_{1}}-\vec{a} \end{array}$$ (27)

$$\begin{array}{c} \vec{C}=2\vec{r_{2}} \end{array}$$ (28)

where $\vec{r_{1}}\;\text{and}\;\vec{r_{2}}$ represent random vectors between $\left[\begin{array}{c} 0,1 \end{array}\right]$. The traditional iterative method for $\vec{a}$ is to linearly decrease from two to zero.

(2) Bubble net attack

When the range of $\left|\vec{A}\right|$ is within $\left(\begin{array}{c} -1,1 \end{array}\right)$, whales will target their prey. Whales initiate an attack using a spiral encirclement method. The following two methods are used: 1. gradually approaches the prey; 2. spirally swims around the prey. Both methods simultaneously occur. The process is represented by the following mathematical expression:

$$\begin{array}{c} \vec{X(t+1)}=\{\begin{array}{cc} \vec{X^{\prime }(t)}-\vec{A}\vec{D}, & p<0.5\\ \vec{D}e^{bl}\;\text{cos}2\pi l+\vec{X^{\prime }(t)}, & p\geq 0.5 \end{array} \end{array}$$ (29)

where *l* and *b* are constants.

(3) Search for prey

When $|\vec{A}|\geq 1$, whales will move away from their prey to search for other targets, ensuring that they find the prey they need, which represents the global optimum.

$$\begin{array}{c} \vec{D}=|\vec{C}\vec{X_{s}(t)}-\vec{X(t)}| \end{array}$$ (30)

$$\begin{array}{c} \vec{X(t+1)}=\vec{X_{s}(t)}-\vec{A}\vec{D} \end{array}$$ (31)

where $\vec{X_{s}\left(t\right)}$ represents a random position vector.

### 4.2 Simulated annealing algorithm

Simulated annealing algorithm (SA algorithm) is derived from the principle of simulating metal annealing. This algorithm has a certain probability of accepting solutions that are worse than the current solution, enabling it to potentially escape local optima. The calculation process of the SA algorithm is simple and exhibits strong general robustness. This process can be outlined as follows:

(1) Initialization: initial temperature *T*, initial solution state *x* (iteration starting point), and number of iterations for each temperature state *L*.
(2) Generate new solution *x*_*new*_ = *x* + Δ*x*. Δ*x* is a random number within a range of increments.
(3) Calculate increment Δ*f* = *f* (*x*_*new*_) − *f* (*x*) and evaluate whether Δ*f* meets the requirements based on actual needs. If Δ*f* meets the requirements, then use *x*_*new*_ as the new solution; otherwise, use probability $p=e^{-\frac{\Delta f}{kT}}$ to accept *x*_*new*_ as the new solution.
(4) Determine whether the number of iterations at the current temperature has been reached. If the number of iterations has been met, then determine whether the termination condition is satisfied. If the condition is met, then terminate the process. If the number of iterations is not met, then continue iterating until the condition is satisfied.

If the number of times does not meet the termination condition, then continue to cool down. The temperature cooling process is *T* = *αT*, and *α* usually takes values close to 1, such as 0.95 and 0.9.

### 4.3 IWOA

The integration of the annealing algorithm into the whale algorithm results in an improved whale algorithm, addressing the problem of the whale algorithm being trapped in local optima and enhancing convergence speed and search accuracy. The specific enhancements are detained as follows:

(1) Considering the cumbersome calculation and excessive number of iterations of the WOA algorithm, we have improved the descent method of $\vec{a}$ in the WOA algorithm to enhanced the convergence speed. In the early stage, the descent speed of $\vec{a}$ must be increased due to the large range of whale prey search and the inclusion of the SA algorithm to jump out of local optima. In the later stage, the descent speed must be slowed down. The improved descent method of $\vec{a}$ is as follows:

$$\begin{array}{c} \vec{a}=2{\left(\frac{L-L_{\text{max}}}{L_{\text{max}}}\right)}^{2} \end{array}$$ (32)

where *L* represents the current number of iterations, and *L*_max_ represents the maximum number of iterations.

(2) When whales initiate bubble net attacks, the probability of defining spiral swimming and approaching prey is equal. However, in practice, whales have a higher probability of spiral swimming around the target and continuing to search for prey when the search range is large in the early stage. In the later stage, when the distance to the prey is relatively close, whales have a high probability of approaching the prey. Therefore, we will make the following improvements to the algorithm.

$$\begin{array}{c} \vec{X(t+1)}=\{\begin{array}{c} \vec{X^{\prime }(t)}-\vec{A}\vec{D},p<0.9-0.4\frac{L}{L_{\text{max}}}\\ \vec{D}e^{bl}\text{cos}2\pi l+\vec{X^{\prime }(t)},p\geq 0.9-0.4\frac{L}{L_{\text{max}}} \end{array} \end{array}$$ (33)

where *L* represents the current number of iterations, and *L*_*max*_ represents the maximum number of iterations.

(3) The termination condition of the simulated annealing algorithm is usually when the temperature drops to a low level or the error reaches the set requirement. This work comprehensively considers the iteration termination condition of the whale algorithm, changes the termination condition to the number of iterations, and improves the temperature drop process. The temperature drop must be related to the number of iterations. Considering the inverse proportional function relationship, the improved temperature drop process is expressed as follows:

$$\begin{array}{c} T=\frac{L_{\text{max}}}{2L} \end{array}$$ (34)

The fitness function expression we use is expressed as follows:

$$\begin{array}{c} J_{ITAE}=\int_{0}^{\infty }t\left|e\left(t\right)\right|dt \end{array}$$ (35)

**Fig 4:**
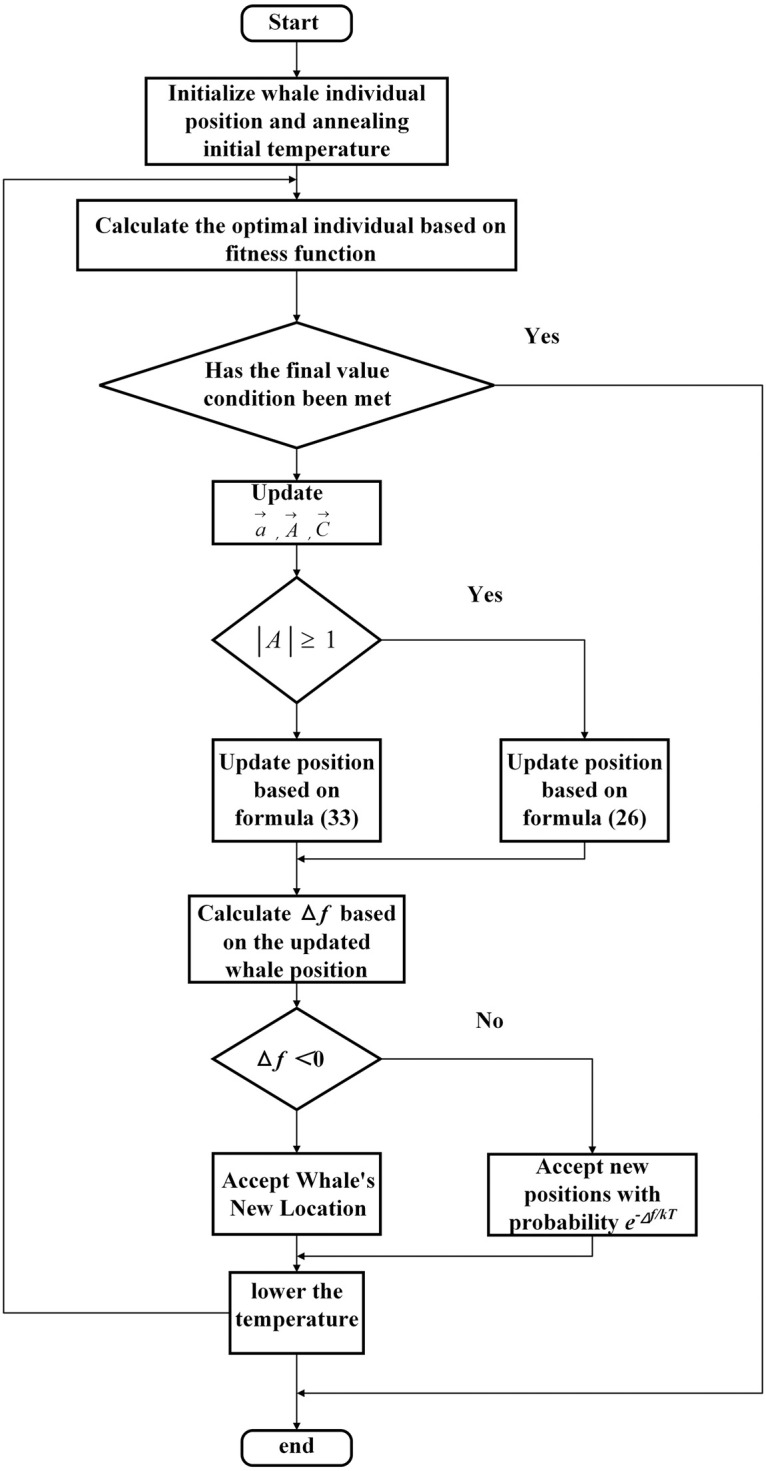
Improve algorithm process.

The improved algorithm flowchart is shown in Fig 4.

The improved algorithm operation steps are as follows:

**Step 1**: Initialize the whale population (quantity *m*), including the position of the whales, the given starting temperature *T*_0_, and the number of iterations *L*_*max*_, according to the constraint conditions of the temperature control system.
**Step 2**: Calculate the ITAE value of individual whales and change their positional coordinates.
**Step 3**: Determine if the end condition is met. If this condition is satisfied, then output the optimal value. Otherwise, proceed to Step 4.
**Step 4**: Update the algorithm-related parameters and determine whether $|\vec{A}|\geq 1$ is satisfied. Thereafter, update the whale position based on the algorithm’s relevant formula.
**Step 5**: Calculate the fitness value of the whale after the position update according to the fitness function, and compare it with before the update to calculate Δ*f*. If Δ*f* < 0 then accept the whale’s updated position. If Δ*f* ≥ 0 receives the updated position of the whale with probability $e^{\frac{-\Delta f}{kT}}$.
**Step 6**: Perform cooling operation.
**Step 7**: Return to step (2).

**Fig 5:**
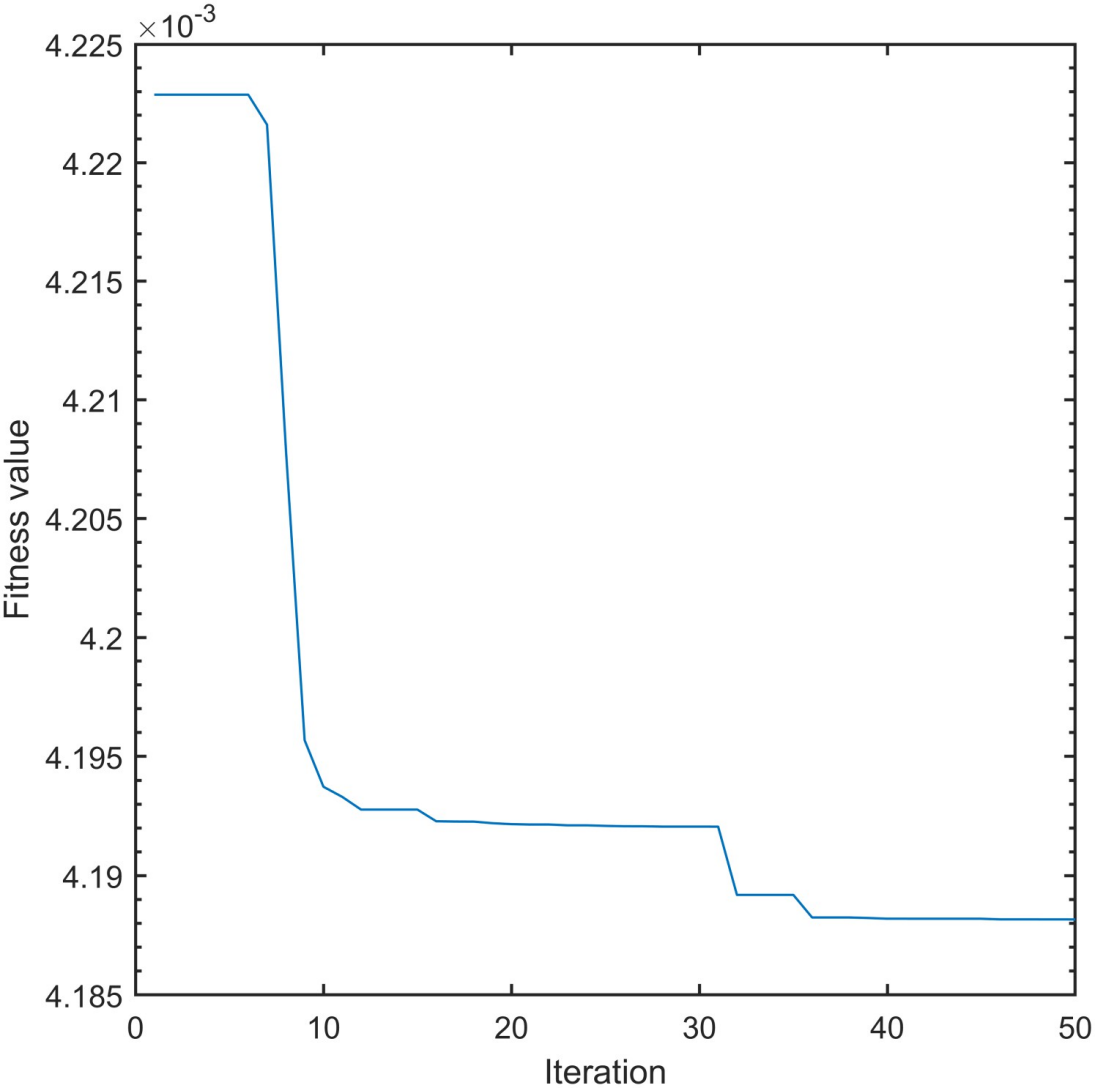
Convergence curve of unimproved fitness function.

**Fig 6:**
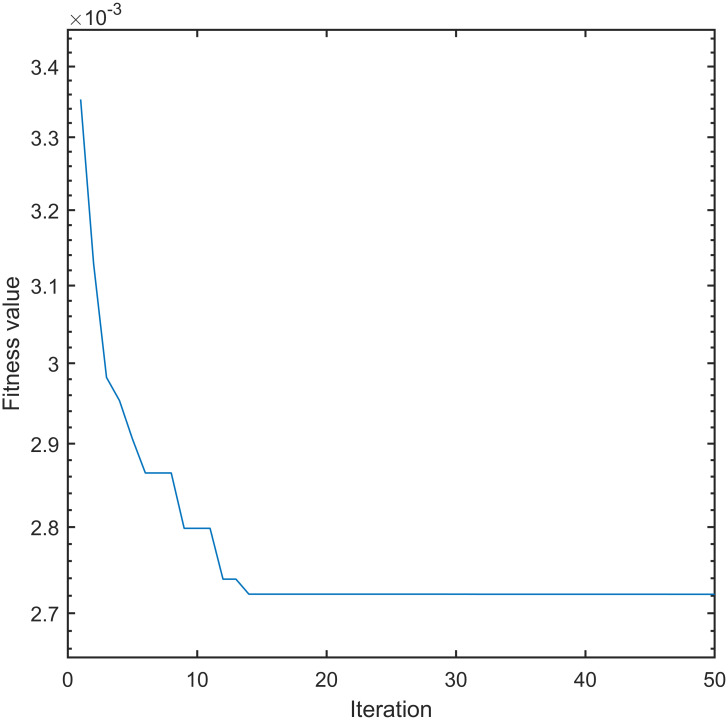
Improved fitness function convergence curve.

This study compares the convergence curves of the fitness function between the IWOA and the traditional whale algorithm:

Figs 5 and 6 compares the convergence curves of the fitness function between the IWOA and the traditional whale optimization algorithm:

Fig 5 depicts that the traditional whale optimization algorithm requires 36 iterations to reach the convergence state, and the fitness value eventually converges to 4.188 × 10^−3^. Fig 6 shows that the IWOA needs 14 iterations to reach the convergence state, and the fitness value finally converges to 2.722 × 10^−3^. The IWOA reduces the number of iterations by 61.11% compared with the traditional whale optimization algorithm. The fitness value decreased by 35.00%, indicating that the IWOA exhibits stronger search performance compared to traditional whale optimization algorithms, and it cannot easily fall into search blind spots.

## 5 Theoretical analysis

**Theorem1**: All signals in the system converge, and all errors are zero. where Lyapunov’s theorem is used to prove the convergence and stability of the system. The Lyapunov equation can be defined as follows:

$$\begin{array}{c} V=\frac{1}{2}s^{2}+\frac{1}{2}\delta^{2}+\frac{1}{2}{\tilde{k}}_{p}\vartheta {\tilde{k}}_{p}+\frac{1}{2}{\tilde{k}}_{d}\zeta {\tilde{k}}_{d} \end{array}$$ (36)

where *V* is a positive definite. The following expression can be obtained by differentiating the above formula:

$$\begin{array}{c} \dot{V}=s\dot{s}+\delta \dot{\delta }+{\tilde{k}}_{p}\vartheta {\dot{\tilde{k}}}_{p}+{\tilde{k}}_{d}\zeta {\dot{\tilde{k}}}_{d} \end{array}$$ (37)

Substituting Eqs (17) and (22) into Eq (37) yields:

$$\begin{array}{c} \dot{V}=s\left(w_{1}{\dot{e}}_{1}+{\ddot{e}}_{1}\right)+\delta [-a\delta +k_{p}\left(y_{s}-z_{1}\right)+k_{d}\left({\dot{y}}_{r}-z_{2}\right)]+{\tilde{k}}_{p}\vartheta^{-1}{\dot{\tilde{k}}}_{p}+{\tilde{k}}_{d}\zeta^{-1}{\dot{\tilde{k}}}_{d} \end{array}$$

$$\begin{array}{c} =s\left(w_{1}{\dot{e}}_{1}+{\ddot{z}}_{1}-{\ddot{y}}_{r}\right)-a\delta^{2}+\delta k_{p}\left(y_{s}-z_{1}\right)+\delta k_{d}\left({\dot{y}}_{r}-z_{2}\right)+{\tilde{k}}_{p}\vartheta^{-1}{\dot{\tilde{k}}}_{p}+{\tilde{k}}_{d}\zeta^{-1}{\dot{\tilde{k}}}_{d} \end{array}$$ (38)

$$\begin{array}{c} =s\left(w_{1}{\dot{e}}_{1}+z_{3}+y_{s}-{\ddot{y}}_{r}\right)-a\delta^{2}+\delta k_{p}\left(y_{s}-z_{1}\right)+\delta k_{d}\left({\dot{y}}_{r}-z_{2}\right)+{\tilde{k}}_{p}\vartheta^{-1}{\dot{\tilde{k}}}_{p}+{\tilde{k}}_{d}\zeta^{-1}{\dot{\tilde{k}}}_{d} \end{array}$$

Meanwhile, substituting Eq (18) into the above formula yields

$$\begin{array}{c} \dot{V}=s\left(w_{1}{\dot{e}}_{1}+z_{3}+{\ddot{y}}_{d}-w_{1}{\dot{e}}_{1}-z_{3}-w_{2}s-{\ddot{y}}_{r}\right)+\delta \lceil -a\delta +k_{p}\left(y_{s}-z_{1}\right)+k_{d}\left({\dot{y}}_{r}-z_{2}\right)\rceil +{\tilde{k}}_{p}\vartheta^{-1}{\dot{\tilde{k}}}_{p}+{\tilde{k}}_{d}\zeta^{-1}{\dot{\tilde{k}}}_{d} \end{array}$$

$$\begin{array}{c} =s\left(w_{1}{\dot{e}}_{1}+{\ddot{z}}_{1}-{\ddot{y}}_{r}\right)-a\delta^{2}+\delta k_{p}\left(y_{s}-z_{1}\right)+\delta k_{d}\left({\dot{y}}_{r}-z_{2}\right)+{\tilde{k}}_{p}\vartheta^{-1}{\dot{\tilde{k}}}_{p}+{\tilde{k}}_{d}\zeta^{-1}{\dot{\tilde{k}}}_{d} \end{array}$$

$$\begin{array}{c} =s\left(w_{1}{\dot{e}}_{1}+z_{3}+y_{s}-{\ddot{y}}_{r}\right)-a\delta^{2}+\delta k_{p}\left(y_{s}-z_{1}\right)+\delta k_{d}\left({\dot{y}}_{r}-z_{2}\right)+{\tilde{k}}_{p}\vartheta^{-1}{\dot{\tilde{k}}}_{p}+{\tilde{k}}_{d}\zeta^{-1}{\dot{\tilde{k}}}_{d} \end{array}$$ (39)

Eqs (19) and (20) are substituted into Eq (39):

$$\begin{array}{c} \dot{V}\leq -w_{2}s^{2}-a\delta^{2}+\delta k_{p}\left(y_{s}-z_{1}\right)+\delta k_{d}\left({\dot{y}}_{r}-z_{2}\right) \end{array}$$

$$\begin{array}{c} -{\tilde{k}}_{p}\vartheta^{-1}\left[\delta k_{p}\left(y_{s}-z_{1}\right)\vartheta \right]/{\tilde{k}}_{p}-{\tilde{k}}_{d}\zeta^{-1}\left[\delta k_{d}\left({\dot{y}}_{r}-z_{2}\right)\zeta \right]/{\tilde{k}}_{d} \end{array}$$

$$\begin{array}{c} \leq -w_{2}s^{2}-a\delta^{2}+\delta k_{p}\left(y_{s}-z_{1}\right)+\delta k_{d}\left({\dot{y}}_{r}-z_{2}\right)-\delta k_{p}\left(y_{s}-z_{1}\right)-\delta k_{d}\left({\dot{y}}_{r}-z_{2}\right) \end{array}$$ (40)

The following expression can be obtained by simplifying Eq (41):

$$\begin{array}{c} \dot{V}\leq -w_{2}s^{2}-a\delta^{2} \end{array}$$ (41)

where *w*_2_ and *a* are positive constants. Eq (41) can be written as follows:

$$\begin{array}{c} \dot{V}\leq 0 \end{array}$$ (42)

$\dot{V}$ is negatively definite due to the existence of *V* → ∞ in the presence of ∥s∥→∞and∥δ∥→∞. According to Lyapunov’s theorem, the system is stable.

Lyapunov equation is bounded according to Eq (42). We can obtain the following expression according to Barbarat’s theorem:

$$\begin{array}{c} \lim_{t\to \infty }\;s\left(t\right)=0,\underset{t\to \infty }{\text{lim}}\;\delta \left(t\right)=0 \end{array}$$ (43)

${\tilde{k}}_{p}\;\text{and}\;{\tilde{k}}_{d}$ are bounded according to Lyapunov theory, ensure that all signals in the system remain bounded. Consequently, the error is also bounded and tends toward zero.

## 6 Simulink result

**Fig 7:**
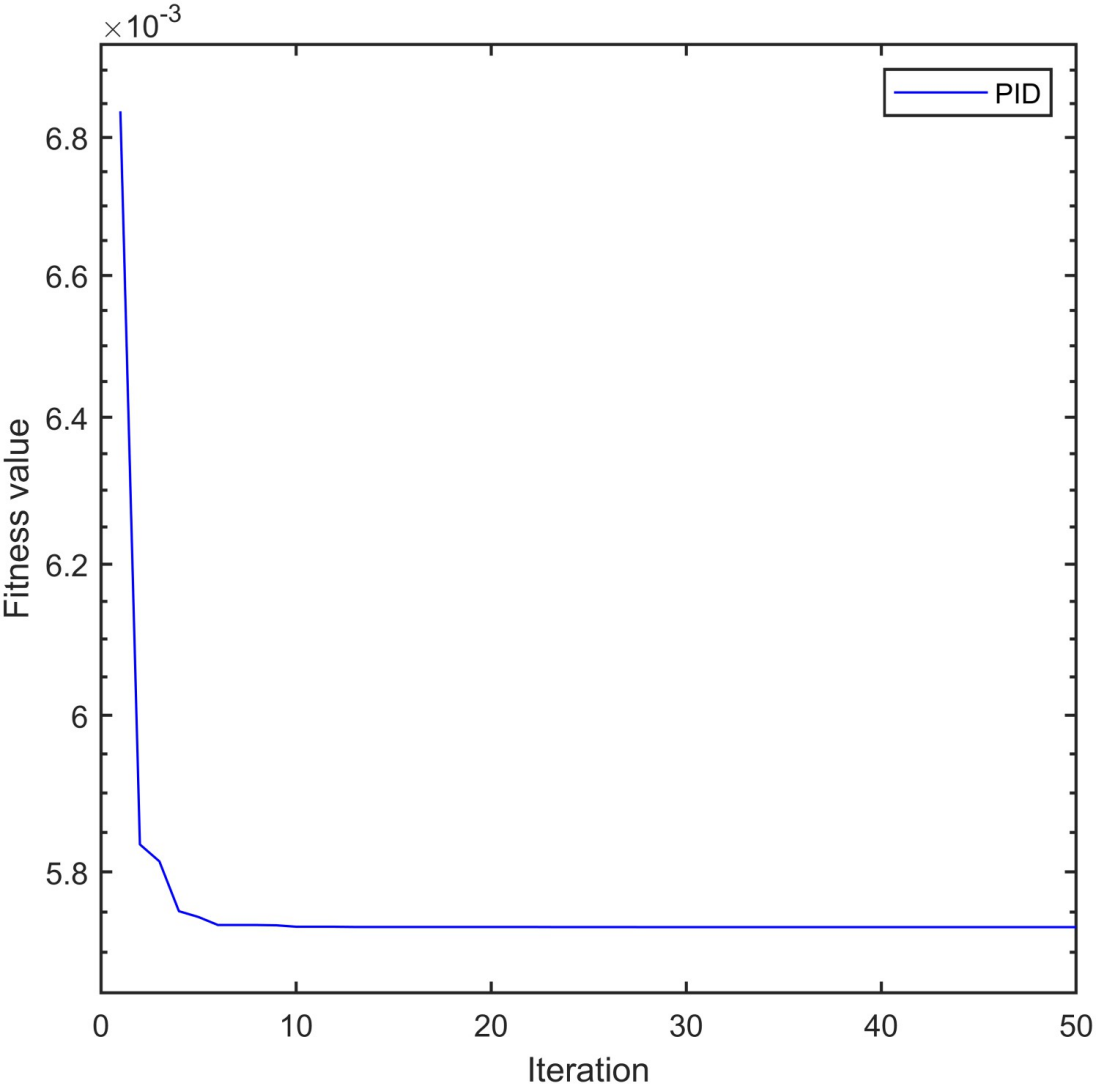
Convergence curve of PID fitness function.

**Fig 8:**
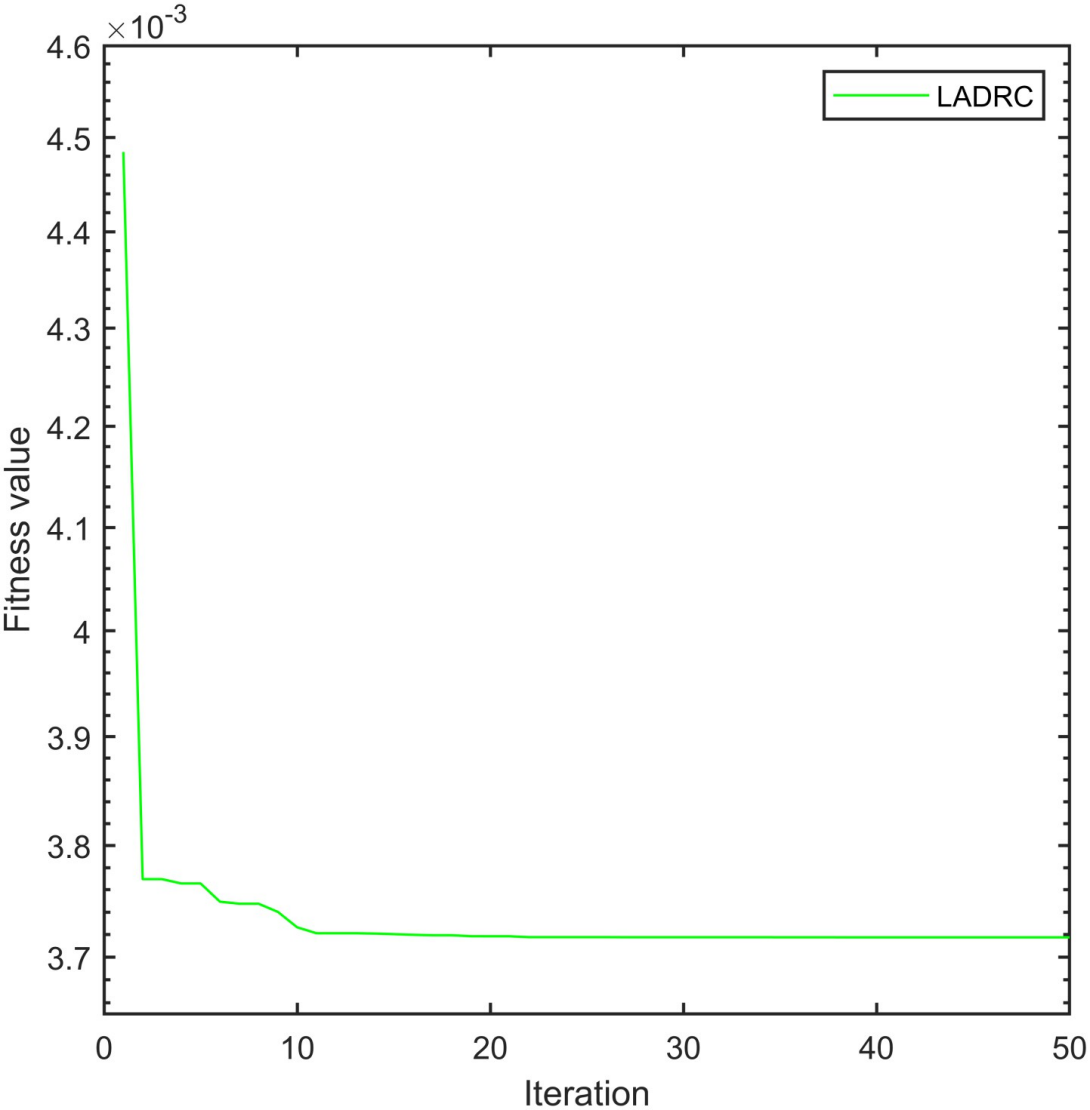
Convergence curve of LADRC fitness function.

**Fig 9:**
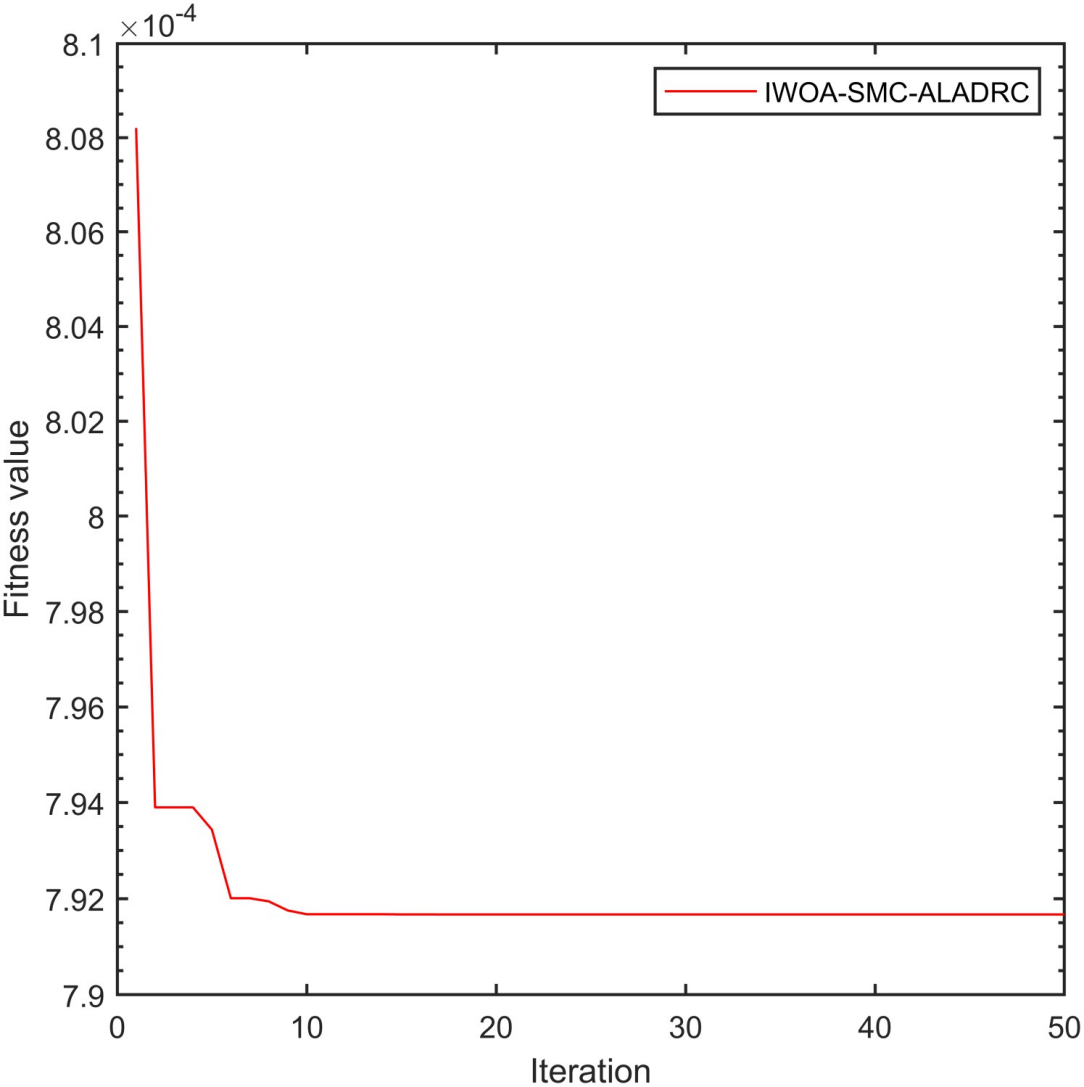
Convergence curve of IWOA-SMC-ALADRC fitness function.

To ensure fairness in simulation analysis, we use the same improved whale optimization algorithm to optimize PID and LADRC. Figs 7–9 show the convergence curves of the fitness functions of controller under the improved whale optimization algorithm:

From Fig 7, it can be seen that due to the limited number of adjustable parameters in PID, after optimizing the algorithm, the fitness function of PID converges after five iterations and ultimately converges to 5.731 × 10^−3^. Fig 8, it can be seen that LADRC converged to 3.717 × 10^−3^ after 10 iterations. From Fig 9, it can be seen that due to the presence of too many adjustable parameters, the fitness function of IWOA–SMC–ALADRC has undergone 10 iterations to reach a convergence state, and finally converges to 7.917 × 10^−4^. Compared with PID, the fitness function value of IWOA–SMC–ALADRC has decreased by 86.19% and decreased by 78.70% compared to LADRC. This indicates that after using the improved whale optimization algorithm to optimize the controller, IWOA–SMC–ALADRC produces the smallest error value, indicating that IWOA–SMC–ALADRC has the best control performance. At the same time, in order to better demonstrate the superiority of the proposed algorithm, we introduced two state-of-the-art control algorithms for single point maglev ball systems as comparison items. These two algorithms are: the cuckoo combined LADRC (CS-LADRC) algorithm proposed by Wei et al. [11] in 2022 and the improved LADRC algorithm proposed by Li et al. [31] in 2024.

This section mainly focuses on the simulation analysis of the disturbance resistance ability and tracking performance of the controller. We use three error criteria to evaluate the dynamic performance of the controllers. The three error criteria are as follows: integral absolute error (IAE), integral time multiplied absolute error (ITAE), and integral time multiplied square error (ITSE). The expression is as follows:

$$\begin{array}{c} IAE=\int_{0}^{t}|e_{y}|d\tau =\int_{0}^{t}|y_{r}-y_{out}|d\tau \end{array}$$ (44)

$$\begin{array}{c} ITAE=\int_{0}^{t}t|e_{y}|d\tau =\int_{0}^{t}t|y_{r}-y_{out}|d\tau \end{array}$$ (45)

$$\begin{array}{c} ITSE=\int_{0}^{t}t{\left|e_{y}\right|}^{2}d\tau =\int_{0}^{t}t{\left(y_{r}-y_{out}\right)}^{2}d \end{array}$$ (46)

Here *y*_*r*_ is the standard suspension air gap and *y*_*out*_ is the output suspension air gap of the system.

Perform the following simulation analysis by introducing PID and LADRC:

(1) Step signal analysis;
(2) Anti-disturbance analysis;
(3) Tracking performance analysis.

Table 2 shows the parameters used in the simulation analysis for the three control methods:

**Table 2:** Controller parameters used during the physical verification.

Controller	Parameter	Value	Parameter	Value	Parameter	Value
**PID**	*k*_*p*_	1500.2145	*k*_*i*_	2501.3642	*k*_*d*_	500.2358
**LADRC**	*b*_0_	3.5489	*ω*_*o*_	81.0245	*ω*_*c*_	30.2546
**IWOA-SMC-ALADRC**	*b*_0_	1.4266	*k*_*d*0_	0.0654	*ζ*	32.5489
	*ω*_*o*_	1512.1234	*k*_*p*_	4.2656	*α*	0.02689
	*k*_*p*0_	1300.2484	*k*_*d*_	2.3654	*ϑ*	42.5687

### 6.1 Step signal analysis

**Fig 10:**
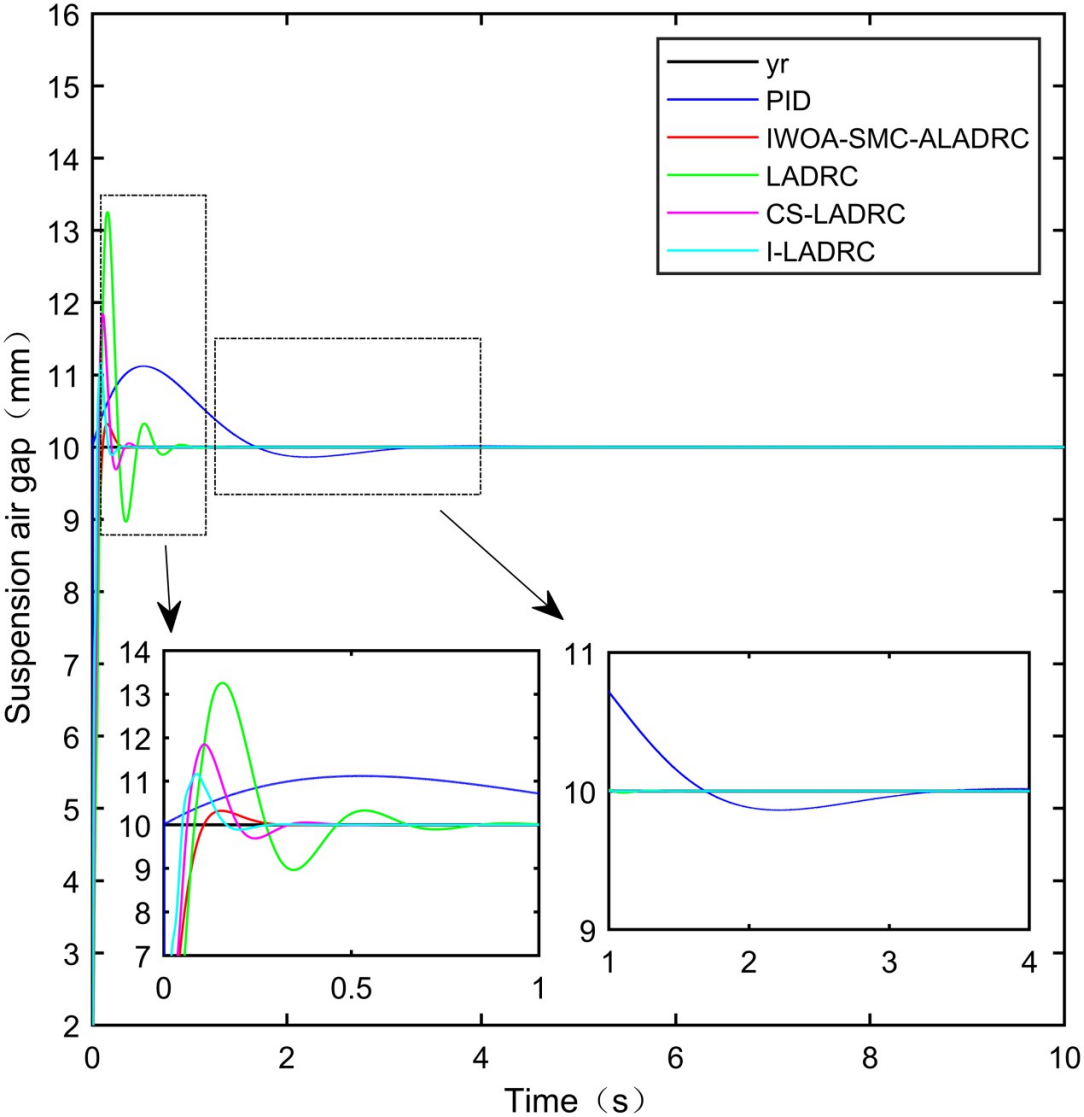
Step signal response.

The dynamic controller performance was verified by simulation. Fig 10 shows that when the standard suspension air gap is 10 mm, although the response speed of the PID output suspension air gap is fast, it has produced an overshoot of up to 12.54%. The adjustment time required to reach the standard suspension air gap is as high as 4.131 s. Although the adjustment time for the suspension air gap output by LADRC, CS-LADRC, and I-LADRC to reach the standard suspension air gap is shorter than that of PID, which are 1.204s, 0.522s, and 0.374s, respectively, the overshoot generated by these three controllers is 32.14%, 19.32%, and 12.61%, respectively. By contrast, the suspension air gap output by IWOA–SMC–ALADRC exhibits not only a fast response speed but also a small overshoot. The adjustment time to reach the standard suspension air gap is 0.281 s, and the generated overshoot is only 2.94%. Compared with PID, LADRC, CS-LADRC, and I-LADRC, the adjustment time of the suspension air gap output of IWOA-SMC-ALADRC to reach the standard suspension air gap was shortened by 93.20%, 76.66%, 46.17%, and 24.87%, respectively. Compared with PID, LADRC, CS-LADRC, and I-LADRC, the overshoot generated by the suspended air gap in the output of IWOA-SMC-ALADRC was reduced by 74.19%, 90.85%, 84.78%, and 76.69%, respectively. Therefore, IWOA-SMC-ALADRC has better control performance.

In terms of the IAE error data, compared to PID, LADRC, CS-LADRC, and I-LADRC, the error data of IWOA-SMC-ALADRC decreased by 50.36%, 34.09%, 21.76%, and 11.80%, respectively. In terms of the ITAE error data, the error data of IWOA-SMC-ALADRC decreased by 86.18%, 78.70%, 50.46%, and 16.02%, respectively. In terms of the ITSE error data, the error data of IWOA-SMC-ALADRC decreased by 86.14%, 65.46%, 85.75%, and 84.35%, respectively. This indicates that the dynamic performance of IWOA-SMC-ALADRC is superior to the other four control methods. The error values of the controller are shown in Table 3:

**Table 3:** Controller parameters used during the physical verification.

Reference signal	Controller	IAE	ITAE	ITSE
**Step**	PID	1.319 × 10^−3^	5.731 × 10^−3^	6.917 × 10^−7^
	LADRC	9.935 × 10^−4^	3.717 × 10^−3^	2.775 × 10^−7^
	IWOA-SMC-ALADRC	6.548 × 10^−4^	7.917 × 10^−4^	9.586 × 10^−8^
	CS-LADRC	8.369 × 10^−4^	1.598 × 10^−3^	6.725 × 10^−7^
	I-LADRC	7.424 × 10^−4^	9.427 × 10^−4^	6.124 × 10^−7^

### 6.2 Dynamic disturbance analysis

**Fig 11:**
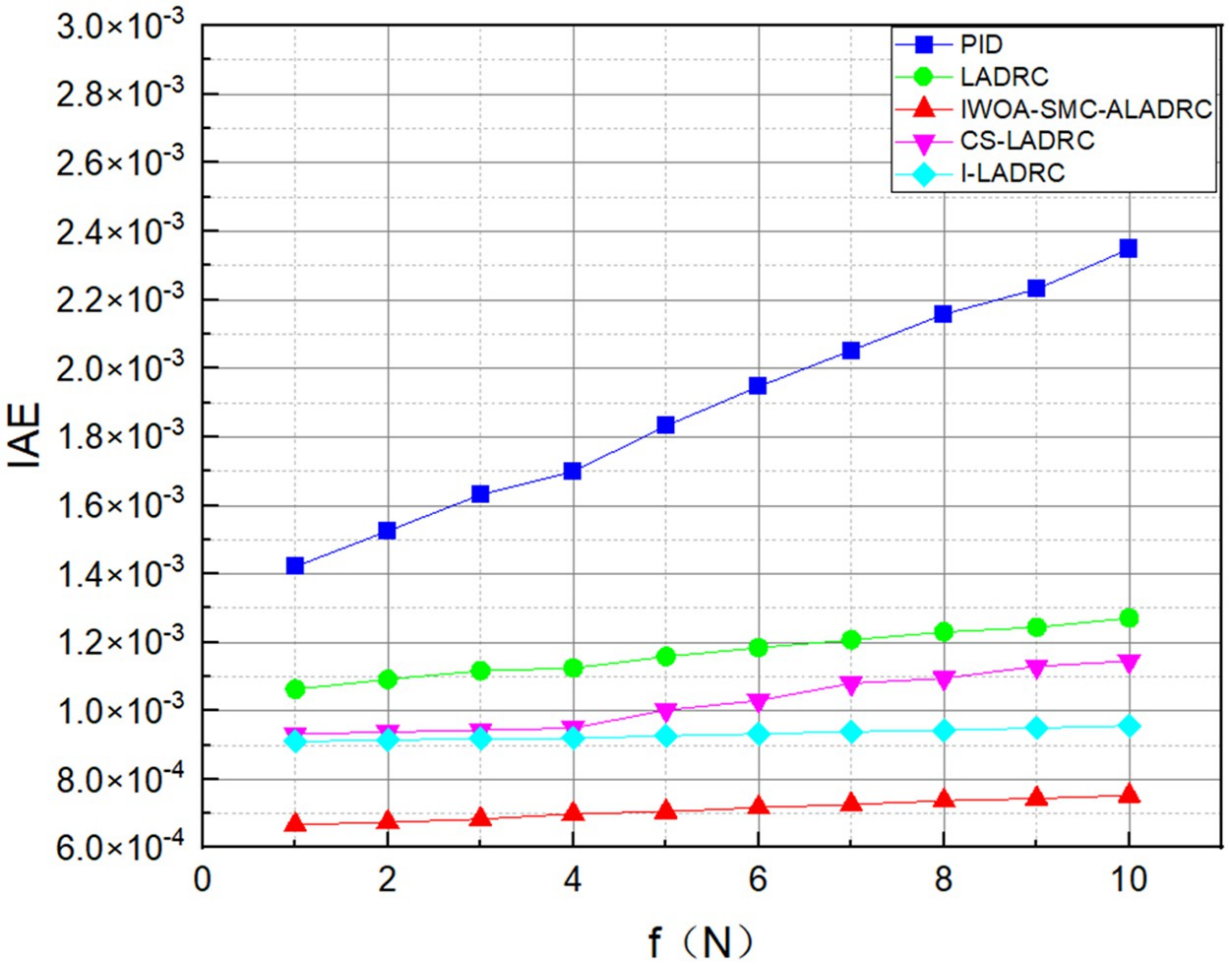
The trend of IAE error variation for five control methods.

**Fig 12:**
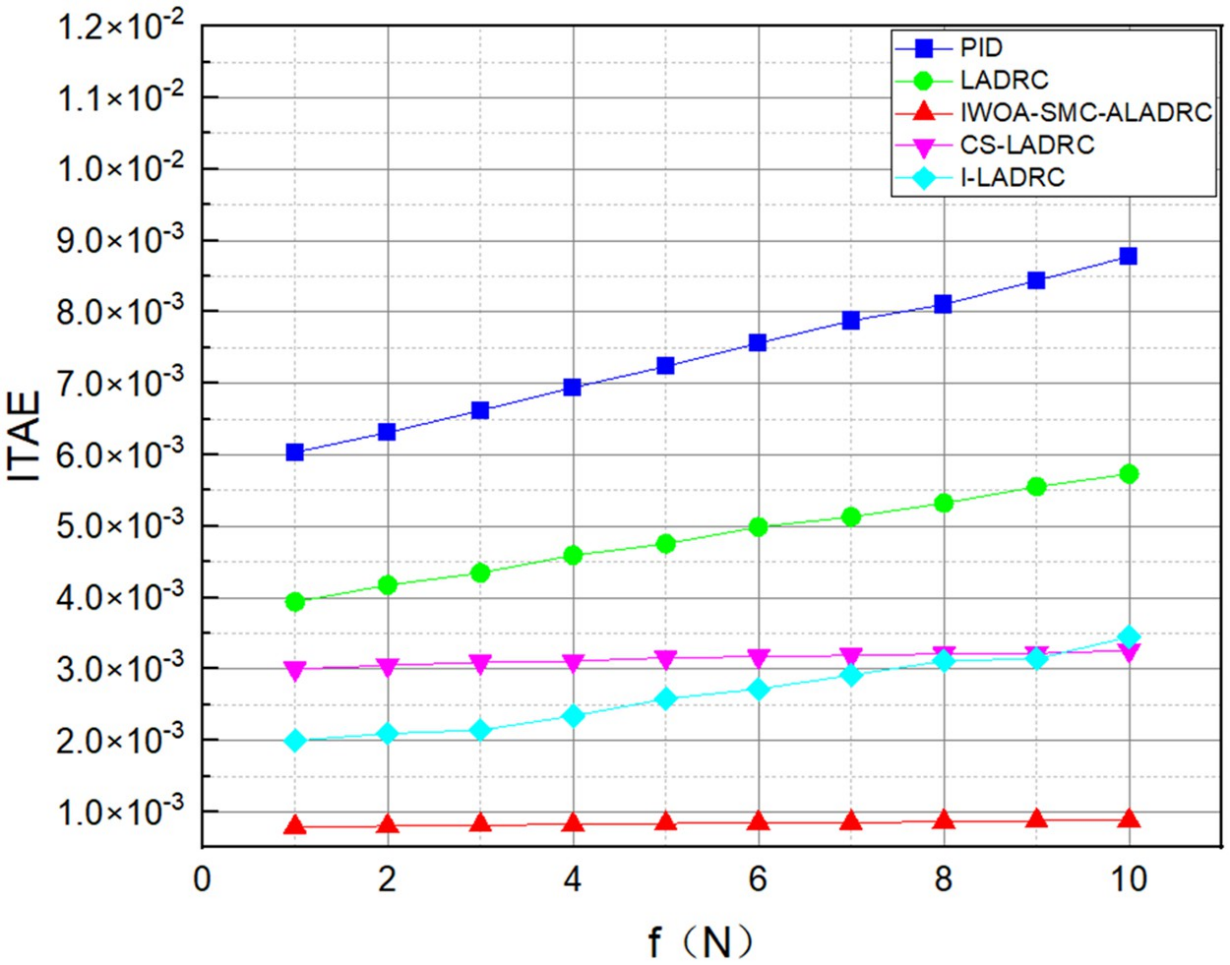
The trend of ITAE error variation for five control methods.

**Fig 13:**
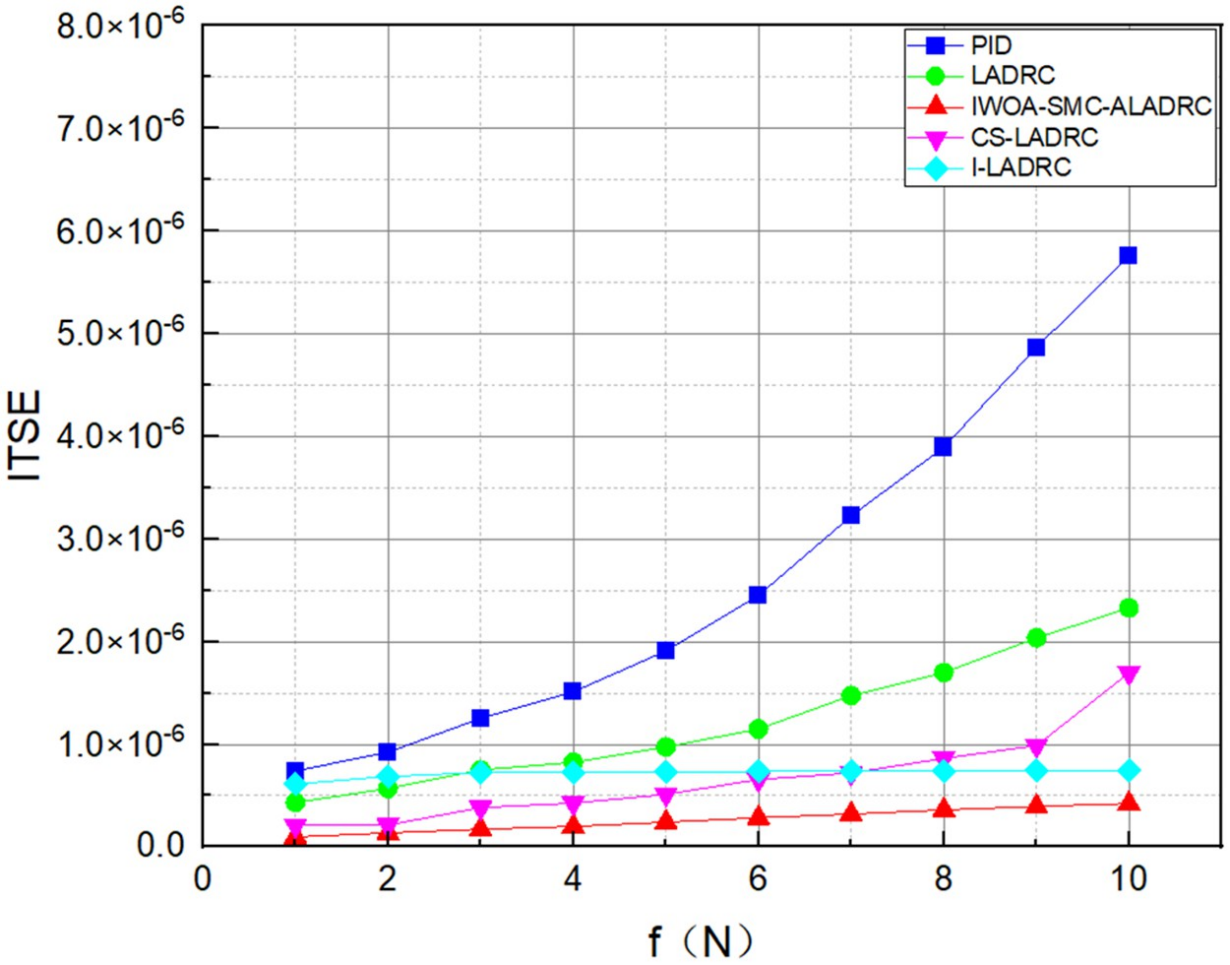
The trend of ITSE error variation for five control methods.

We applied disturbances of different intensities to the system to demonstrate its anti-disturbance performance. Figs 11–13 show the three error variation trends of the five control methods under various disturbance intensities.

**Fig 14:**
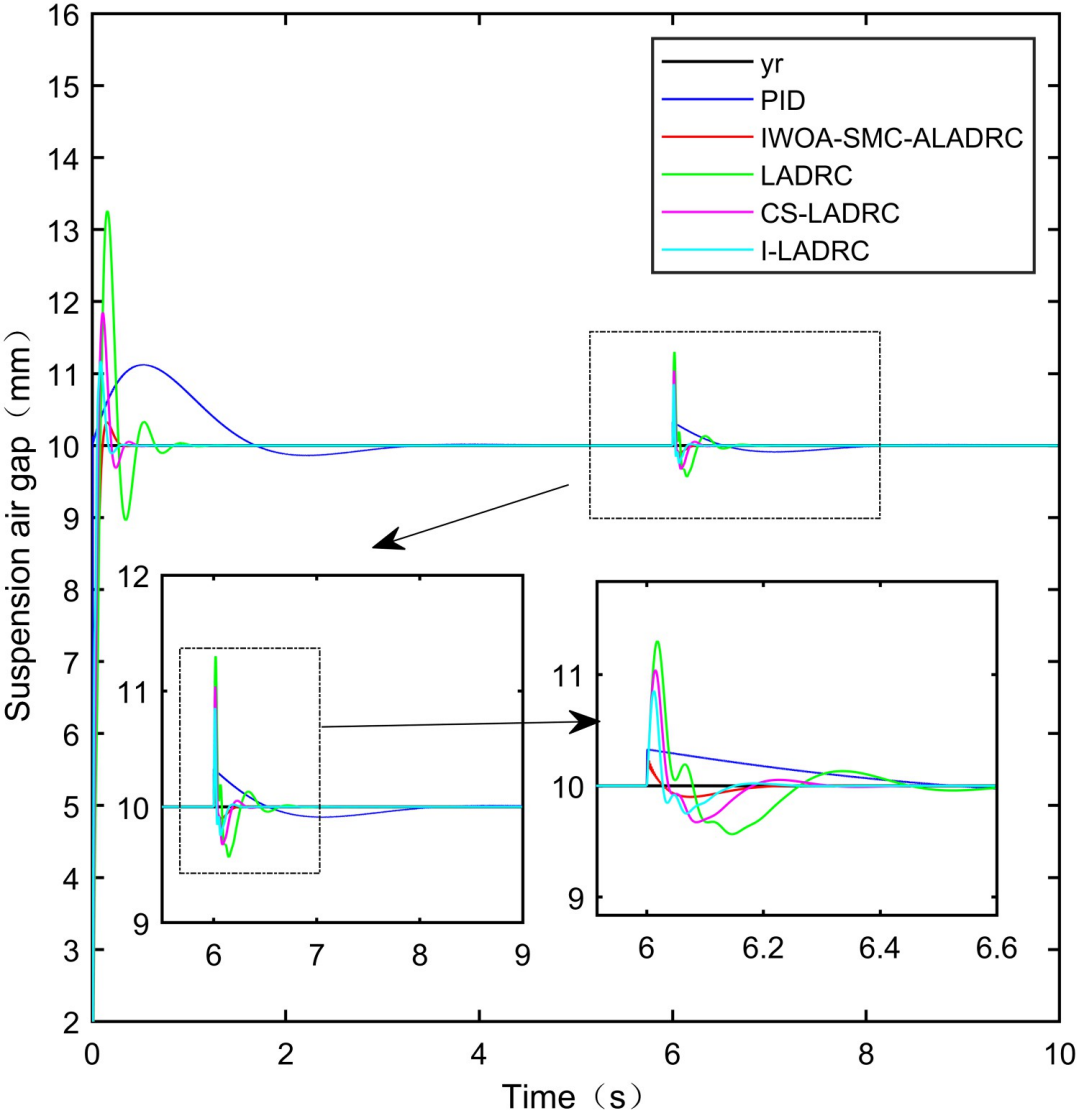
Step response curves for the disturbance intensities of 5 N.

**Fig 15:**
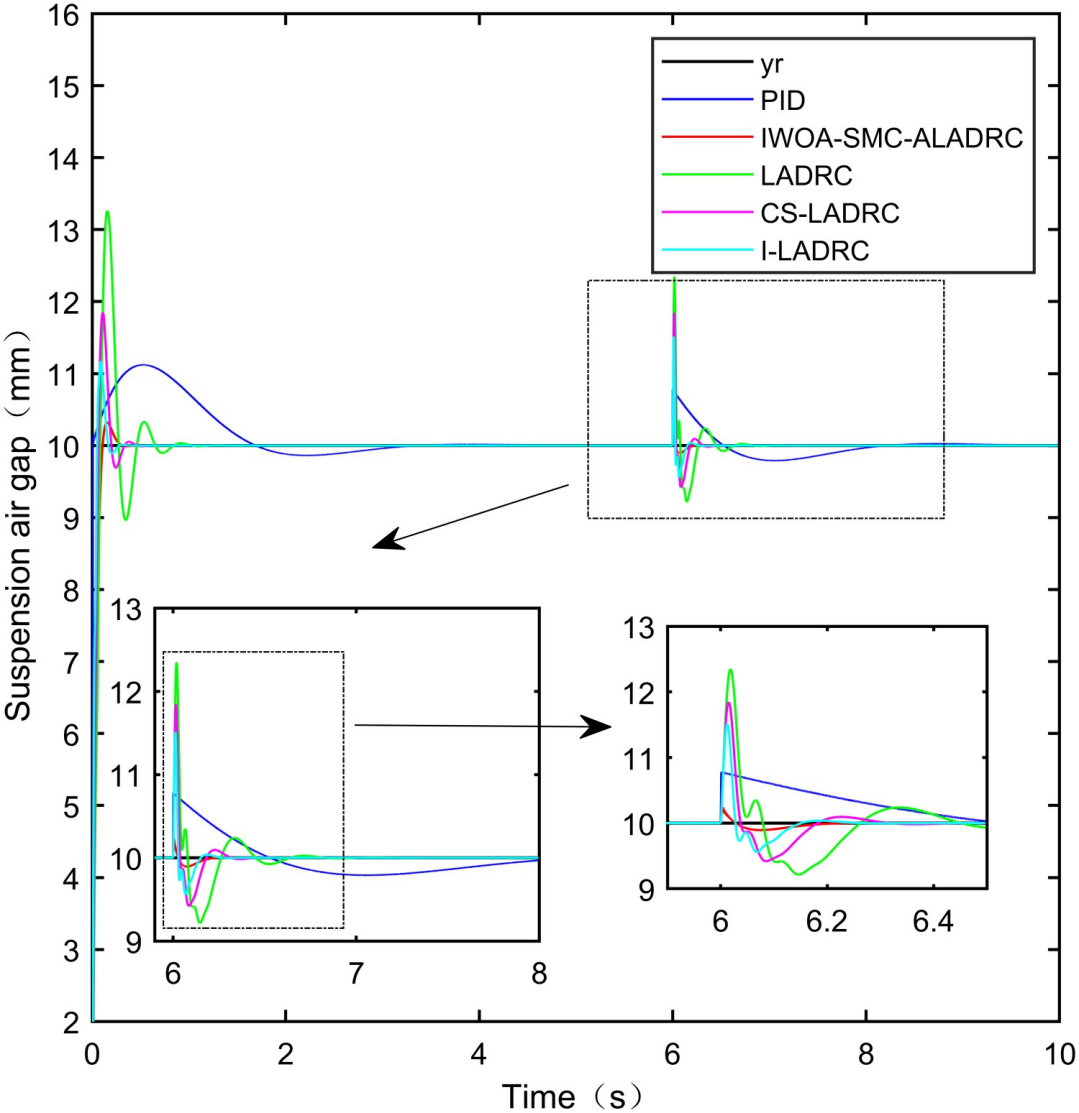
Step response curves for the disturbance intensities of 10 N.

Figs 11–13 show the trend of the three error indicators of PID, LADRC, CS-LADRC, I-LADRC, and IWOA–SMC–ALADRC as the disturbance intensity increases. It can be seen that the error values generated by IWOA-SMC-ALADRC are smaller than those generated by the other four control methods. In addition, with the increase of disturbance intensity, compared with the other four control methods, the error value generated by IWOA-SMC-ALADRC shows a relatively gentle increase trend. This indicates that IWOA-SMC-ALADRC has better anti-disturbance ability, and the greater the disturbance intensity, the more obvious it is. We selected disturbance intensities of 5 and 10 N for simulation analysis to more intuitively reflect the anti-disturbance performance of the controllers. The specific effects are shown in Figs 14 and 15.

From Fig 14, it can be seen that when the disturbance intensity applied to the system is 5N, the deviations of the suspended air gaps output by PID, LADRC, CS-LADRC, I-LADRC, and IWOA-SMC-ALADRC from the standard suspended air gap are 0.421mm, 1.423mm, 1.014mm, 0.772mm, and 0.107mm, respectively. Therefore, under 5N disturbance, compared with PID, LADRC, CS-LADRC, and I-LADRC, the distance between the suspended air gap output by IWOA-SMC-ALADRC and the standard suspended air gap decreased by 74.58%, 92.48%, 89.45%, and 86.14%, respectively. From Fig 15, it can be seen that when the disturbance intensity applied to the system is 10N, the deviations of the suspended air gaps output by PID, LADRC, CS-LADRC, I-LADRC, and IWOA-SMC-ALADRC from the standard suspended air gap are 0.876mm, 2.724mm, 1.793mm, 1.502mm, and 0.164mm, respectively. Therefore, under a disturbance of 10N, compared with PID and LADRC, the distance of the deviation of the suspension air gap output by IWOA-SMC-ALADRC from the standard suspension air gap was reduced by 81.28%, 93.98%, 90.85%, and 89.08%, respectively. According to the analysis of dynamic load disturbances, it can be concluded that IWOA–SMC–ALADRC has superior disturbance resistance ability.

From Table 4, it can be seen that when the disturbance intensity is 5N, in terms of the IAE error data, compared with PID, LADRC, CS-LADRC, and I-LADRC, the error data of IWOA-SMC-ALADRC have been reduced by 61.50%, 39.09%, 29.61%, and 23.87%, respectively. In terms of the ITAE error data, the error data of IWOA-SMC-ALADRC decreased by 87.81%, 82.19%, 73.19%, and 67.26%, respectively, in terms of the ITSE error data, the error values of IWOA-SMC-ALADRC decreased by 87.35%, 75.25%, 66.70%, and 64.15%, respectively. When the disturbance intensity is 10N, in terms of the IAE error data, compared to PID, LADRC, CS-LADRC, and I-LADRC, the error data of IWOA-SMC-ALADRC decreased by 67.94%, 40.75%, 34.22%, and 21.29%, respectively, while in terms of the ITAE error data, the error data of IWOA-SMC-ALADRC decreased by 89.81%, 84.40%, 72.54%, and 73.27%, respectively. In terms of the ITSE error data, the error data of IWOA-SMC-ALADRC decreased by 92.64%, 81.78%, 75.00%, and 43.11%, respectively.

**Table 4:** Error date of the step signal with disturbance.

Reference signal	Controller	IAE	ITAE	ITSE
**Step+Disturbance(5*N*)**	PID	1.832 × 10^−3^	6.944 × 10^−3^	1.911 × 10^−6^
	LADRC	1.158 × 10^−3^	4.754 × 10^−3^	9.767 × 10^−7^
	IWOA-SMC-ALADRC	7.053 × 10^−4^	8.466 × 10^−4^	2.417 × 10^−7^
	CS-LADRC	1.002 × 10^−3^	3.158 × 10^−3^	7.258 × 10^−7^
	I-LADRC	9.265 × 10^−4^	2.586 × 10^−3^	6.742 × 10^−7^
**Step+Disturbance(10*N*)**	PID	2.349 × 10^−3^	8.776 × 10^−3^	5.762 × 10^−6^
	LADRC	1.271 × 10^−3^	5.732 × 10^−3^	2.329 × 10^−6^
	IWOA-SMC-ALADRC	7.531 × 10^−4^	8.942 × 10^−4^	4.243 × 10^−7^
	CS-LADRC	1.145 × 10^−3^	3.258 × 10^−3^	1.697 × 10^−6^
	I-LADRC	9.568 × 10^−4^	3.345 × 10^−3^	7.458 × 10^−7^

### 6.3 Sine tracking analysis

This section simulates and analyses the tracking performance of the system is simulated and analysed when the standard suspension air gap changes in a sinusoidal function. The amplitude of the sine function is 2 mm, and the frequency is 5 Hz. The mathematical expression can be expressed using Eq (47). We can obtain the following expression according to Barbarat’s theorem:

$$\begin{array}{c} y_{r}=10+\text{sin}\left(5t\right) \end{array}$$ (47)

**Fig 16:**
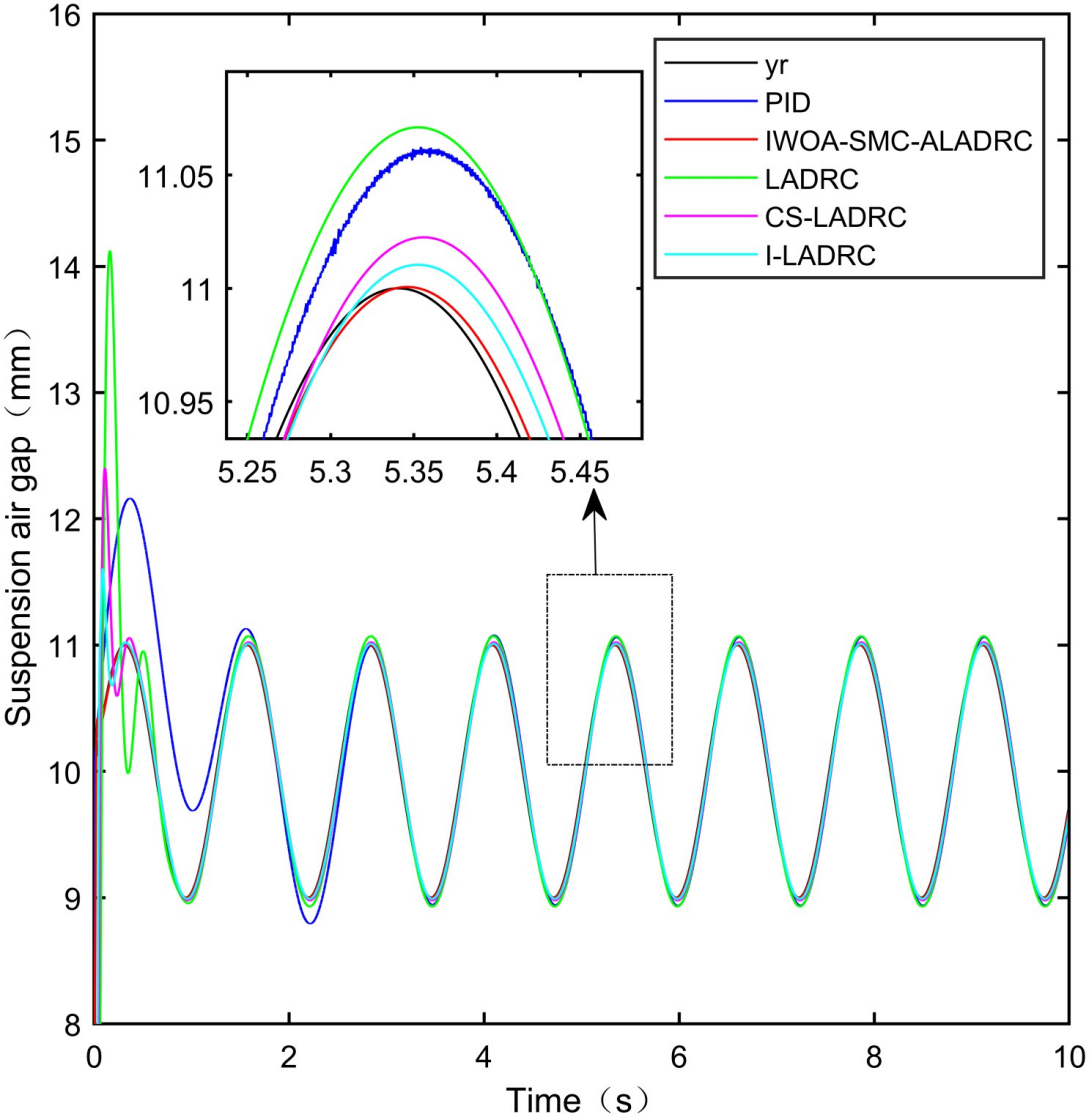
Performance analysis of sinusoidal signal tracking without disturbance.

**Fig 17:**
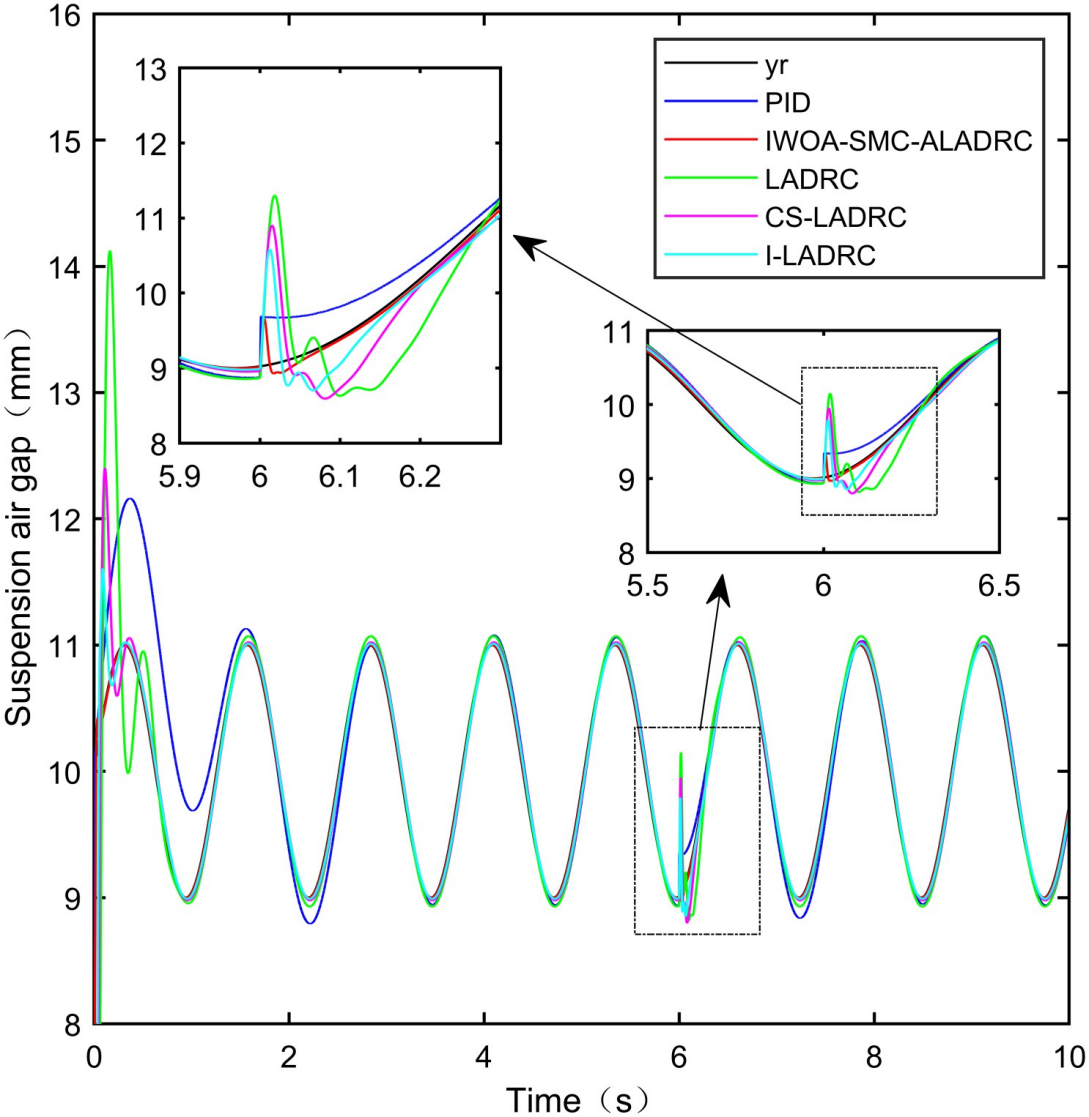
Performance Analysis of sinusoidal signal tracking with added disturbance.

This notion indicates that the suspended air gap varies in a sine function pattern within the range of 9–11 mm. The sine tracking effects of the five control modes are shown in Figs 16 and 17. It is worth noting that Fig 16 shows the tracking effect without disturbance, and Fig 17 shows the tracking effect with disturbance added.

From Fig 16, it can be seen that IWOA-SMC-ALADRC can approach the standard suspension air gap to the maximum extent compared with the other four control methods when no disturbance is applied to the system. In terms of the IAE error data, compared with PID, LADRC, CS-LADRC, and I-LADRC, the error data of IWOA-SMC-ALADRC decreased by 50.11%, 41.84%, 9.38%, and 1.76%, respectively. In terms of the ITAE error data, the error data of IWOA-SMC-ALADRC decreased by 95.40%, 93.71%, 92.32%, and 86.75%, respectively. In terms of ITSE error data, the error data of IWOA-SMC-ALADRC decreased by 88.99%, 78.35%, 48.46%, and 12.07%, respectively. Therefore, both simulation results and data indicate that IWOA-SMC-ALADRC has better tracking performance compared to the other four control methods.

From Fig 17, it can be seen that after adding disturbance, IWOA-SMC-ALADRC outperforms the other four control methods in both tracking performance and anti-disturbance performance. In terms of the IAE error, compared with PID, LADRC, CS-LADRC, and I-LADRC, the error data of IWOA-SMC-ALADRC decreased by 49.53%, 42.55%, 7.29%, and 2.24%, respectively. In terms of the ITAE error, the error data of IWOA-SMC-ALADRC decreased by 95.14%, 93.78%, 92.11%, and 91.19%, respectively. In terms of the ITSE error, the error data of IWOA-SMC-ALADRC have decreased by 91.19%, 81.49%, 66.69%, and 49.60%, respectively. In terms of the disturbance error, the disturbance error values of PID, LADRC, CS-LADRC, I-LADRC, and IWOA-SMC-ALADRC are 0.731mm, 2.221mm, 1.834mm, 1.624mm, and 0.712mm, respectively. Compared with PID, LADRC, CS-LADRC, and I-LADRC, the deviation distance of IWOA-SMC-ALADRC has been reduced by 2.60%, 67.94%, 61.18%, and 56.08%. It can be seen that after adding disturbance, the tracking performance and anti-disturbance ability of IWOA-SMC-ALADRC are superior to the other four control methods.

The specific error values generated by the three control methods are shown in Table 5.

**Table 5:** Error date of the sine signal.

Reference signal	Controller	IAE	ITAE	ITSE
**Sine**	PID	1.793 × 10^−2^	4.322 × 10^−2^	9.726 × 10^−6^
	LADRC	1.538 × 10^−2^	3.158 × 10^−2^	4.948 × 10^−6^
	IWOA-SMC-ALADRC	8.945 × 10^−3^	1.986 × 10^−3^	1.071 × 10^−6^
	CS-LADRC	9.871 × 10^−3^	2.585 × 10^−2^	2.078 × 10^−6^
	I-LADRC	9.105 × 10^−3^	1.938 × 10^−2^	1.218 × 10^−6^
**Sine+Disturbance(5*N*)**	PID	1.826 × 10^−2^	4.554 × 10^−2^	1.215 × 10^−5^
	LADRC	1.604 × 10^−2^	3.559 × 10^−2^	5.785 × 10^−6^
	IWOA-SMC-ALADRC	9.215 × 10^−3^	2.215 × 10^−3^	1.071 × 10^−6^
	CS-LADRC	9.940 × 10^−3^	2.808 × 10^−2^	3.215 × 10^−6^
	I-LADR	9.426 × 10^−3^	2.083 × 10^−2^	2.125 × 10^−6^

### 6.4 Square tracking analysis

$$\begin{array}{c} y_{r}=\{\begin{array}{cc} 12mm & \;k\leq t\leq k+1(k=0,2,4......)\\ 10mm & \;k\leq t\leq k+1(k=1,3,5......) \end{array} \end{array}$$ (48)

**Fig 18:**
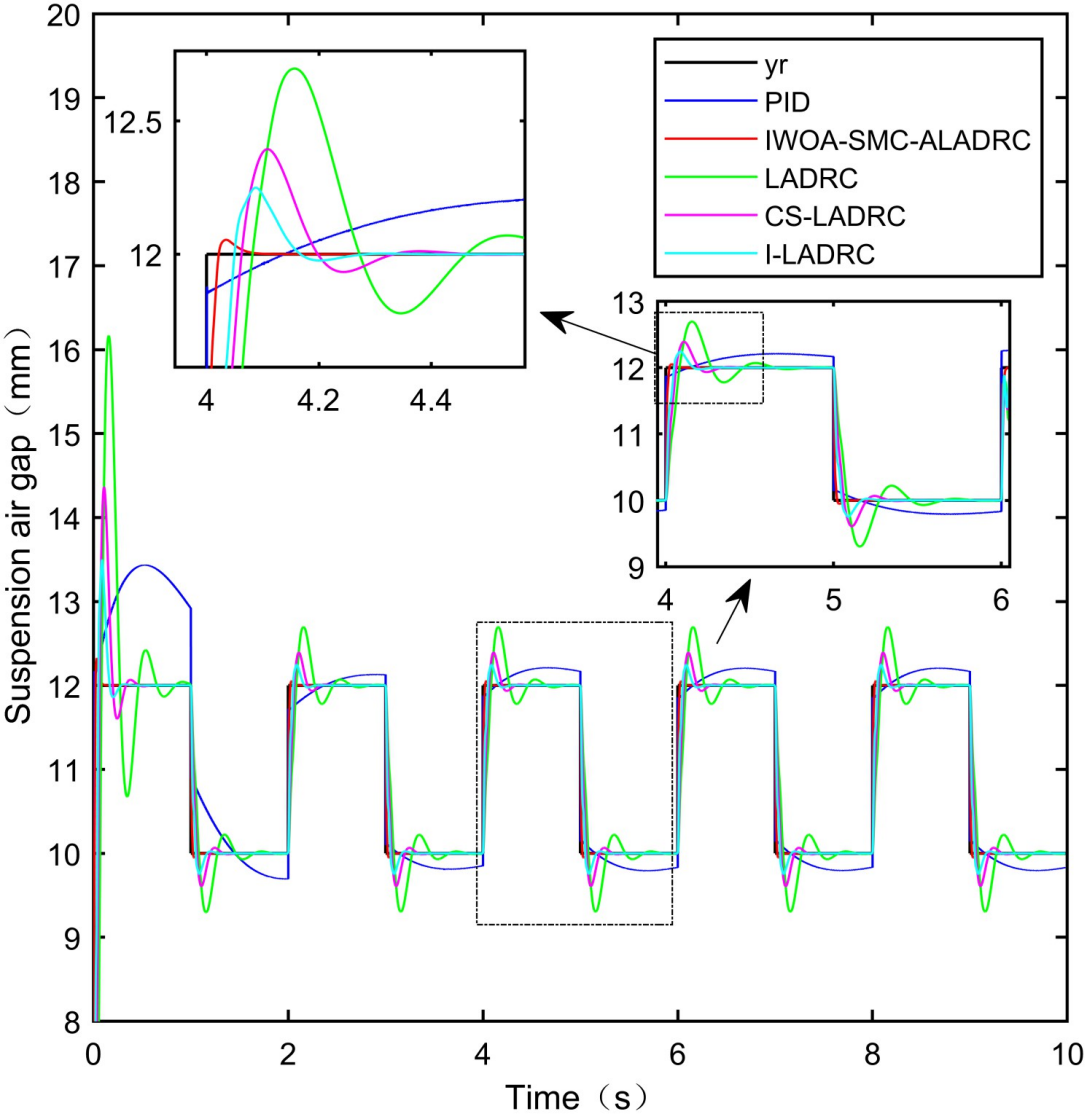
Performance analysis of tracking square wave signals without disturbance.

**Fig 19:**
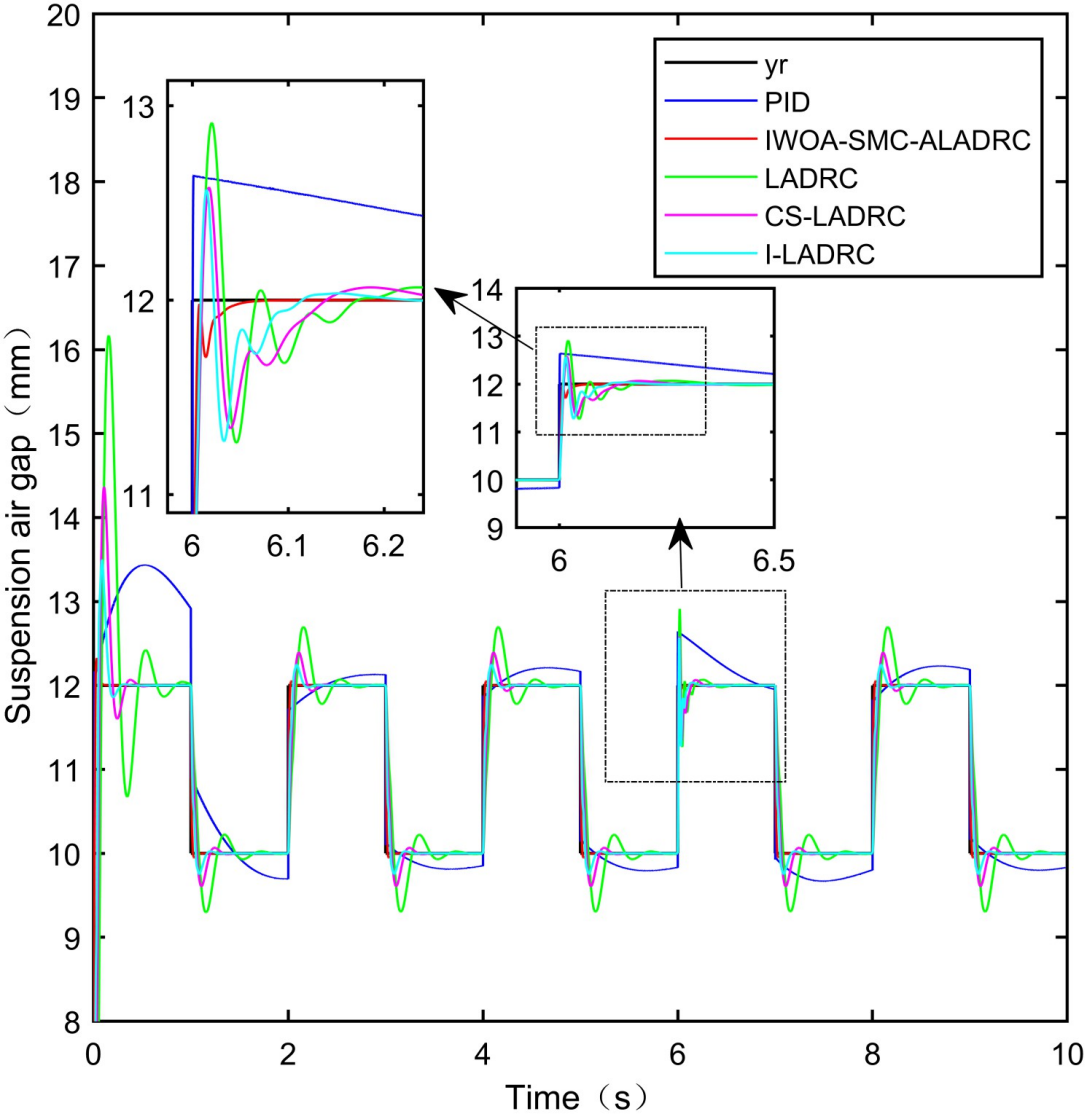
Performance analysis of tracking square wave signals with added disturbance.

When the standard suspended air gap varies with the square wave mode, the tracking effects of the five control methods are shown in Figs 18 and 19 due to the jumping and non-differentiability of the square wave signal. It is worth noting that Fig 18 shows the tracking effect without disturbance, and Fig 19 shows the tracking effect with 5N disturbance added at the 6th second.

As shown in Fig 18, when the system is not affected by disturbance, compared with the other four control methods, IWOA-SMC-ALADRC can approach the standard suspension air gap to the maximum extent. In the aspect of IAE error data, Compared with PID, LADRC, CS-LADRC, and I-LADRC, the error of IWOA-SMC-ALADRC was reduced by 75.44%, 78.95%, 55.17%, and 29.96%, respectively. In terms of the ITAE error data, the error data of IWOA-SMC-ALADRC decreased by 76.59%, 79.86%, 55.90%, and 25.38%, respectively, in terms of ITSE error data, the error data of IWOA-SMC-ALADRC decreased by 45.90%, 86.70%, 74.70%, and 56.89%, respectively. Therefore, both simulation results and data demonstrate that IWOA-SMC-ALADRC can approach the standard suspension air gap to the maximum extent compared with the other four control methods.

As shown in Fig 19, after the system is disturbed, compared with the other four control methods, IWOA-SMC-ALADRC can approach the standard suspension air gap to the maximum extent, and the deviation distance after disturbance is also much smaller than the other four control methods. In terms of the IAE error, compared with PID, LADRC, CS-LADRC, and I-LADRC, the error data of IWOA-SMC-ALADRC decreased by 70.91%, 76.30%, 44.36%, and 11.55%, respectively. In terms of the ITAE error, the error data of IWOA-SMC-ALADRC decreased by 97.95%, 98.01%, 95.08%, and 18.26%, respectively, in terms of ITSE error, the error data of IWOA-SMC-ALADRC decreased by 16.03%, 78.31%, 57.14%, and 27.19%, respectively. In terms of disturbance error, the deviation distances of PID, LADRC, CS-LADRC, I-LADRC, and IWOA-SMC-ALADRC are 0.663mm, 0.811mm, 0.612mm, 0.610mm, and 0.314mm, respectively. Compared to PID, LADRC, CS-LADRC, and I-LADRC, the deviation error of IWOA-SMC-ALADRC was reduced by 52.64%, 61.28%, 48.69%, and 48.52%, respectively. Therefore, after applying disturbance to the system, both simulation results and data can demonstrate that IWOA-SMC-ALADRC has better tracking performance and anti-disturbance ability.

The specific error values generated by the five control methods are shown in Table 6:

**Table 6:** Error date of the square signal.

Reference signal	Controller	IAE	ITAE	ITSE
**Square**	PID	2.552 × 10^−2^	8.100 × 10^−2^	1.711 × 10^−6^
	LADRC	2.977 × 10^−2^	9.415 × 10^−2^	6.961 × 10^−6^
	IWOA-SMC-ALADRC	6.267 × 10^−3^	1.896 × 10^−2^	9.256 × 10^−7^
	CS-LADRC	1.398 × 10^−2^	4.293 × 10^−2^	3.658 × 10^−6^
	I-LADRC	8.948 × 10^−3^	2.541 × 10^−2^	2.147 × 10^−6^
**Square+Disturbance(5*N*)**	PID	2.787 × 10^−2^	9.688 × 10^−2^	1.840 × 10^−6^
	LADRC	3.421 × 10^−2^	9.985 × 10^−2^	7.124 × 10^−6^
	IWOA-SMC-ALADRC	8.107 × 10^−3^	1.987 × 10^−2^	1.545 × 10^−6^
	CS-LADRC	1.457 × 10^−2^	4.042 × 10^−2^	3.605 × 10^−6^
	I-LADR	9.166 × 10^−3^	2.431 × 10^−2^	2.122 × 10^−6^

## 7 Experimental verification

**Fig 20:**
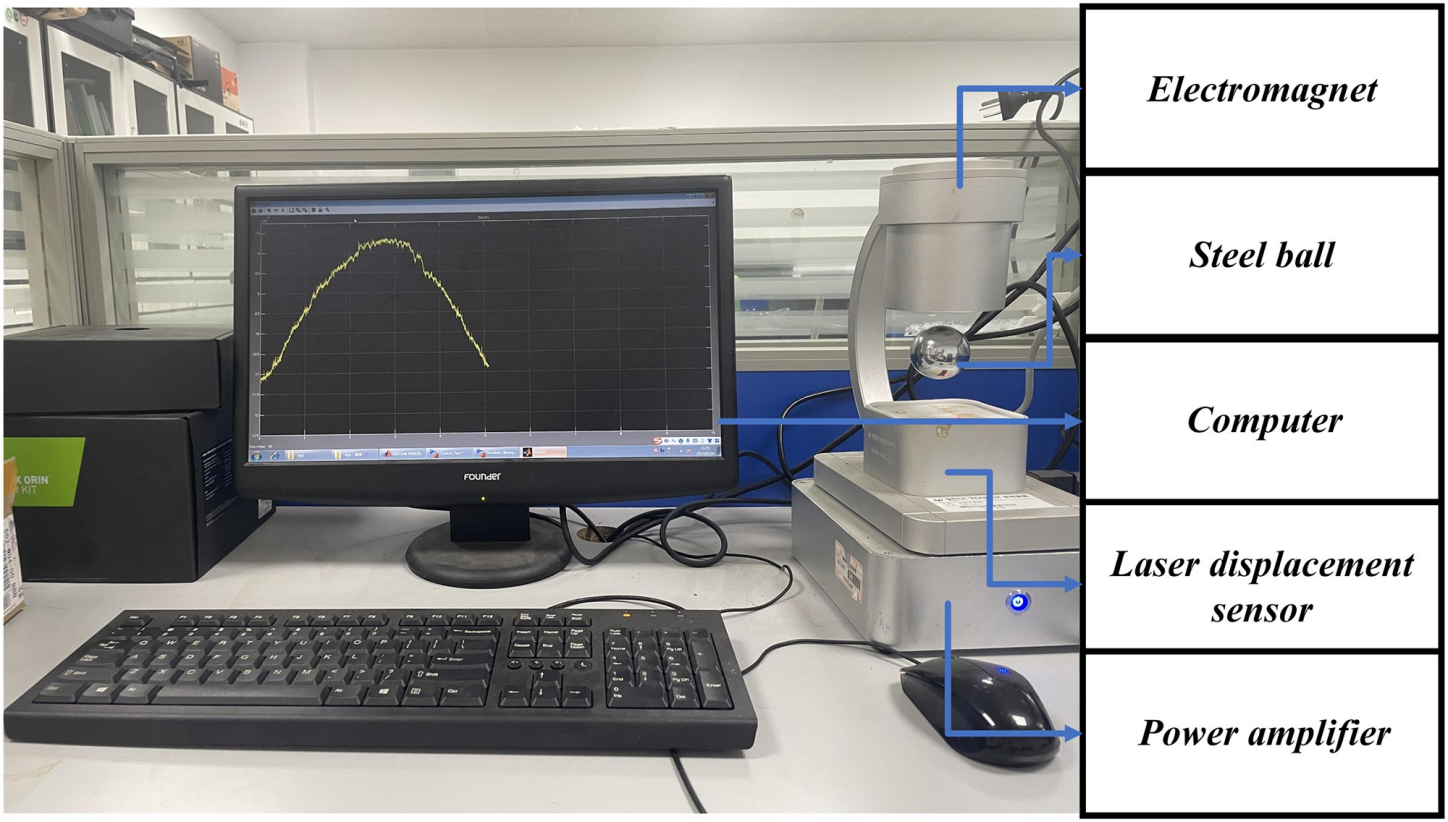
Electromagnetic levitation ball experimental platform.

Fig 20 depicts the GML2001 electromagnetic levitation experimental equipment produced by Guntech with a sampling frequency of 1000 Hz.

Due to the use of ignoring higher-order terms in the process of establishing mathematical models, there may be differences between simulation and actual control systems. Therefore, when conducting experiments, it is necessary to further adjust the controller parameters based on simulation parameter values.

The specific experimental controller parameters are shown in Table 7.

**Table 7:** Controller parameters used during the physical verification.

Controller	Parameter	Value	Parameter	Value	Parameter	Value
**PID**	*k*_*p*_	200	*k*_*i*_	2501.3642	*k*_*d*_	500.2358
**LADRC**	*b*_0_	6	*ω*_*o*_	81.0245	*ω*_*c*_	30.2546
**IWOA-SMC-ALADRC**	*b*_0_	4	*k*_*d*0_	0.0654	*ζ*	32.5489
	*ω*_*o*_	1512.1234	*k*_*p*_	4.2656	*α*	0.02689
	*k*_*p*0_	1300.2484	*k*_*d*_	2.3654	*ϑ*	44

### 7.1 Step signal experiment

**Fig 21:**
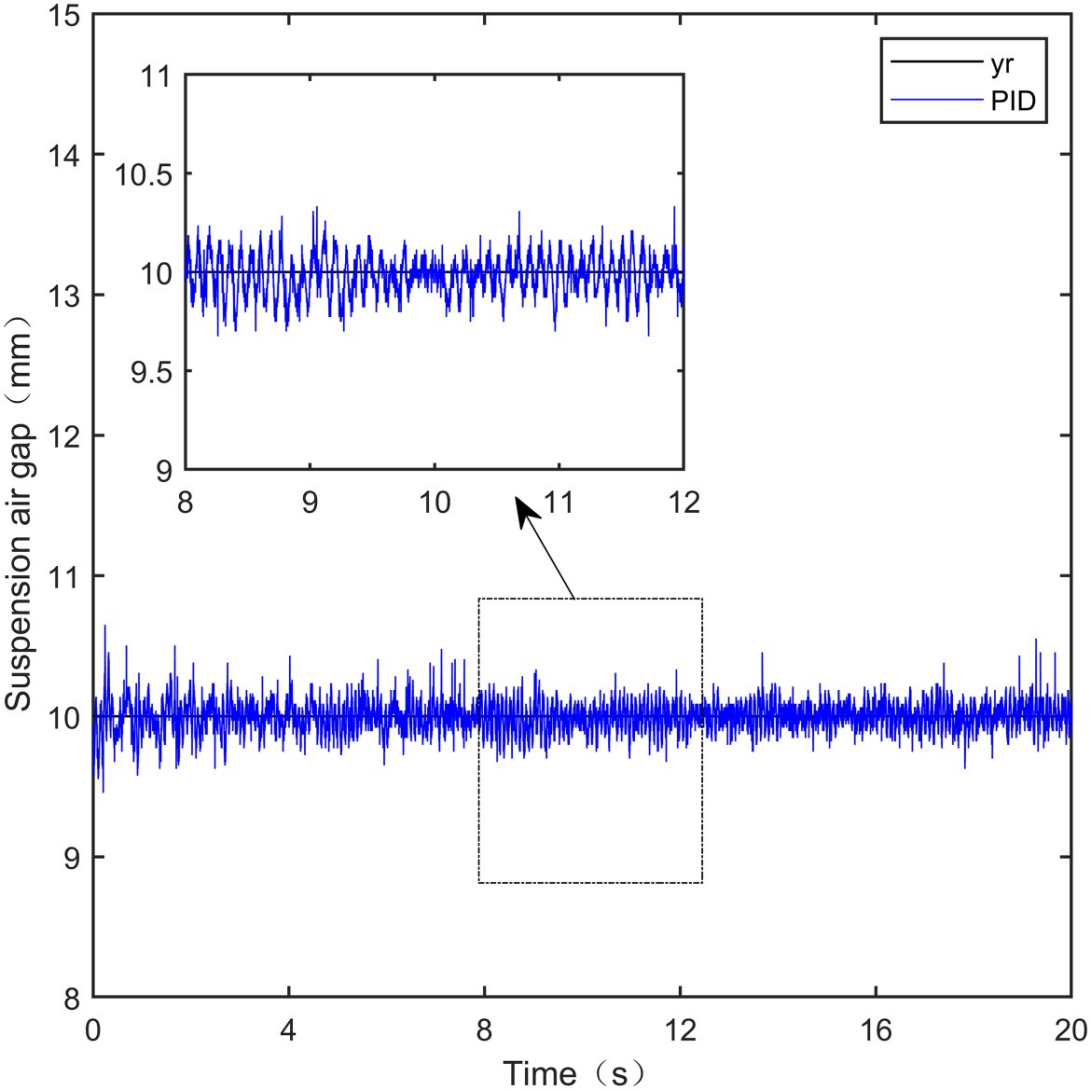
PID step signal experiment.

**Fig 22:**
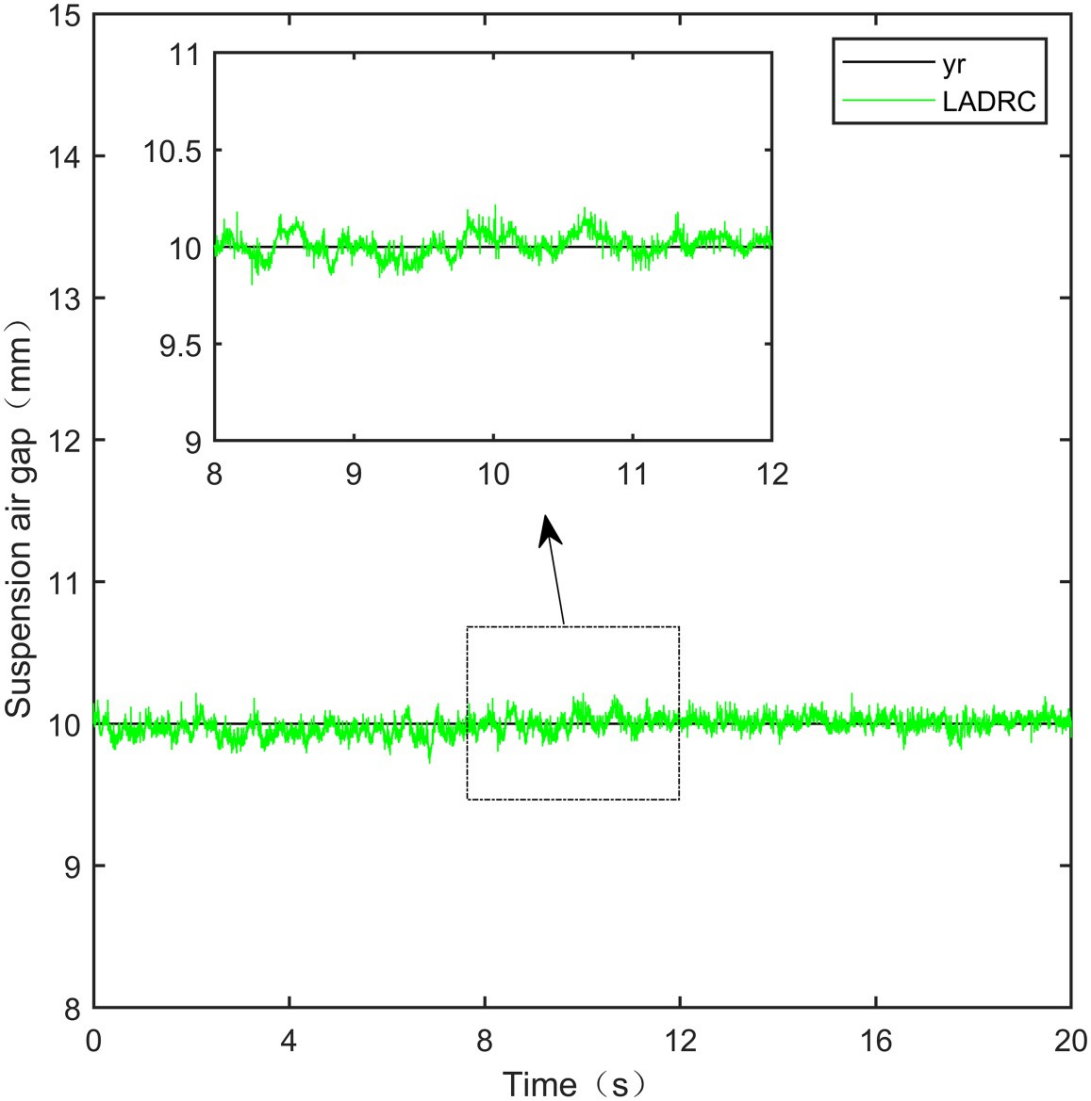
LADRC step signal experiment.

**Fig 23:**
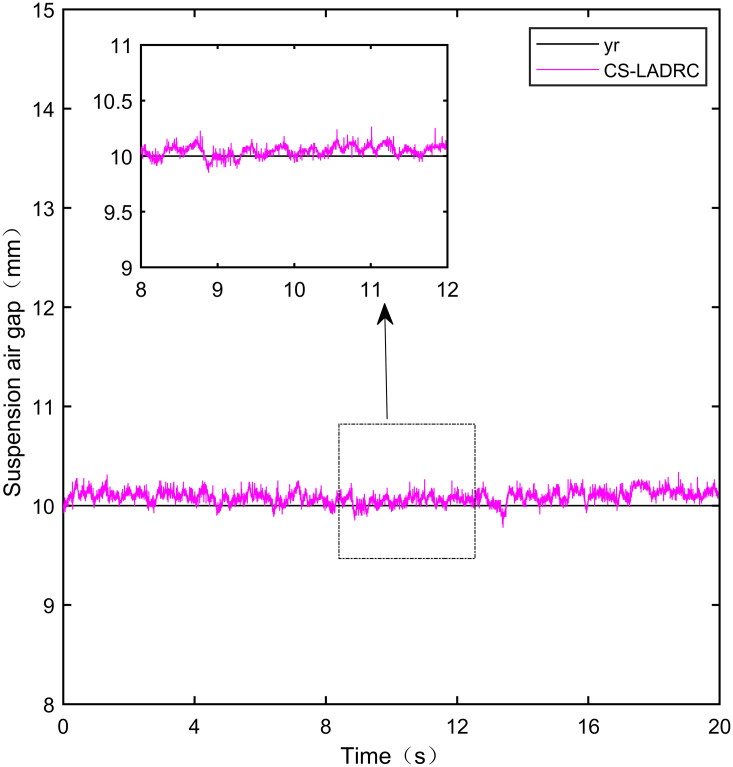
CS-LADRC step signal experiment.

**Fig 24:**
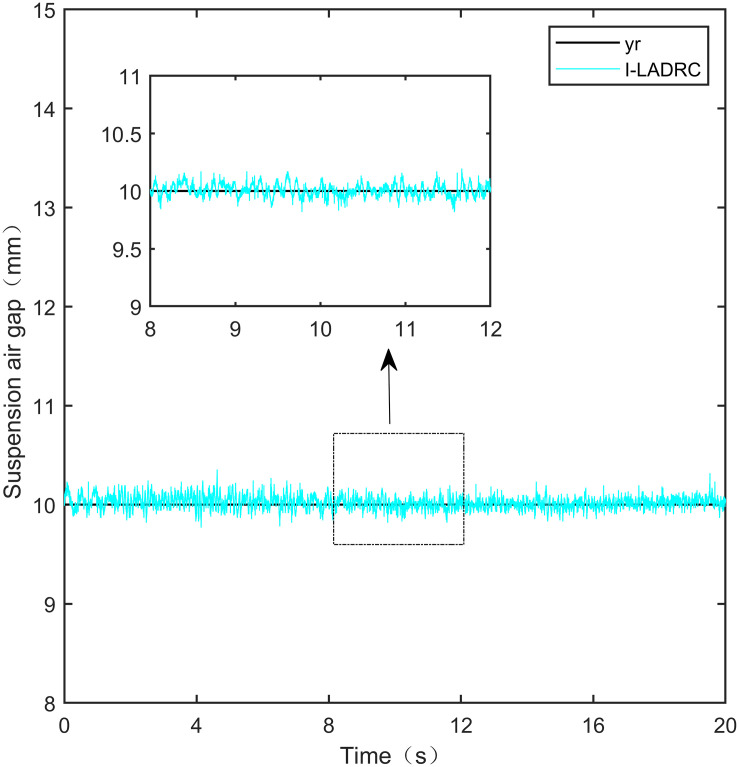
I-LADRC step signal experiment.

**Fig 25:**
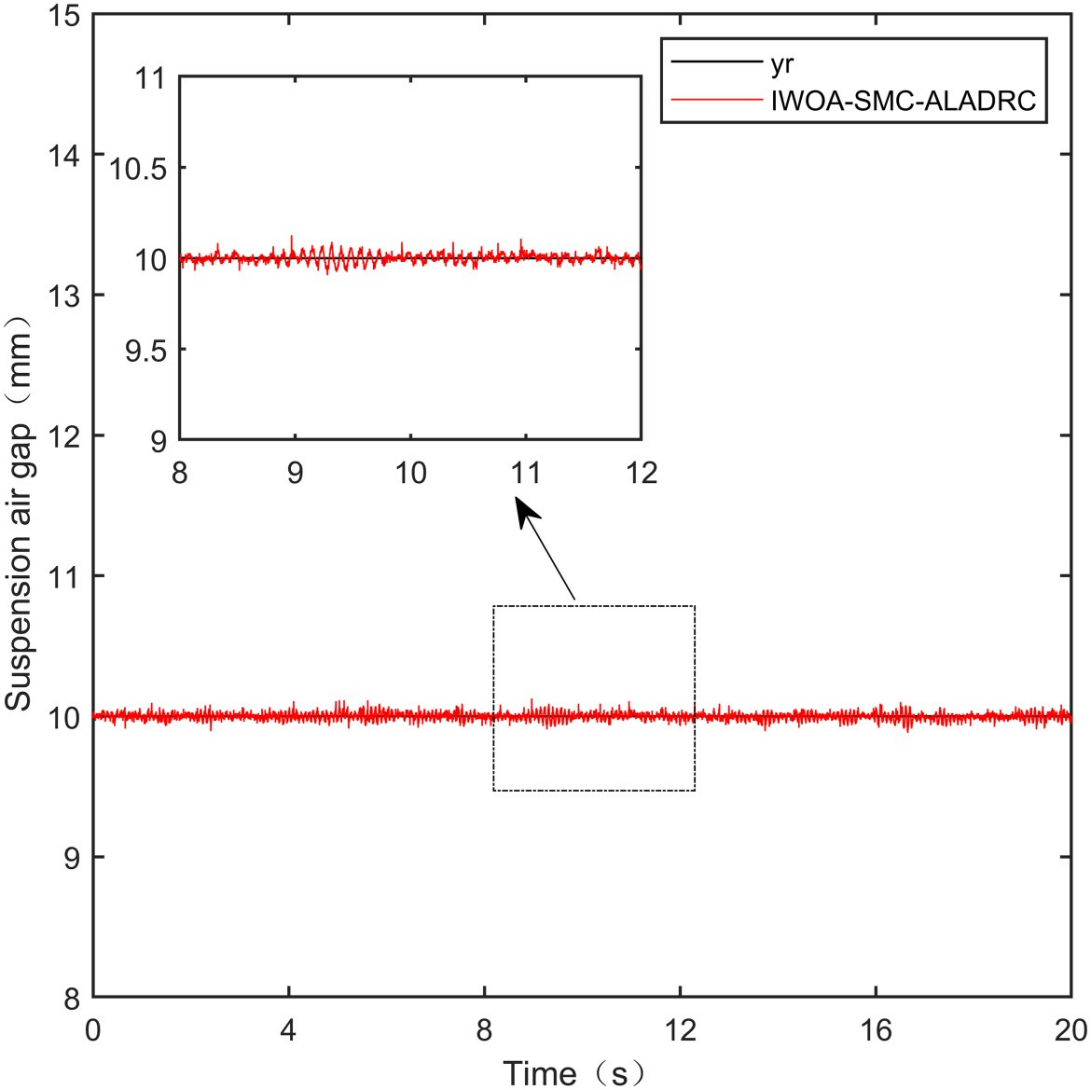
IWOA-SMC-ALADRC step signal experiment.

Here, we use *y*_*r*_ = 10*mm* as the standard suspension air gap to verify the control effect of the controller. The control effect is shown in Figs 21–25:

From Fig 21, it can be seen that the fluctuation range of the suspended air gap output by PID is ± 0.678mm, and high-frequency jitter occurs. From Figs 22–24, it can be seen that the fluctuation ranges of the suspended air gap output by LADRC, CS-LADRC, and I-LADRC are ± 0.311mm, ± 0.301mm, and ± 0.342mm, respectively. However, as shown in Fig 25, the fluctuation range of the suspended air gap output of IWOA SMC ALADRC is only ± 0.135mm. Compared with PID, the fluctuation range of the suspended air gap in IWOA-SMC-ALADRC has been reduced by 80.09%, and compared with LADRC, CS-LADRC, and I-LADRC, it has been reduced by 56.59%, 55.15%, and 60.53%, respectively. Therefore, the suspended air gap output of IWOA-SMC-ALADRC is more stable.

### 7.2 Anti-disturbance experiment

**Fig 26:**
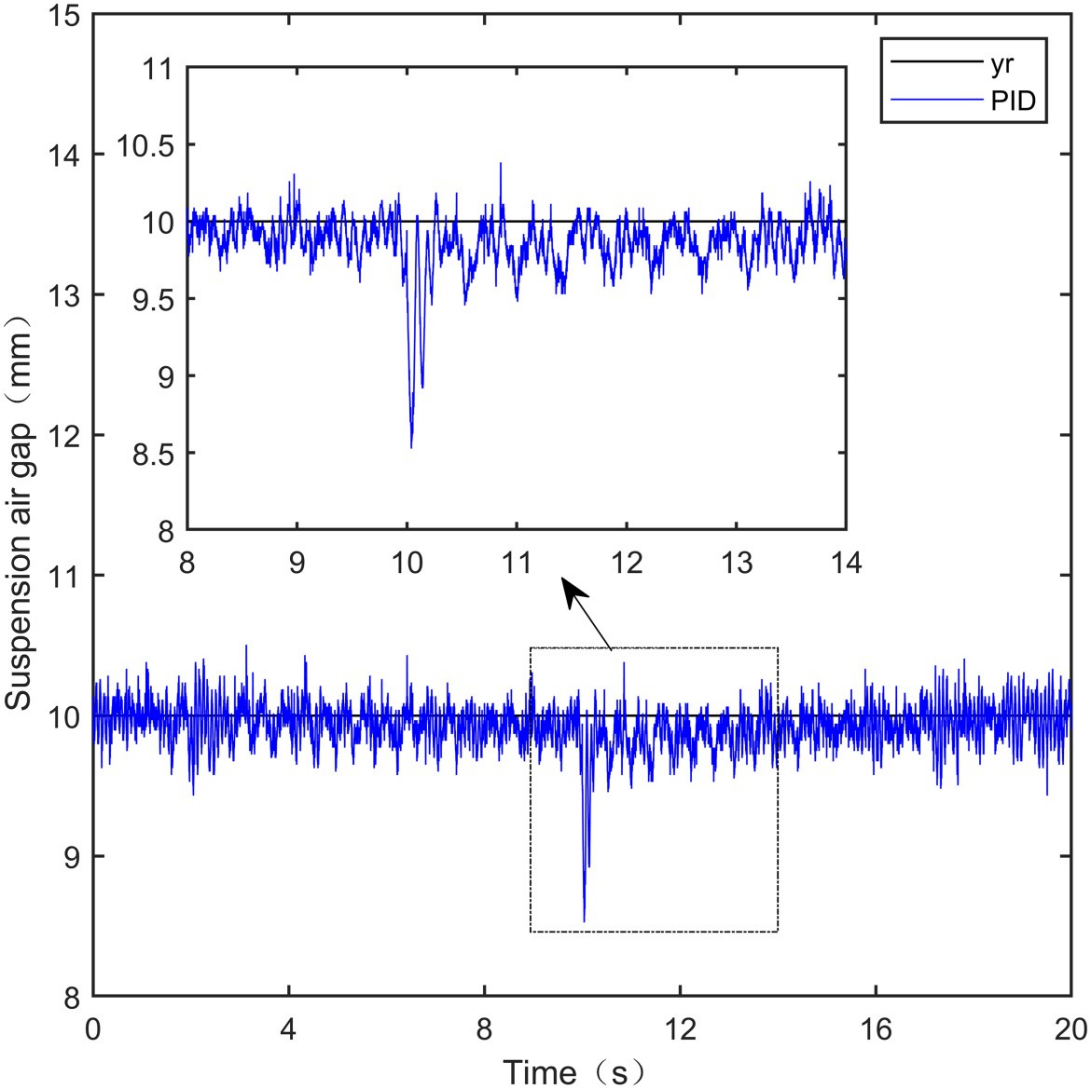
PID anti-disturbance experiment.

**Fig 27:**
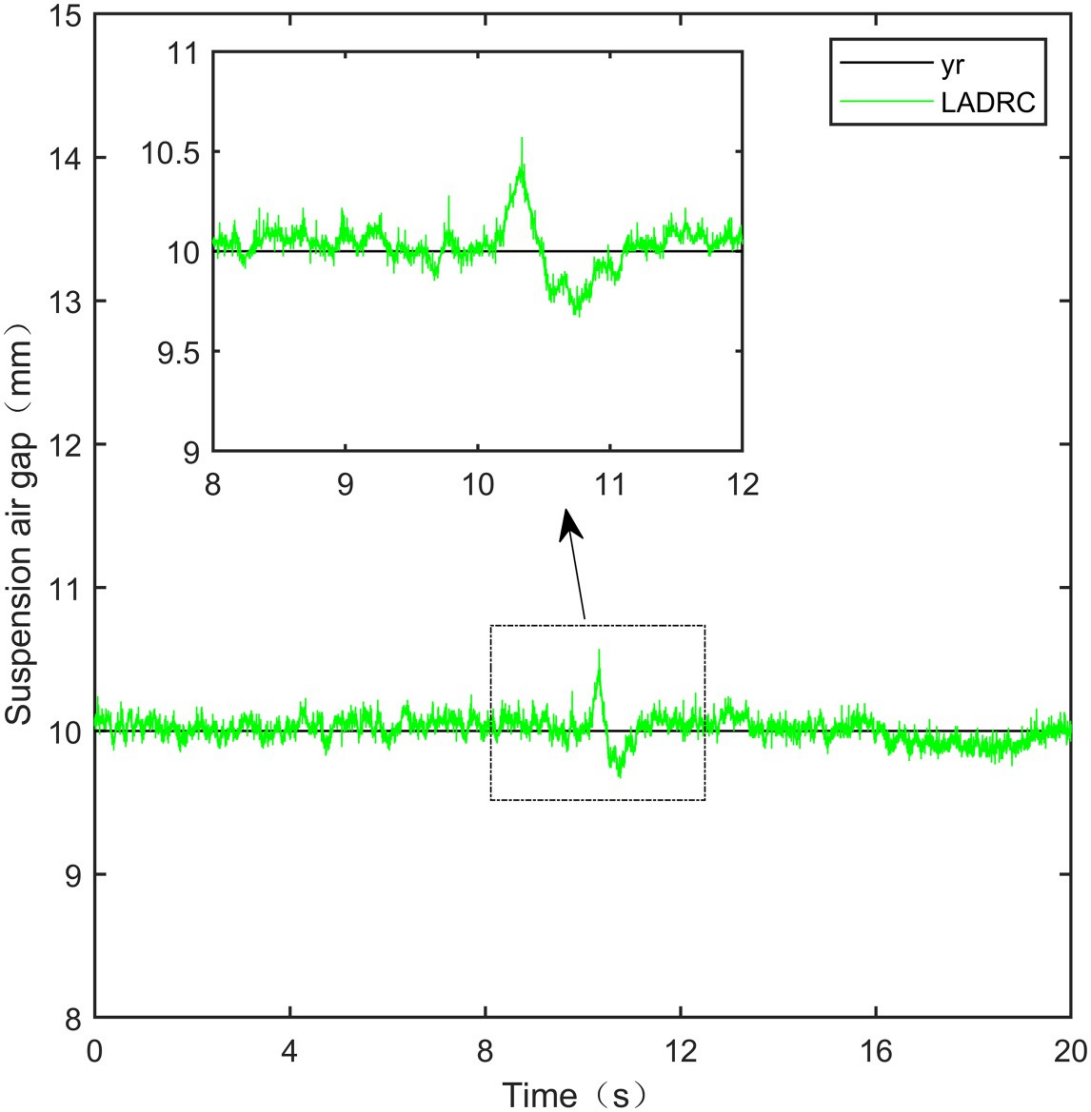
LADRC anti-disturbance experiment.

**Fig 28:**
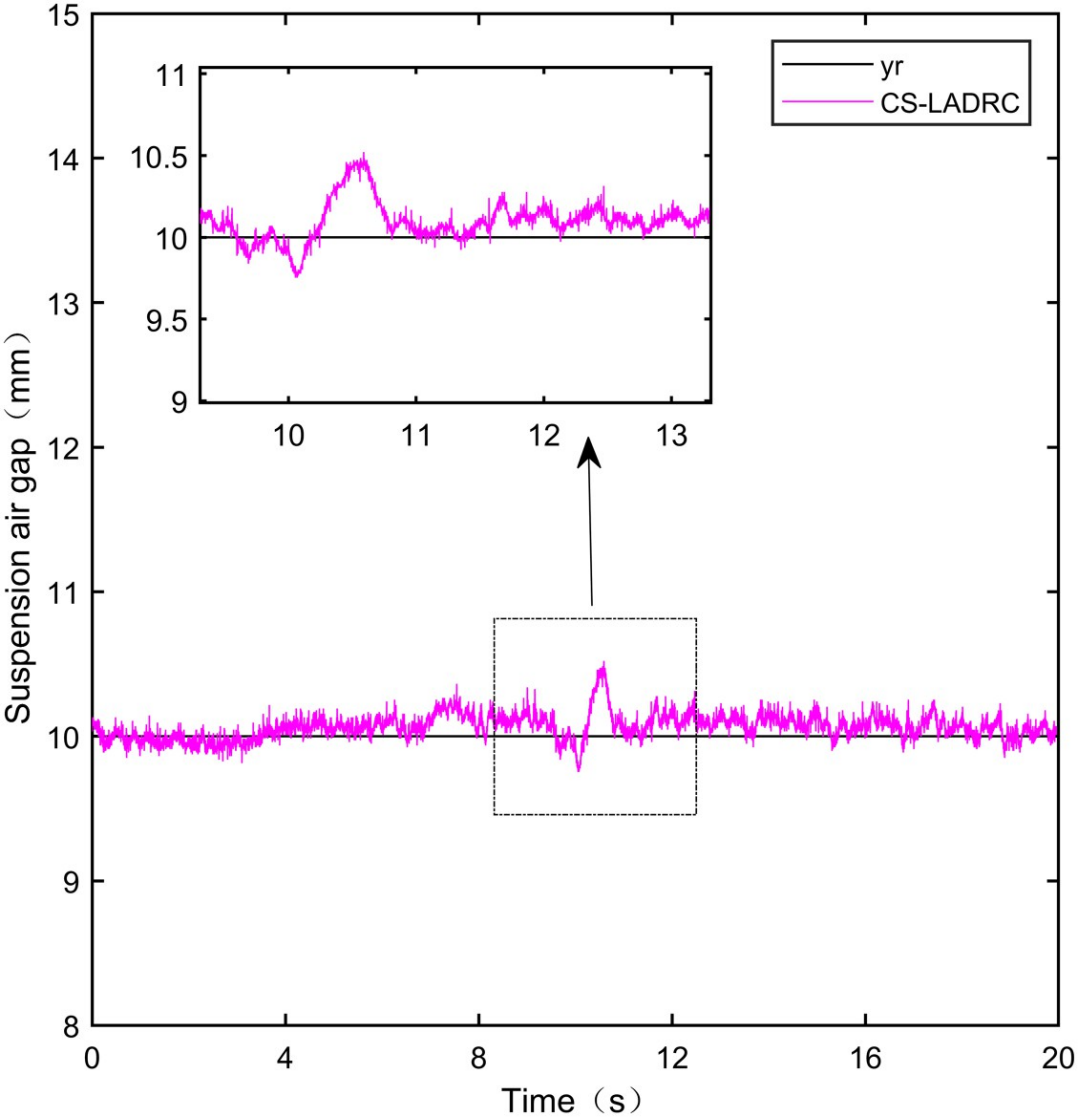
CS-LADRC anti-disturbance experiment.

**Fig 29:**
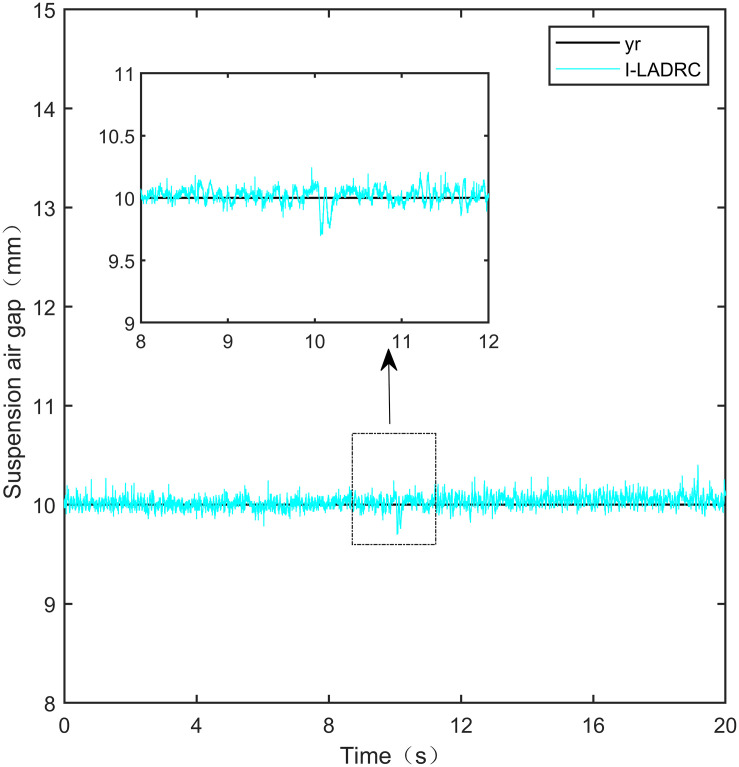
I-LADRC anti-disturbance experiment.

**Fig 30:**
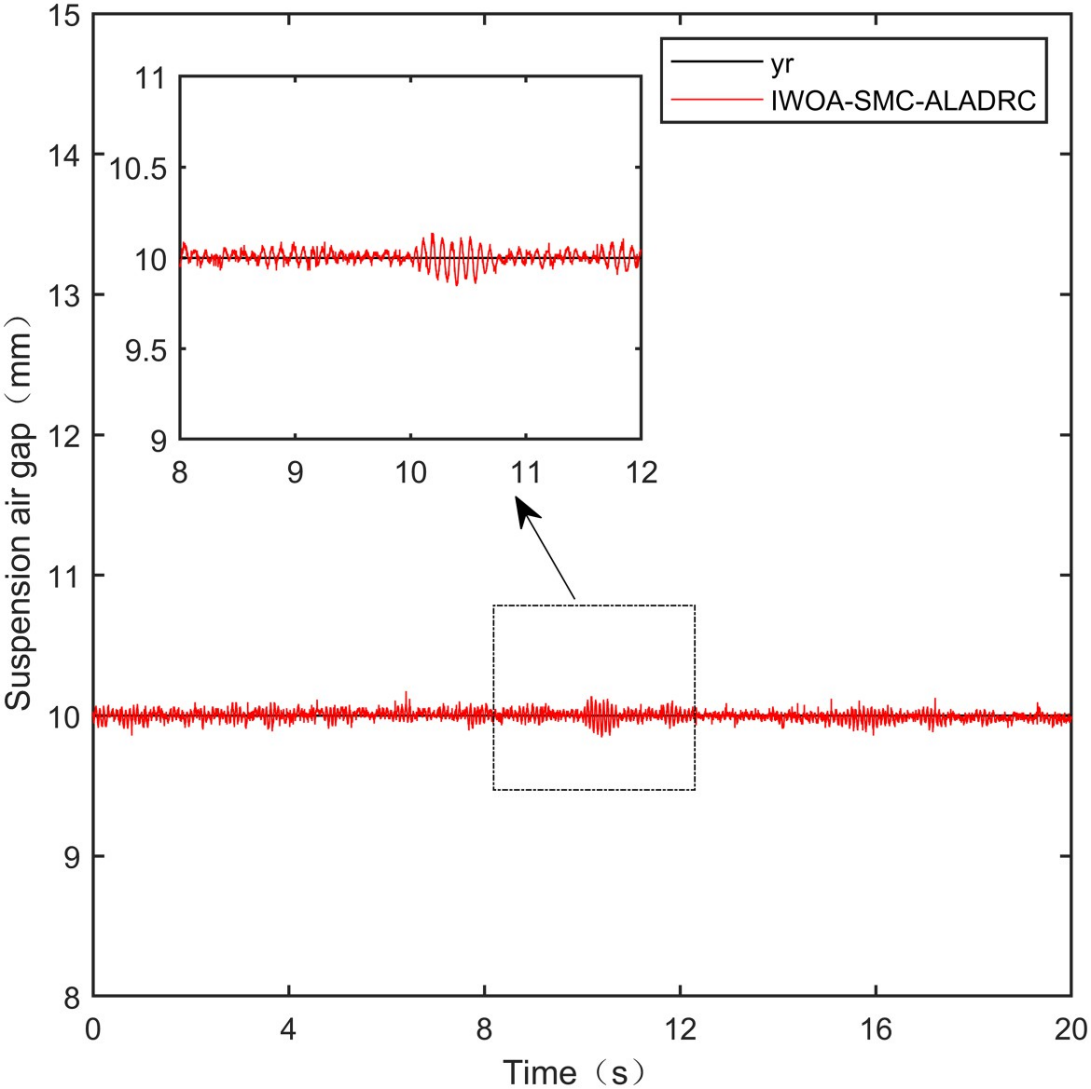
IWOA-SMC-ALADRC anti-disturbance experiment.

We applied disturbance to the system at the 10th second to analyze the anti-disturbance performance of the controller. The anti-disturbance effects of the controller are shown in Figs 26–30.

From Fig 26, it can be seen that after being disturbed, the suspended air gap output of the PID showed severe shaking, with a deviation distance of 1.501mm. From Figs 27–29, it can be seen that the deviation distances of LADRC, CS-LADRC, and LADRC are 0.510mm, 0.501mm, and 0.342mm, respectively. However, as shown in Fig 30, the deviation distance of IWOA-SMC-ALADARC is only 0.196mm. Compared with PID, the deviation distance of the suspended air gap output by IWOA-SMC-ALADRC decreased by 86.94% after disturbance. Compared with LADRC, CS-LADRC, and I-LADRC, it decreased by 62.67%, 60.88%, and 42.69%, respectively. Therefore, it can be seen that compared to the other four control methods, IWOA-SMC-ALADRC has better anti-disturbance performance.

### 7.3 Sine tracking experiment

**Fig 31:**
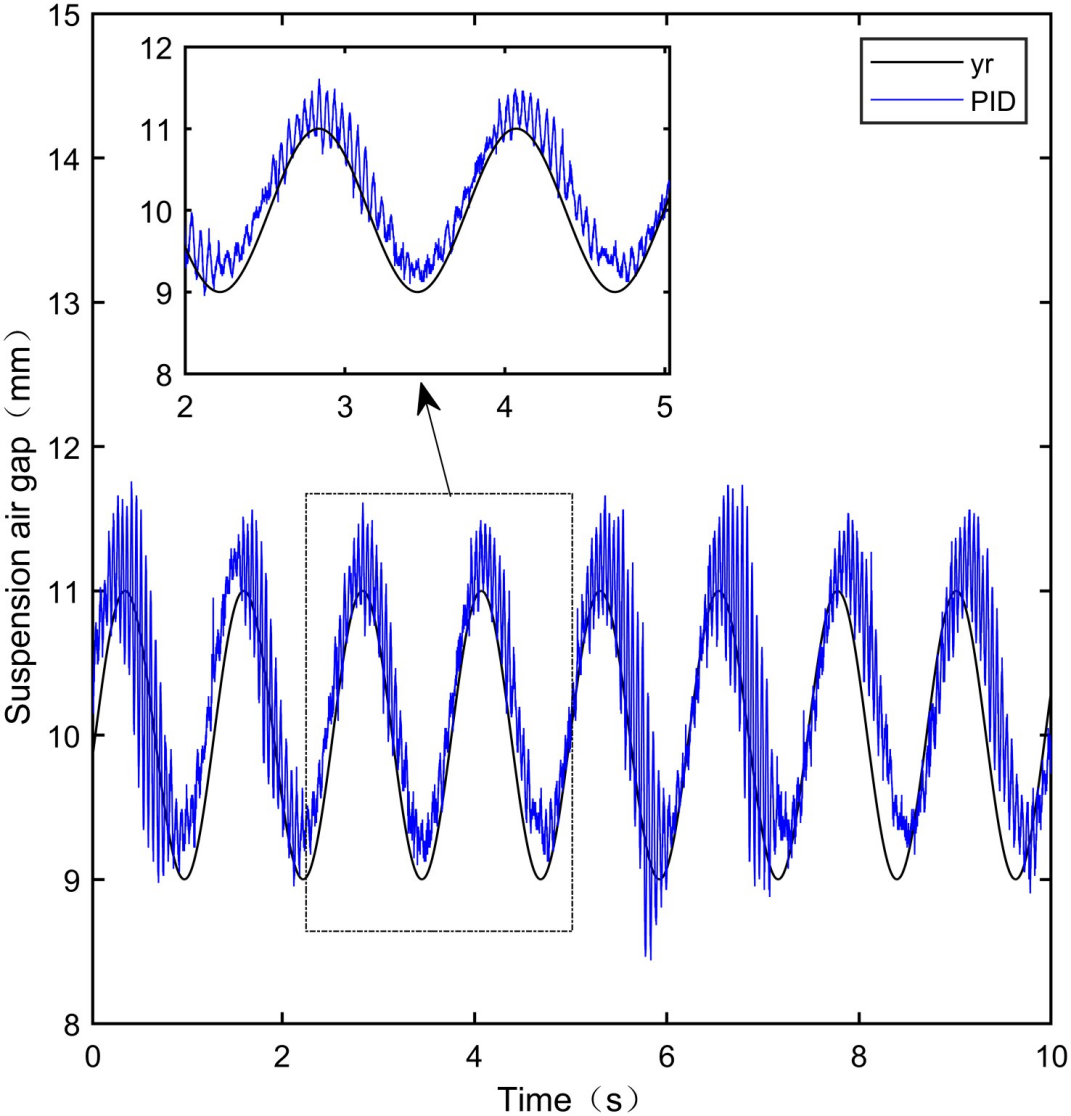
PID sine signal tracking experiment.

**Fig 32:**
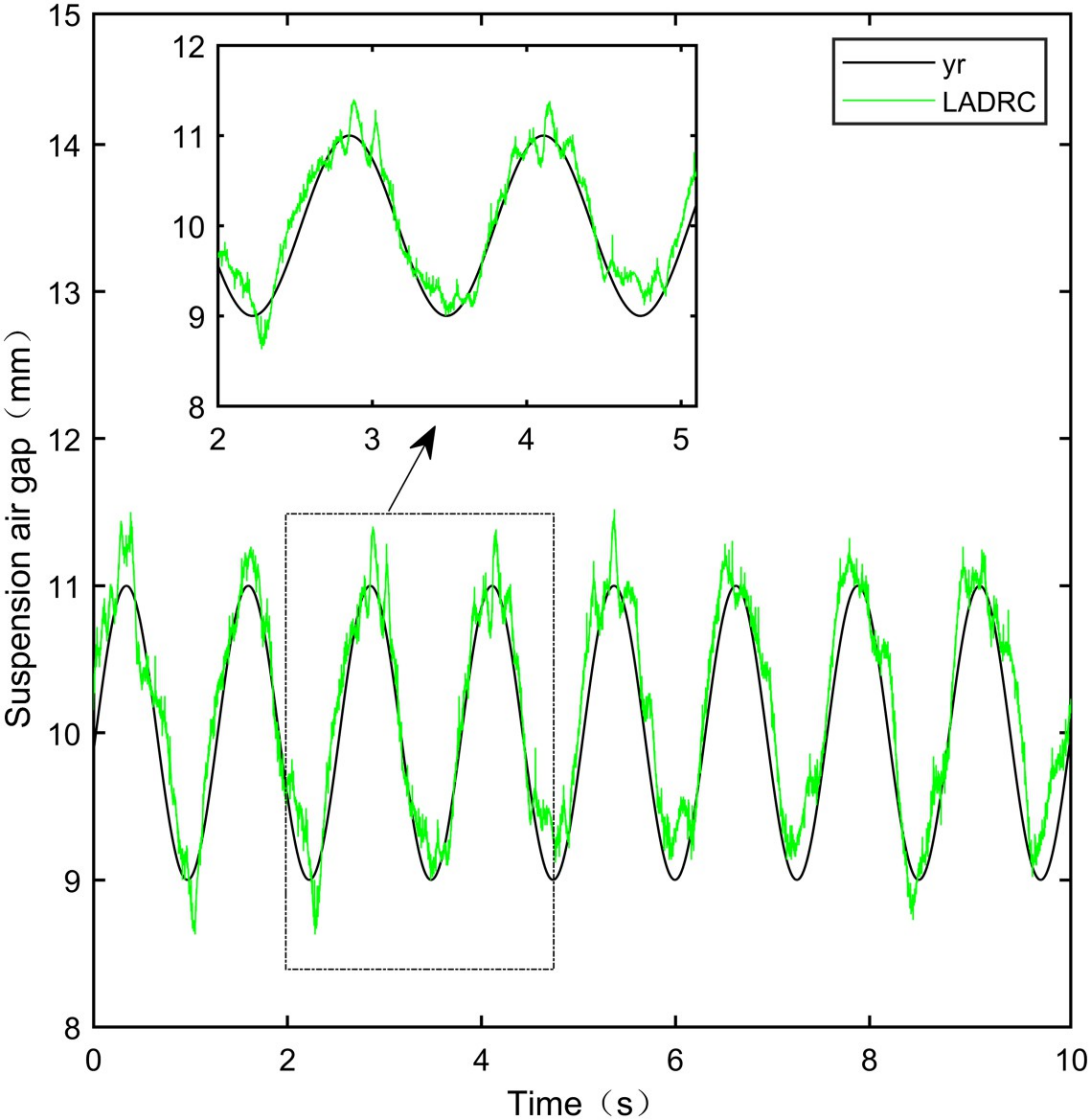
LADRC sine signal tracking experiment.

**Fig 33:**
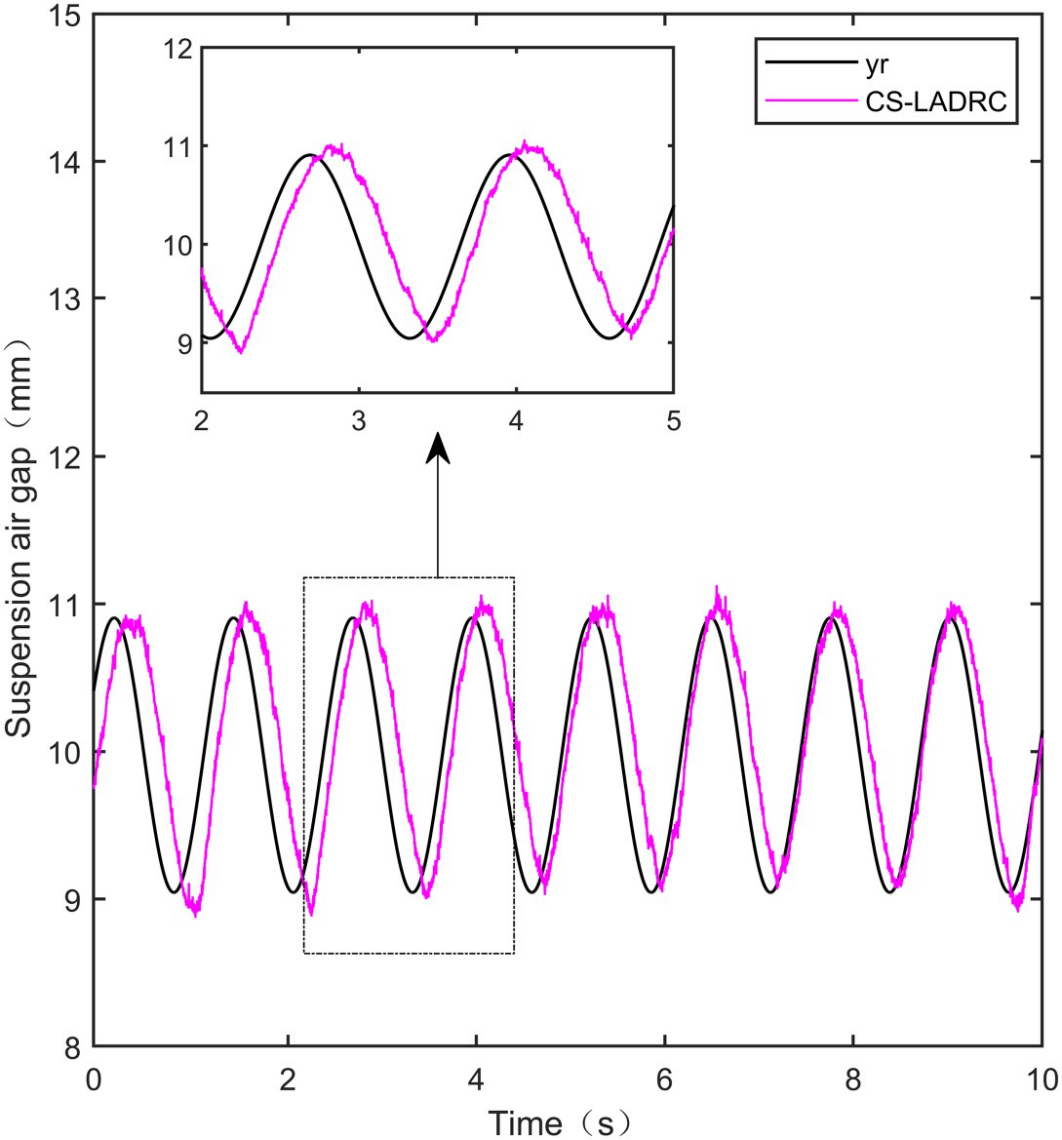
CS-LADRC sine signal tracking experiment.

**Fig 34:**
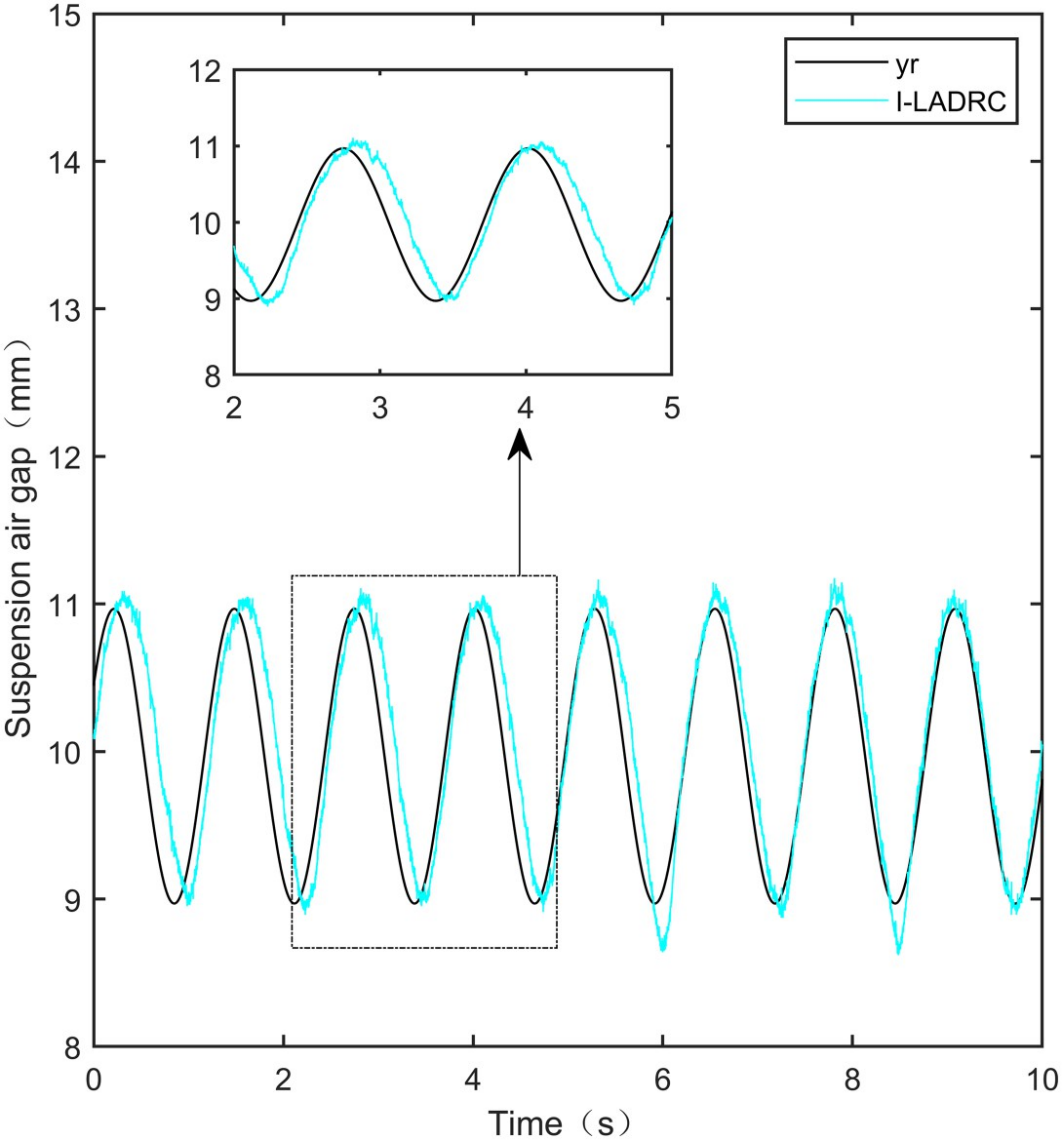
I-LADRC sine signal tracking experiment.

**Fig 35:**
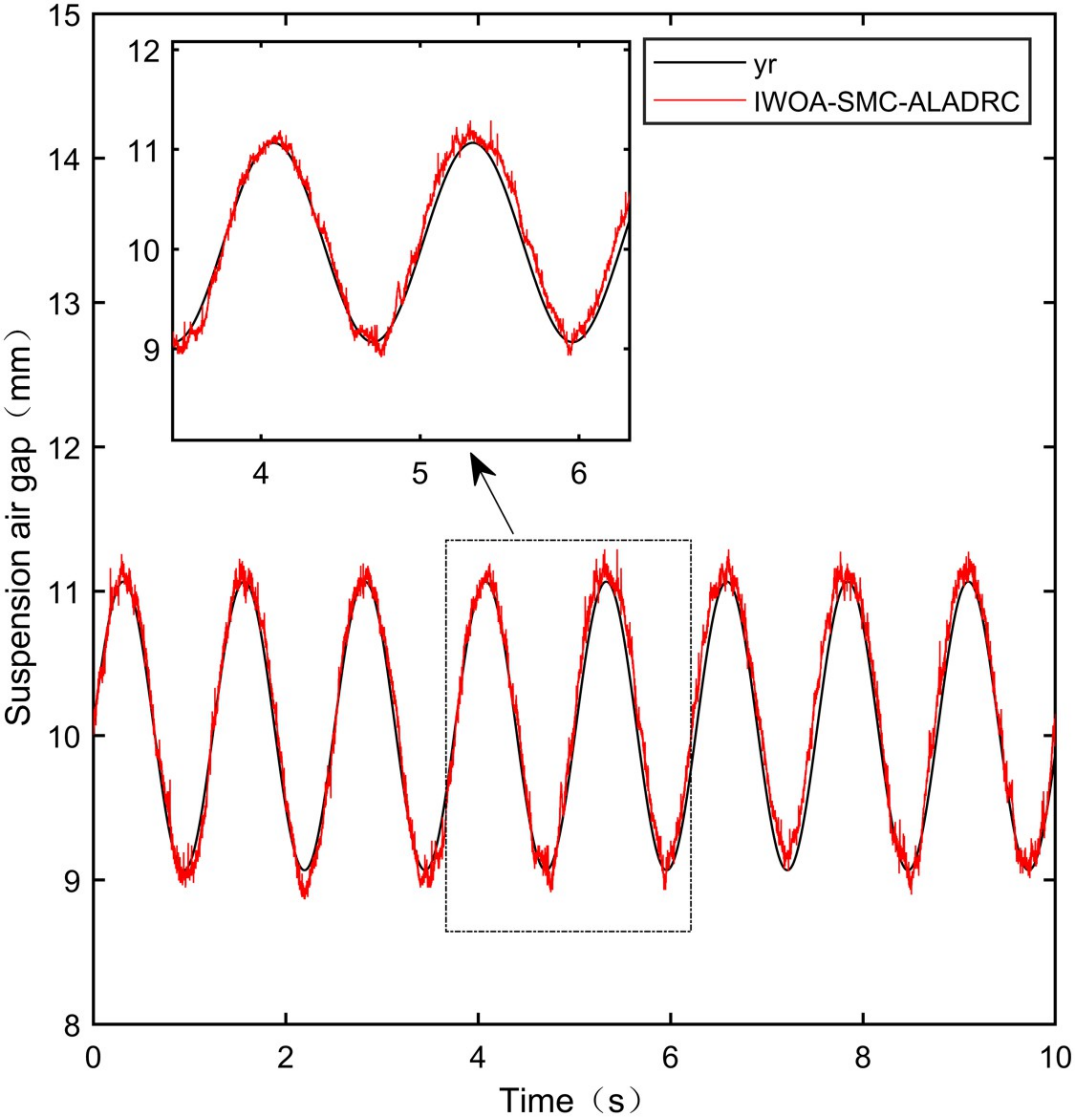
IWOA-SMC-ALADRC sine signal tracking experiment.

This experiment is a sine tracking experiment. Specifically, the goal is to analyze the tracking performance of the controller when the standard suspension air gap changes according to the sine signal law. The input sine signal is shown in Eq (47). The tracking effect is shown in Figs 31–35:

From Fig 31, it can be seen that there is high-frequency vibration in the suspended air gap output by the PID, with a fluctuation range of ± 0.744mm. From Figs 32–34, it can be seen that there is a delay phenomenon in the suspended air gap output by LADRC, CS-LADRC, and I-LADRC, and the fluctuation ranges are ± 0.421mm, ± 0.417mm, and ± 0.394mm, respectively. However, as shown in Fig 35, the suspended air gap of IWOA-SMC-ALADRC exhibits neither high-frequency vibration nor delay, and the fluctuation range of its output suspended air gap is only ± 0.245mm. Compared with PID, the fluctuation range of the suspended air gap output of IWOA-SMC-ALADRC has been reduced by 67.07%, and compared with LADRC, CS-LADRC, and I-LADRC, it has been reduced by 41.81%, 41.25%, and 37.82%, respectively. Therefore, the suspension gap output by IWOA-SMC-ALADRC can approach the standard suspension air gap to the maximum extent possible.

### 7.4 Sine tracking disturbance experiment

**Fig 36:**
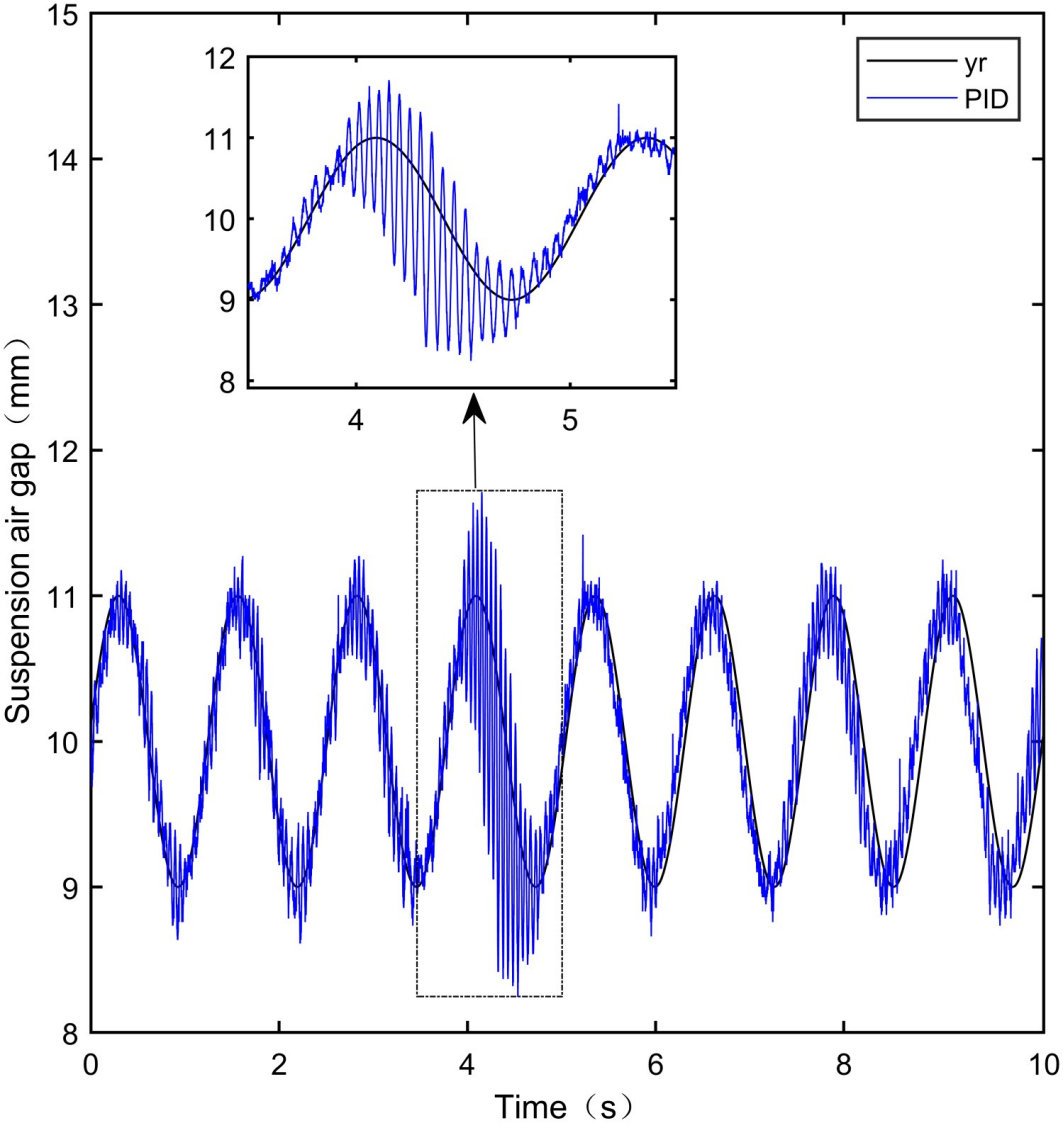
PID sine signal tracking disturbance experiment.

**Fig 37:**
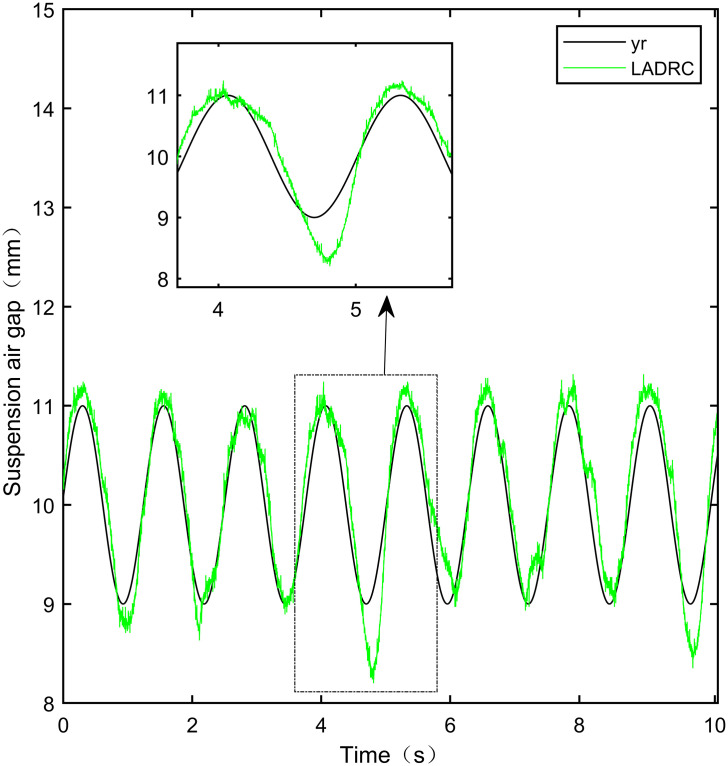
LADRC sine signal tracking disturbance experiment.

**Fig 38:**
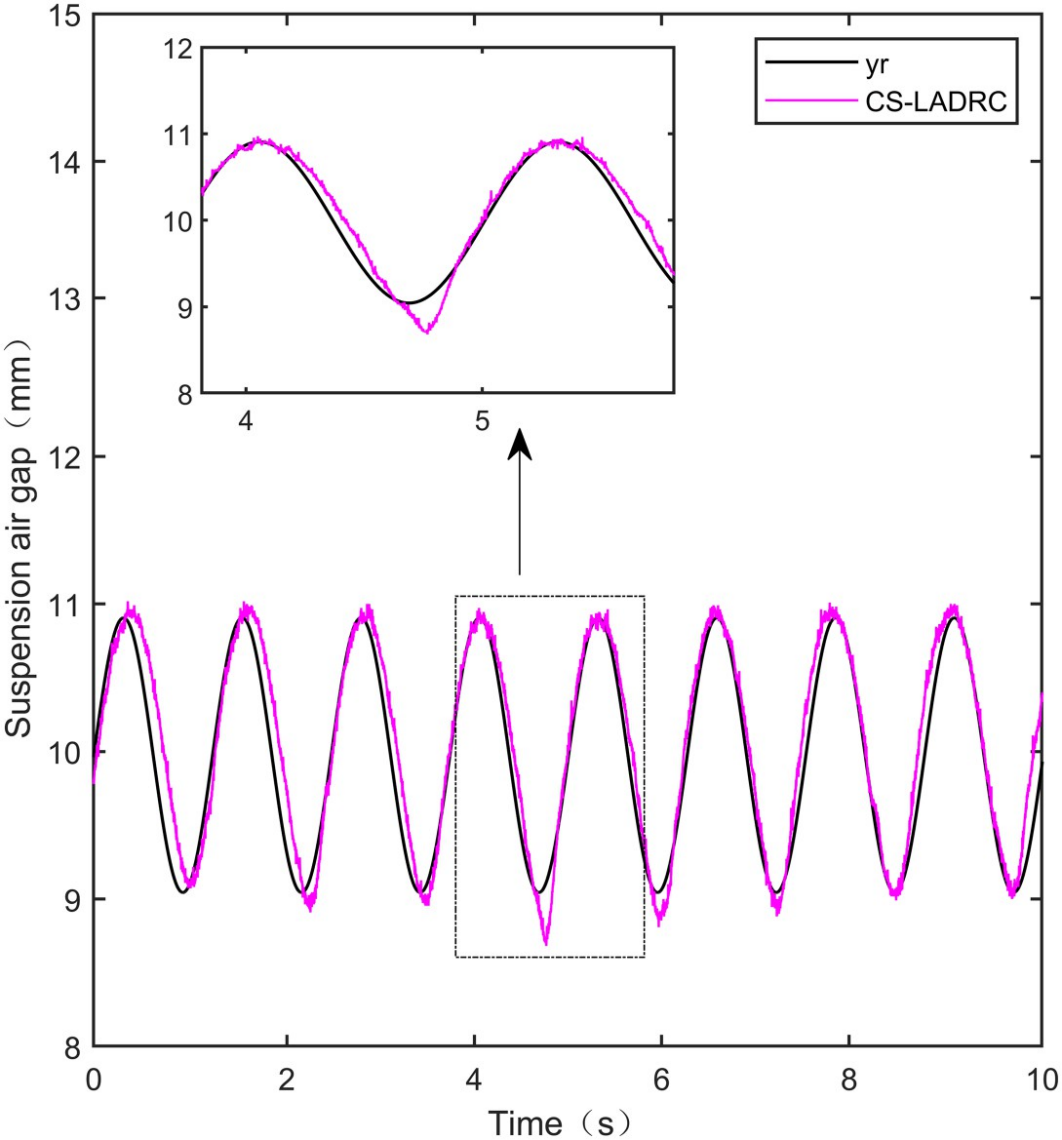
CS-LADRC sine signal tracking disturbance experiment.

**Fig 39:**
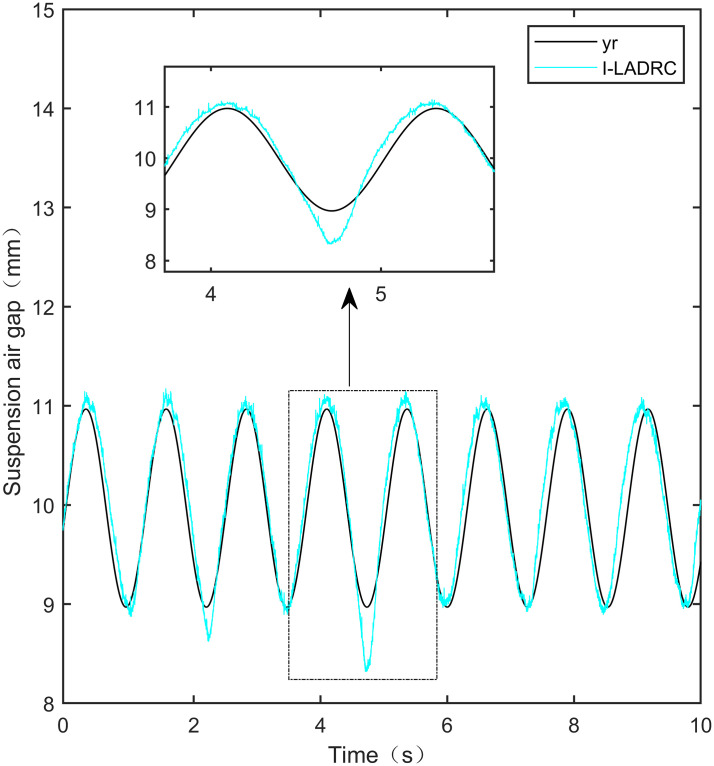
I-LADRC sine signal tracking disturbance experiment.

**Fig 40:**
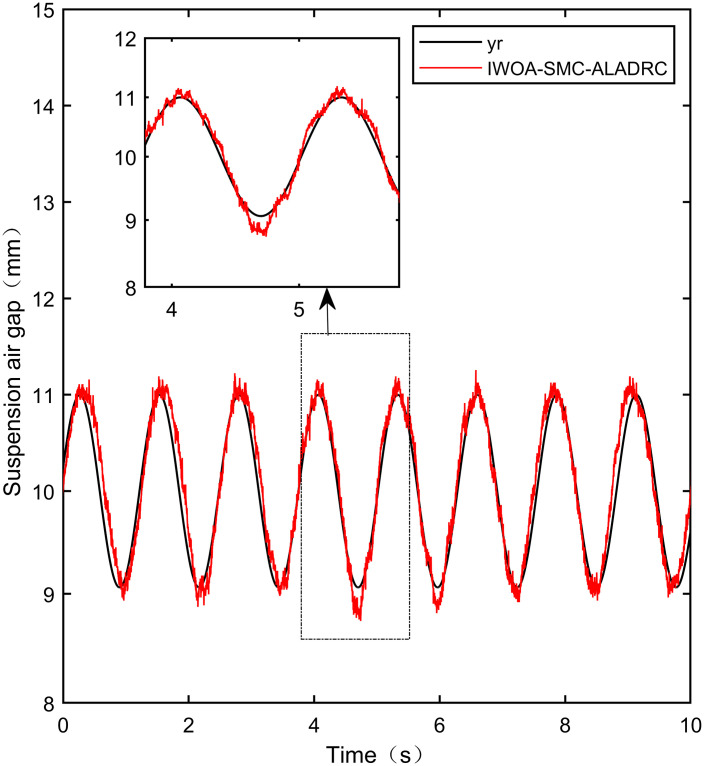
IWOA-SMC-ALADRC sine signal tracking disturbance experiment.

This experiment is a sine tracking interference experiment, apply disturbance to the system at the fifth second. Specifically, the goal is to analyse the anti-disturbance performance of the controller when the standard suspended air gap varies according to the sinusoidal signal law. The disturbance effect is shown in Figs 36–40:

From Figs 36–39, it can be seen that when the system is disturbed, the deviation distances between the PID, LADRC, CS-LADRC, and I-LADRC output suspension air gaps and the standard suspension air gap are 0.812mm, 0.743mm, 0.432mm, and 0.638mm, respectively. From Fig 40, it can be seen that the deviation distance of IWOA-SMC-ALADRC is only 0.322mm, compared with PID, LADRC, CS-LADRC, and I-LADRC, the deviation distance of IWOA-SMC-ALADRC decreased by 60.34%, 56.66%, 25.46%, and 49.53%, respectively. It can be seen that when the standard air gap follows a sine signal change, the anti-disturbance performance of IWOA-SMC-ALADRC is stronger than that of the other four control methods.

### 7.5 Square wave tracking experiment

**Fig 41:**
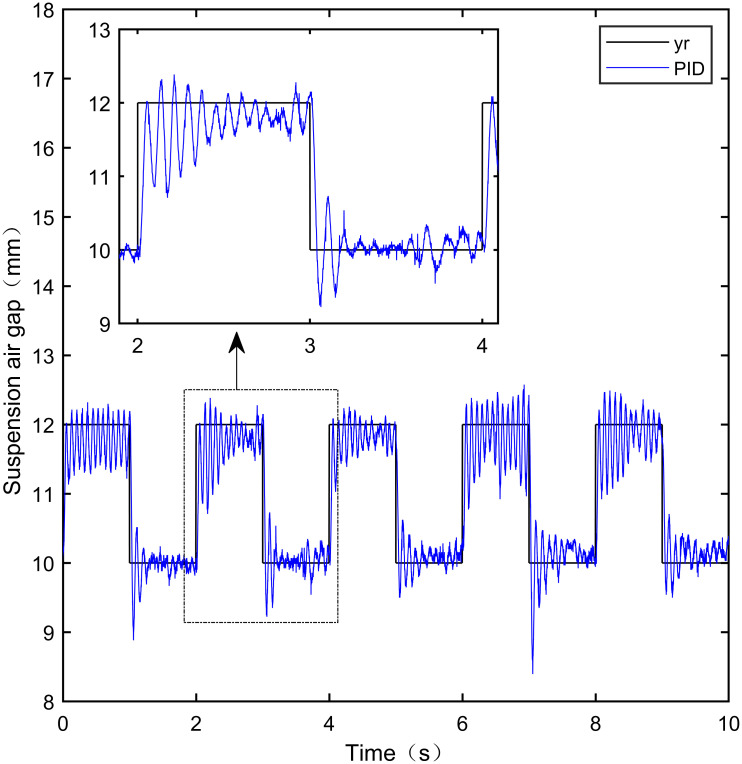
PID square wave tracking experiment.

**Fig 42:**
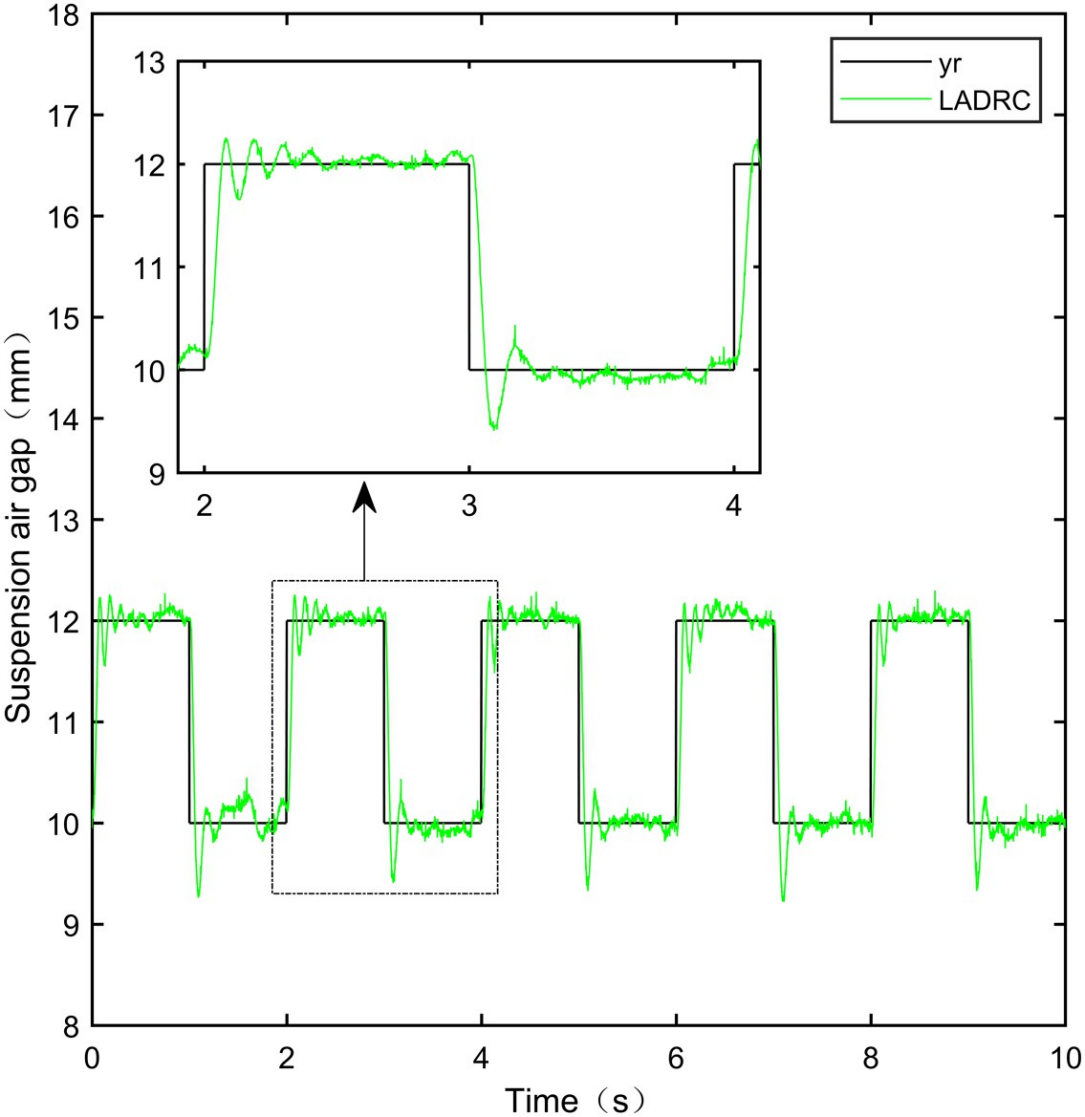
LADRC square wave tracking experiment.

**Fig 43:**
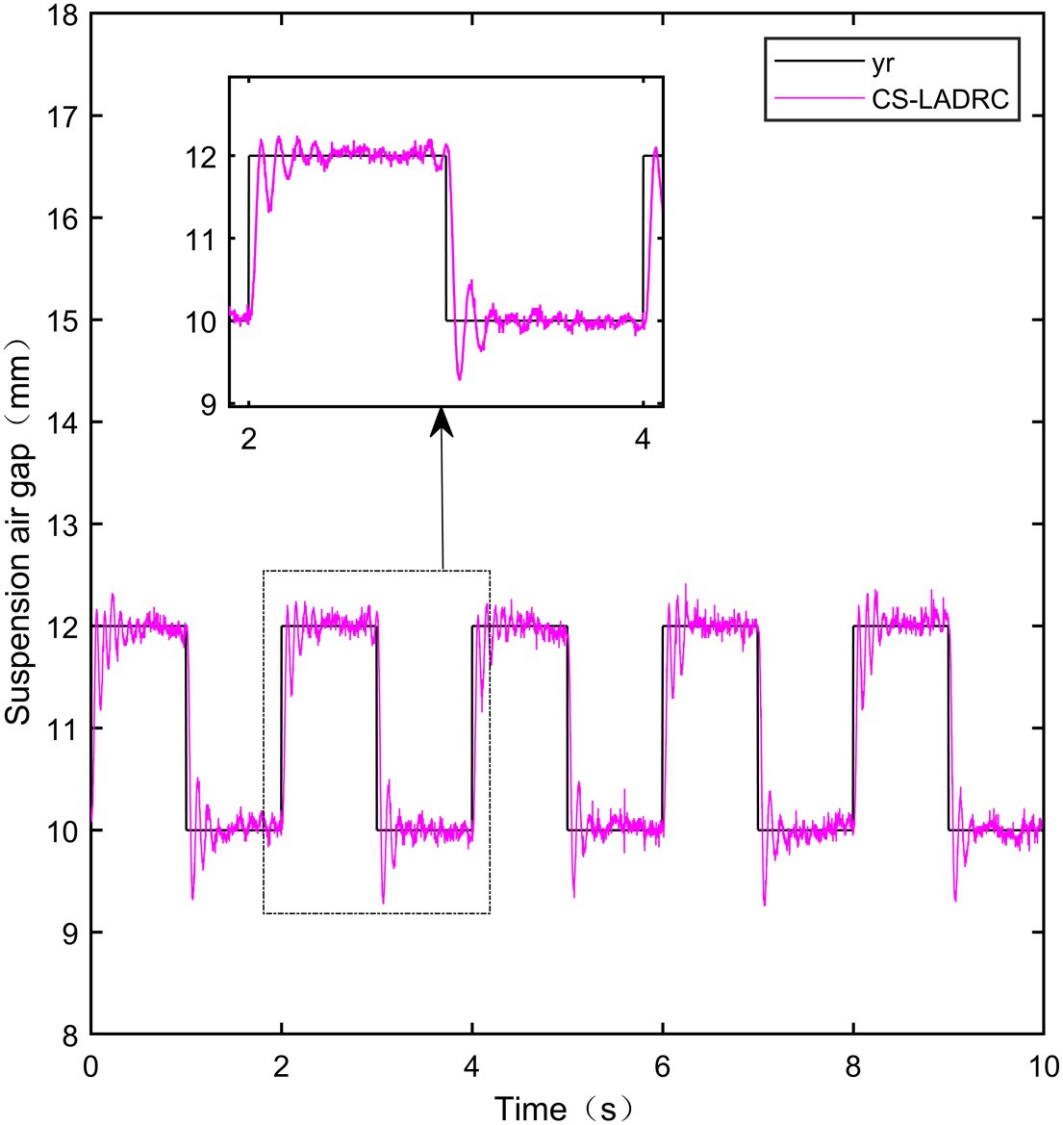
CS-LADRC square wave tracking experiment.

**Fig 44:**
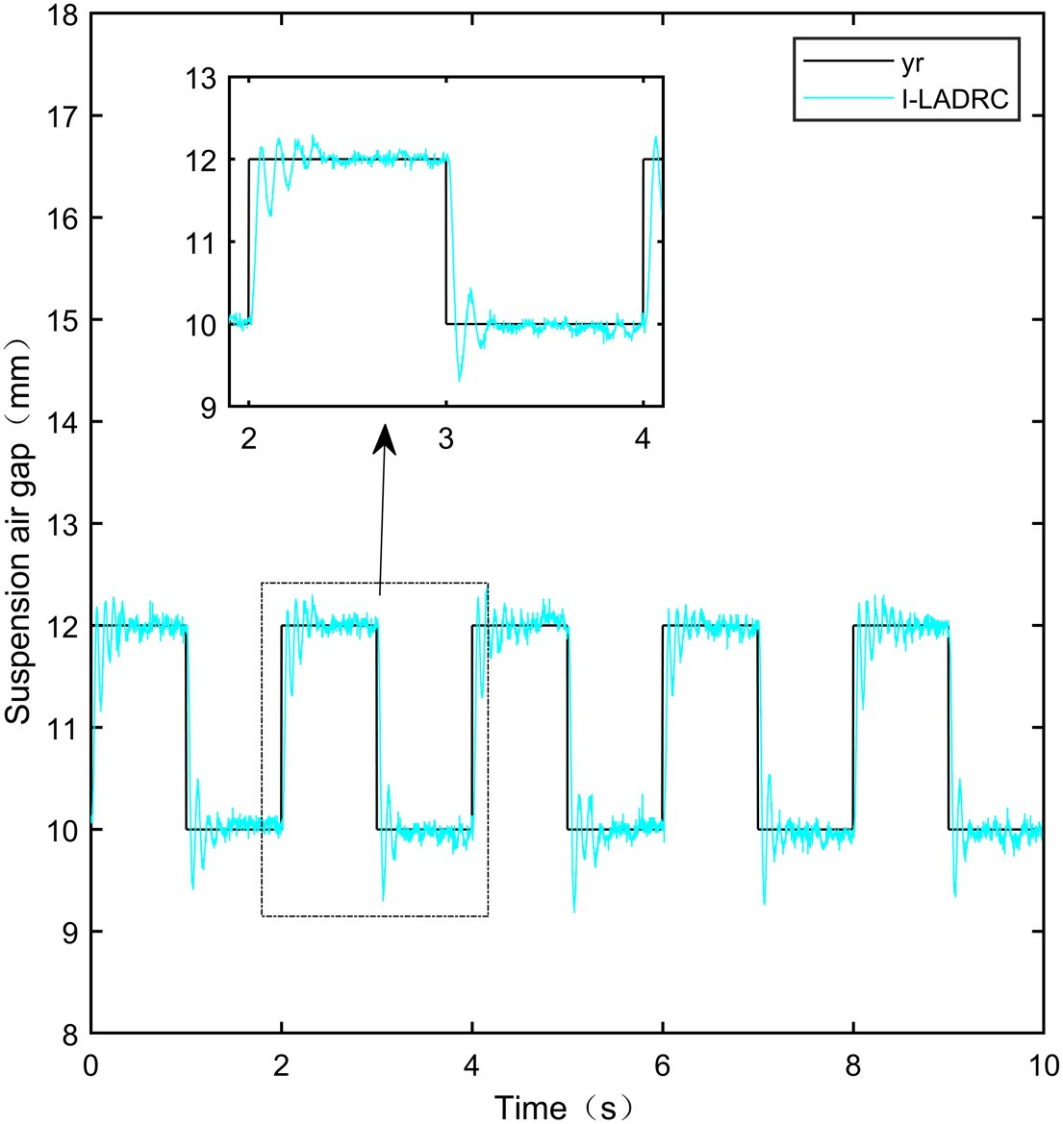
I-LADRC square wave tracking experiment.

**Fig 45:**
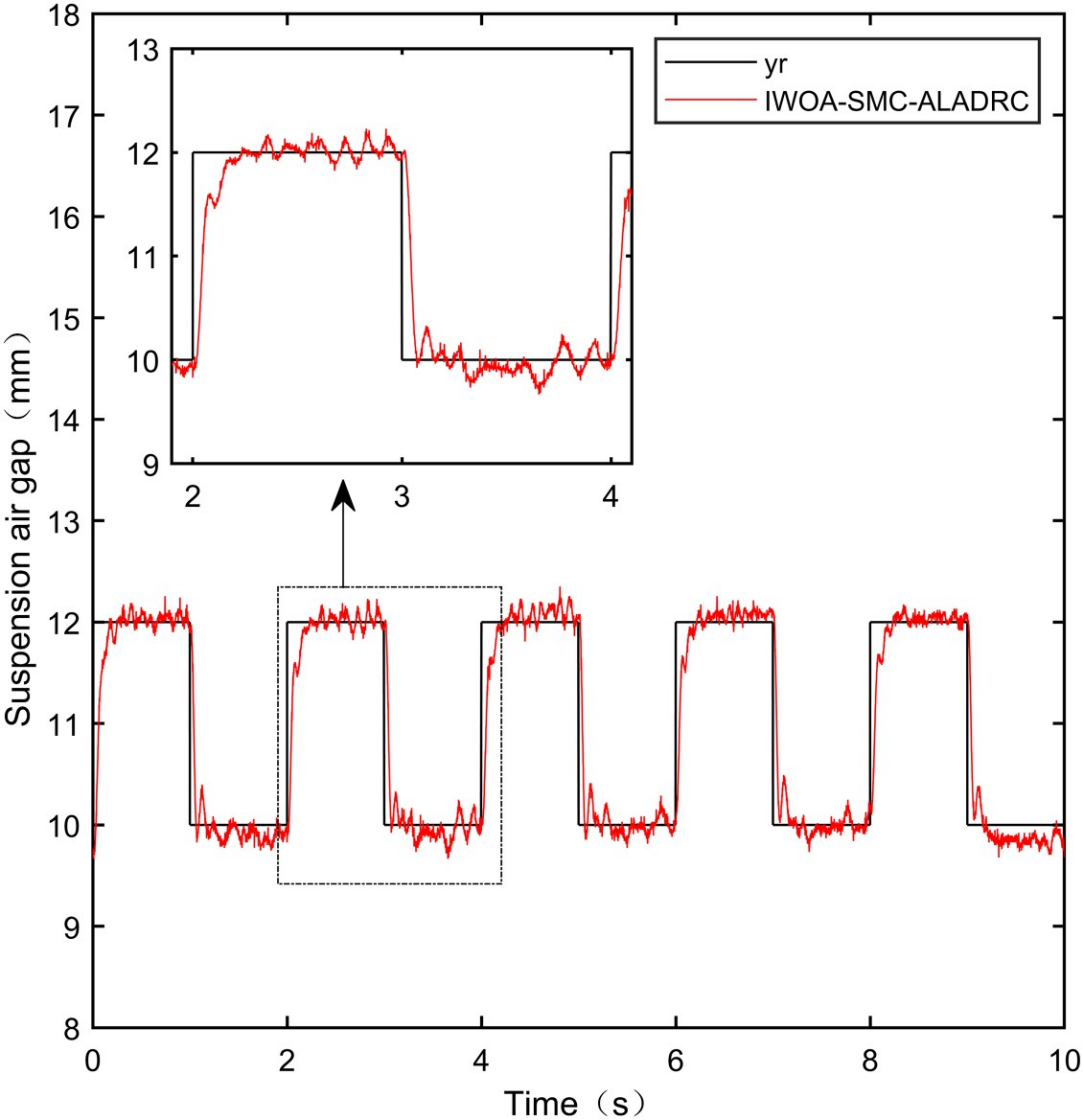
IWOA-SMC-ALADRC square wave tracking experiment.

This experiment is an analysis of square wave tracking performance. Specifically, the goal is to analyze the tracking performance of the controller when the standard suspension air gap changes according to the rules of the square wave signal. The mathematical expression for the square wave signal input into the system is shown in Eq (48). The tracking effect is shown in Figs 41–45.

From Fig 41, it can be seen that the PID output exhibits high-frequency vibration in the suspended air gap, with a fluctuation range of up to ± 1.762mm. From Figs 42–44, it can be seen that the fluctuation ranges of the suspended air gap output by LADRC, CS-LADRC, and I-LADRC are ± 0.792mm, ± 0.801mm, and ± 0.799mm, respectively. From Fig 45, it can be seen that there is no high-frequency vibration in the suspended air gap output by IWOA-SMC-ALADRC, and the fluctuation range of its output suspended air gap is only ± 0.312mm. Compared with PID, the fluctuation range of the suspended air gap output by IWOA-SMC-ALADRC has been reduced by 82.29%. Compared with LADRC, CS-LADRC, and I-LADRC, the fluctuation range has been reduced by 60.60%, 61.08%, and 60.95%, respectively. Therefore, the suspended air gap output by IWOA-SMC-ALADRC can approach the standard suspended air gap to the maximum extent possible.

### 7.6 Square wave tracking disturbance experiment

**Fig 46:**
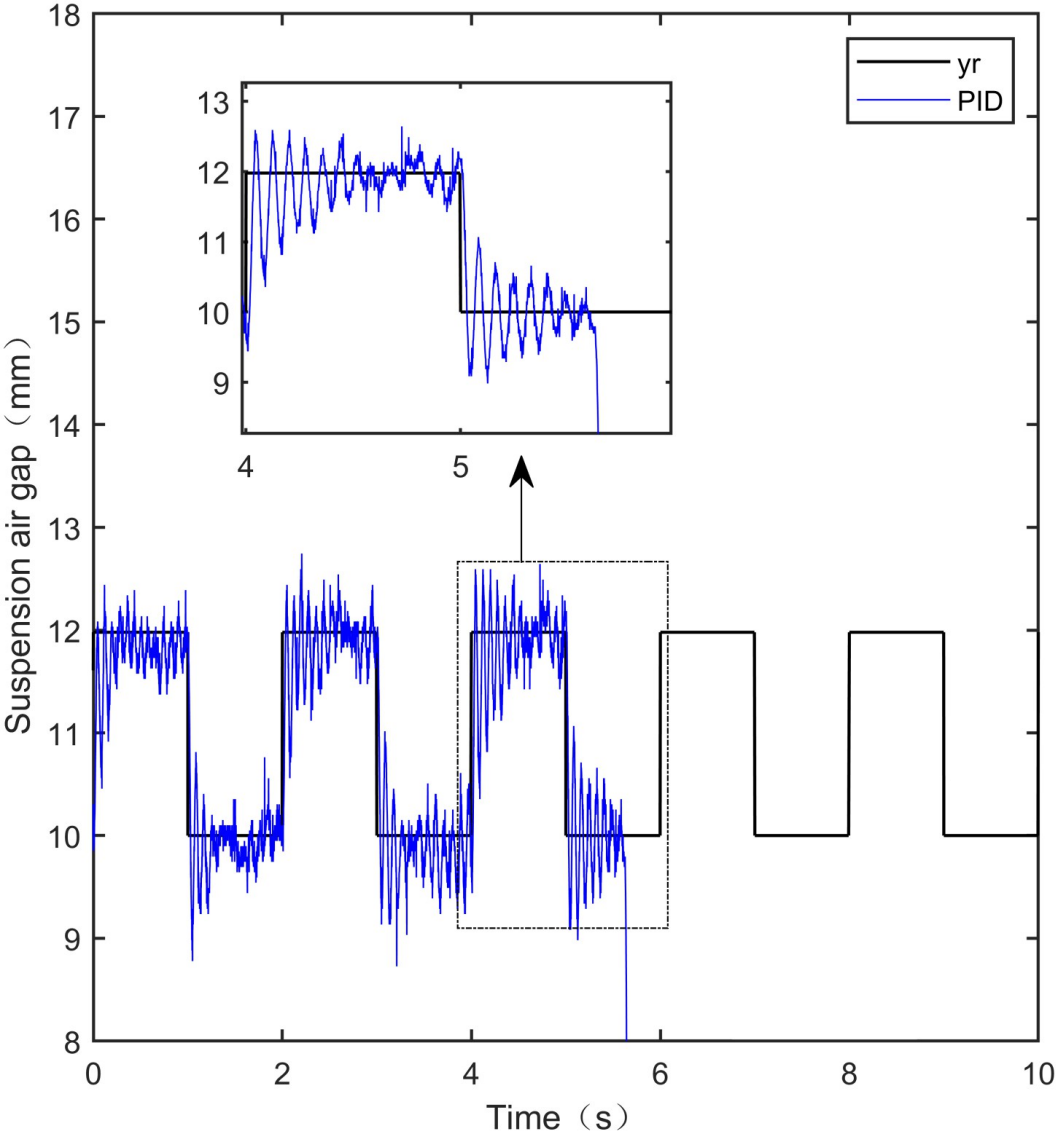
PID square wave tracking disturbance experiment.

**Fig 47:**
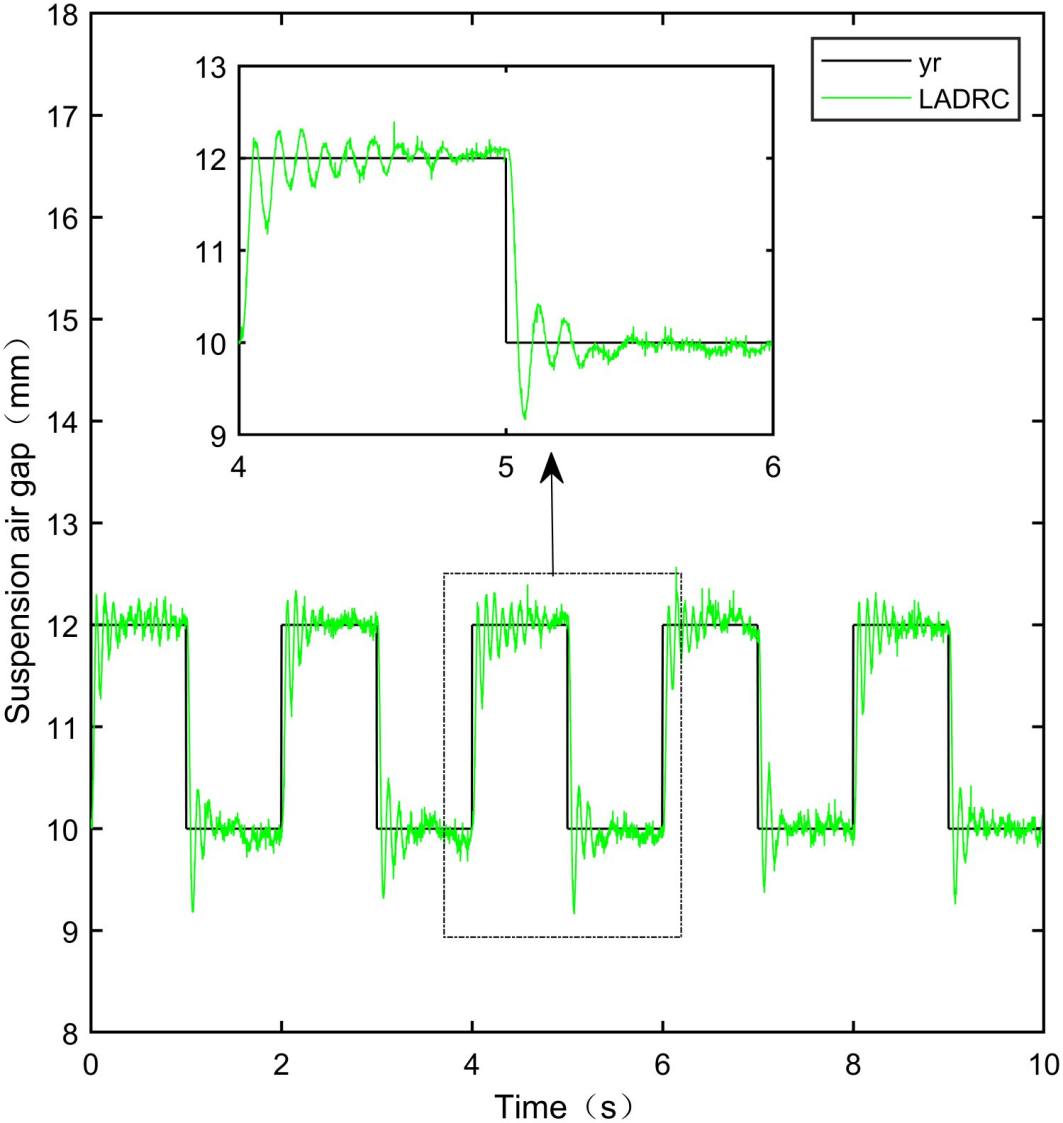
LADRC square wave tracking disturbance experiment.

**Fig 48:**
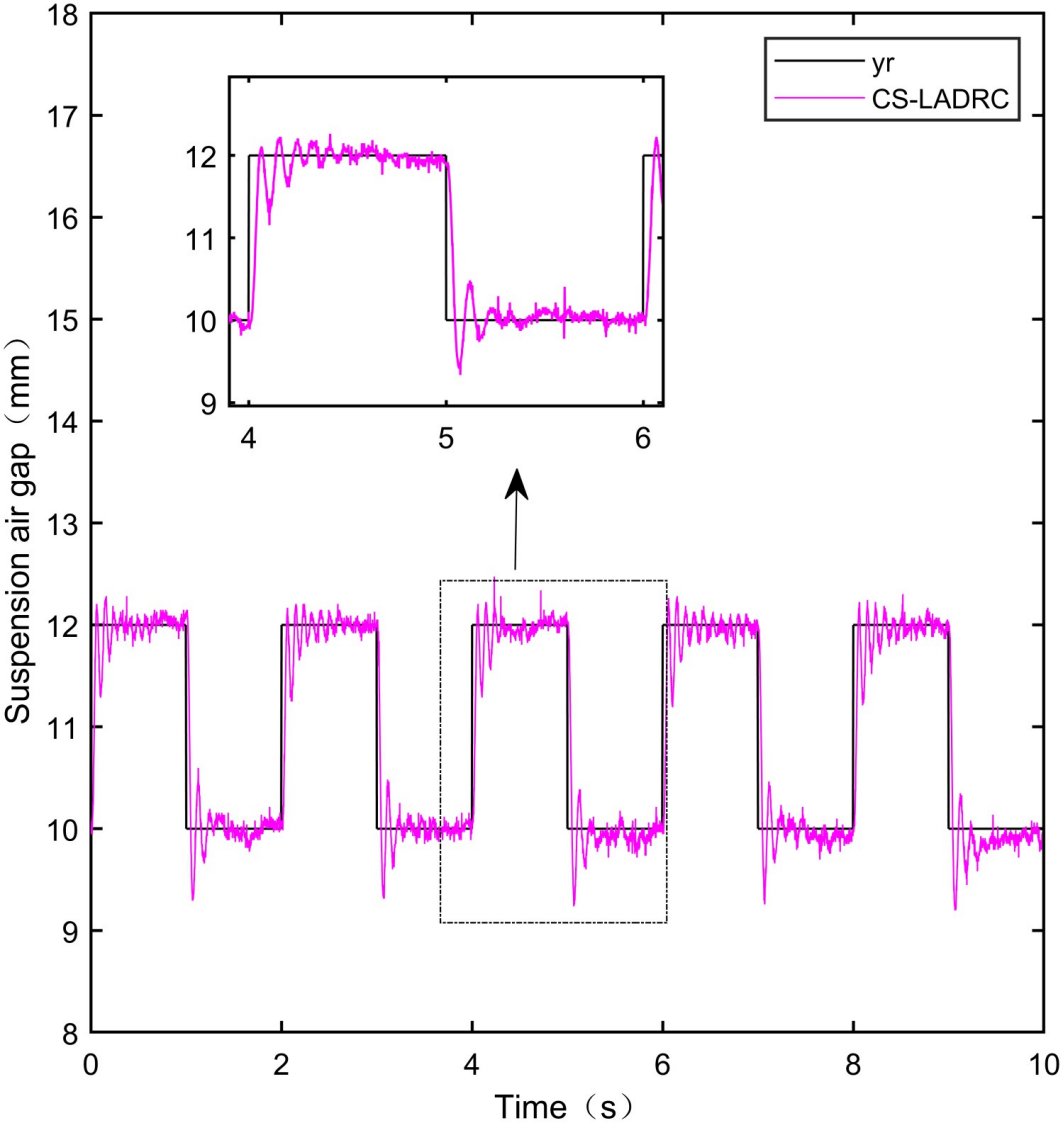
CS-LADRC square wave tracking disturbance experiment.

**Fig 49:**
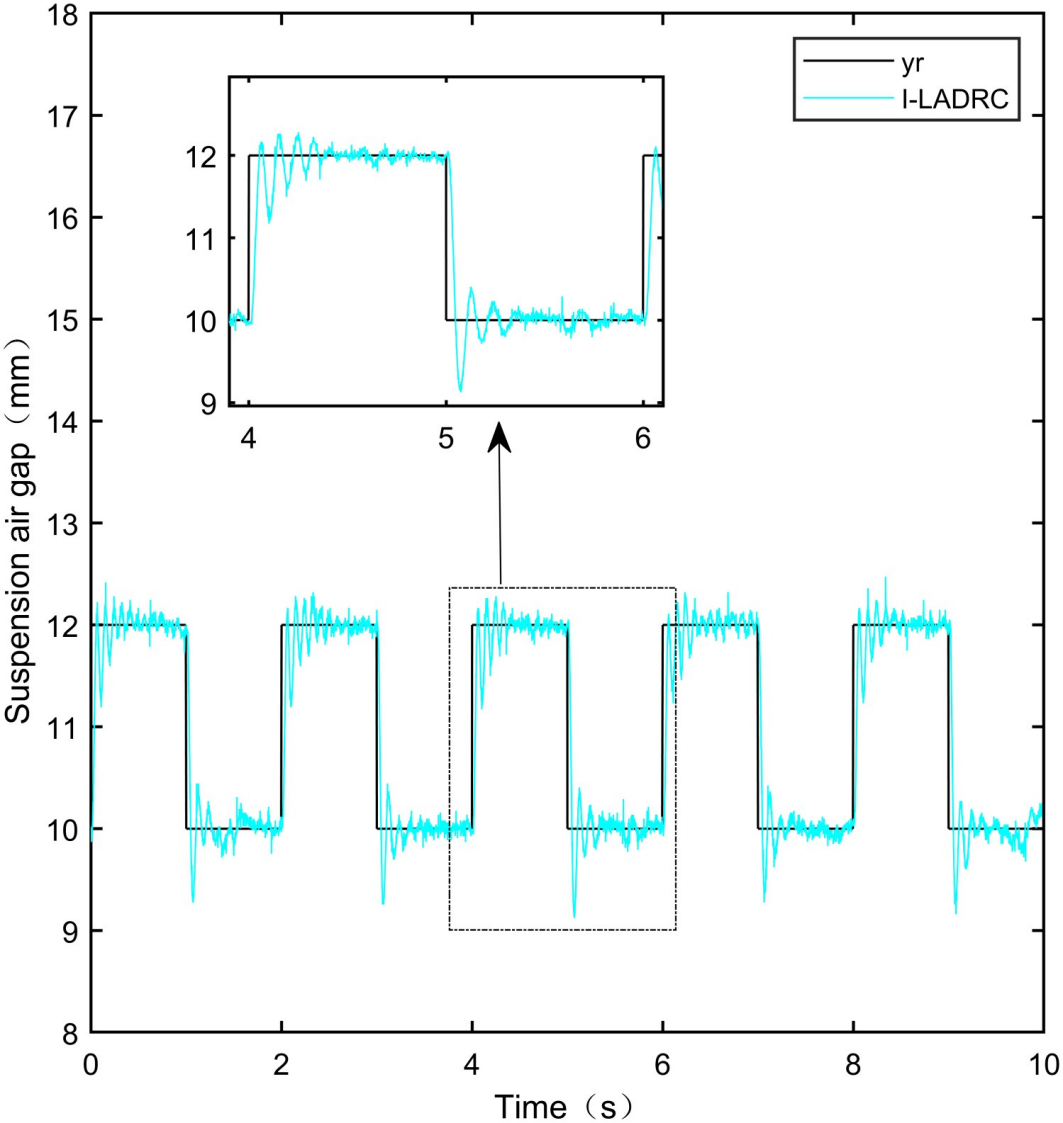
I-LADRC square wave tracking disturbance experiment.

**Fig 50:**
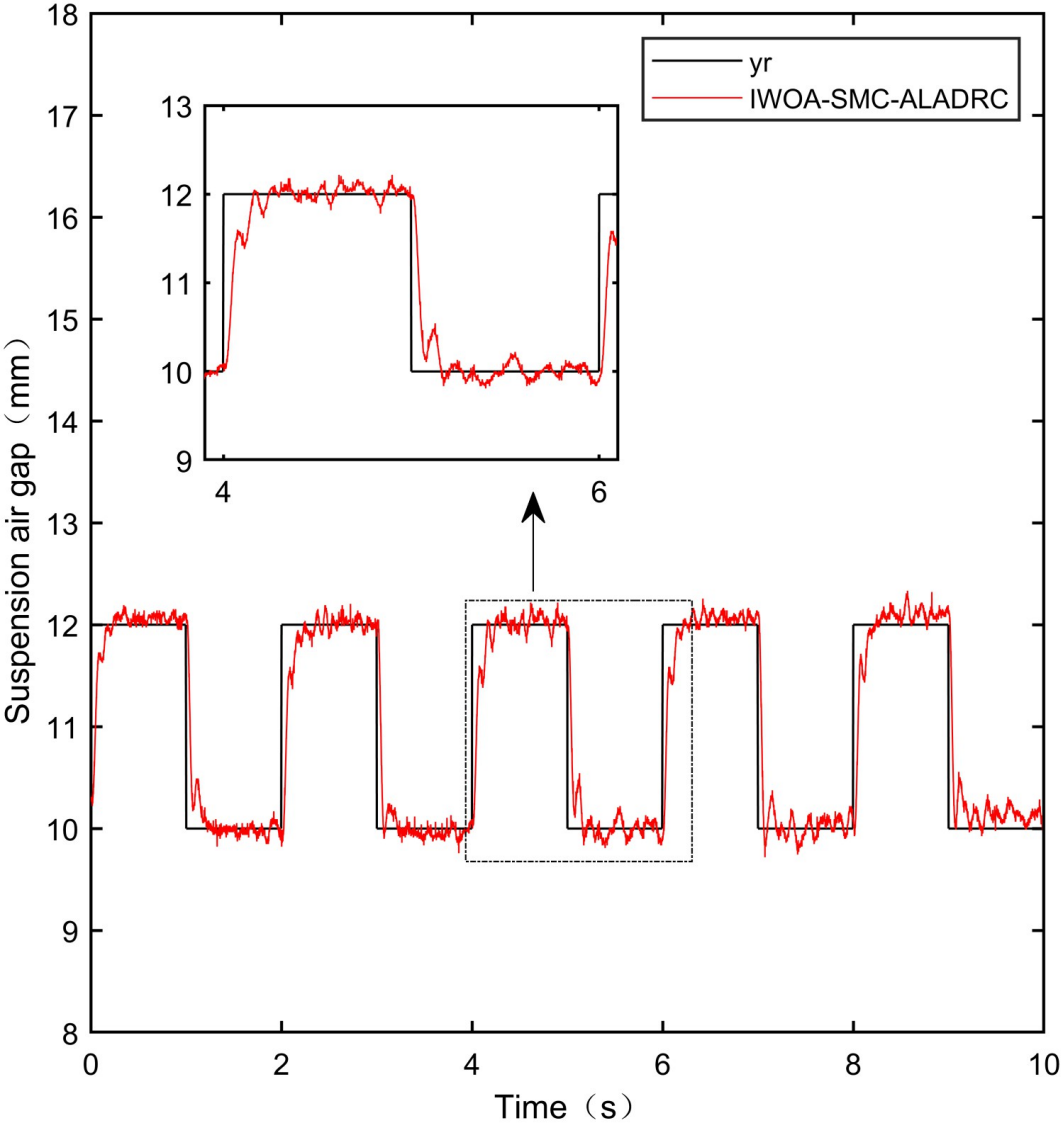
IWOA-SMC-ALADRC square wave tracking disturbance experiment.

This experiment is a square wave tracking disturbance experiment, where disturbance is applied to the system at the fifth second. Specifically, the goal is to analyze the anti-disturbance performance of the controller when the standard suspended air gap changes according to the square wave signal law. The disturbance effect is shown in Figs 46–50:

From Fig 46, it can be seen that the PID lost control and detached after the system was disturbed. From Figs 47–49, it can be seen that after disturbance, the deviation distances between the output suspension air gap of LADRC, CS-LADRC, and I-LADRC and the standard suspension air gap are 0.920mm, 0.792mm, and 0.945mm, respectively. However, it can be seen from Fig 50 that the deviation distance of IWOA-SMC-ALADRC is only 0.412mm. Compared to LADRC, CS-LADRC, and I-LADRC, the deviation distance of IWOA-SMC-ALADRC decreased by 55.22%, 47.98%, and 56.40%, respectively. It can be seen that when the standard suspended air gap changes with the square wave signal pattern, the anti-disturbance performance of IWOA-SMC-ALADRC is stronger than the other four control methods.

The experimental results are shown in Table 8.

**Table 8:** Error of the three controllers in the experimental verification.

	Controller				
Error	PID	LADRC	CS-LADRC	I-LADRC	IWOA-SMC-ALADRC
**Step signal error(mm)**	±0.678	±0.311	±0.301	±0.342	±0.135
**Disturbance error(mm)**	1.501	0.510	0.501	0.342	0.196
**Sine follow error(mm)**	±0.744	±0.421	±0.417	±0.394	±0.245
**Sine dieturbance error(mm)**	0.812	0.743	0.432	0.638	0.322
**Square follow error(mm)**	±1.762	±0.792	±0.801	±0.799	±0.312
**Square disturbance error(mm)**	-	0.920	0.792	0.945	0.412

## 8 Conclusion

This study addresses the issues of weak tracking and anti-disturbance performance in the application of traditional control algorithms in electromagnetic levitation systems. Taking the electromagnetic levitation ball system as the research object, a simulation model was established using the Taylor formula to linearize the electromagnetic levitation ball system at the equilibrium point. Combining robust sliding mode with adaptive linear active disturbance rejection control, and introducing an improved whale optimization algorithm to confirm the adjustable parameter values in the controller, stable operation of the electromagnetic levitation ball is achieved. The final results show that both simulation analysis, experimental verification, and data analysis demonstrate that IWOA–SMC–ALADRC has better anti-disturbance and tracking performance than PID, LADRC, CS-LADRC, and I-LADRC.

1. In terms of simulation analysis, when the standard suspension air gap is 10mm, the IAE, ITAE, and ITSE error values of IWOA-SMC-ALADRC are much smaller than the other four control methods. At the same time, compared with PID, LADRC, CS-LADRC, and I-LADRC, the adjustment time of IWOA-SMC-ALADRC has been reduced by 93.20%, 76.66%, 46.17%, and 24.87%, and the overshoot was reduced by 74.19%, 90.85%, 84.78%, and 76.69%. When the system is subjected to 5N disturbance, compared to PID, LADRC, CS-LADRC, and I-LADRC, the deviation distance of IWOA-SMC-ALADRC decreases by 74.58%, 92.48%, 89.45%, and 86.14%, respectively. When the interference is 10N, the deviation distance of IWOA-SMC-ALADRC decreases by 81.28%, 93.98%, 90.85%, and 89.08%, respectively. Therefore, when the input signal is a step signal, IWOA-SMC-ALADRC has better dynamic performance and anti-disturbance performance than the other four control methods.

2. In terms of simulation tracking performance analysis, regardless of whether the standard suspension air gap varies with sine or square wave laws, the suspension air gap output by IWOA-SMC-ALADRC can approach the standard suspension air gap to the maximum extent, and the IAE, ITAE, and ITSE errors are much smaller than the other four control methods. When the standard suspension air gap varies with the sine law, the deviation distance of IWOA-SMC-ALADRC decreases by 2.60%, 67.94%, 61.18%, and 56.08% compared to PID, LADRC, CS-LADRC, and I-LADRC, respectively. When the standard suspended air gap changes with the square wave pattern, the offset distances decrease by 52.64%, 61.28%, 48.69%, and 48.52%, respectively. Therefore, compared to the other four control methods, the suspended air gap output by IWOA-SMC-ALADRC can not only approach the standard suspended air gap to the maximum extent, but also have better anti-interference ability.

3. In terms of experimental verification, when the standard suspension air gap is 10mm, the fluctuation ranges of PID, LADRC, CS-LADRC, I-LADRC, IWOA-SMC-ALADRC are reduced by 80.09%, 56.59%, 55.15%, and 60.53% respectively. After the system is disturbed, the offset distance of IWOA-SMC-ALADRC is reduced by 86.94%, 62.67%, 60.88%, and 54.10%, respectively. When the standard suspended air gap varies with the sinusoidal signal, the fluctuation range of IWOA-SMC-ALADRC is reduced by 67.07%, 41.81% 41.25% and 37.82% compared to PID, LADRC, CS-LADRC, and I-LADRC, respectively. After adding interference, the offset distance of IWOA-SMC-ALADRC is reduced by 60.34%, 56.66%, 25.46%, and 49.53%. When the standard suspended air gap changes according to the square wave signal, the fluctuation range of IWOA-SMC-ALADRC is reduced by 82.29%, 60.60%, 61.08%, and 60.95% compared to PID, LADRC, CS-LADRC, and I-LADRC, respectively. After adding interference, PID loses control and falls off, and the offset distance of IWOA-SMC-ALADRC is reduced by 55.22%, 47.98%, and 56.40% compared to LADRC, CS-LADRC, and I-LADRC.

## Supporting information

S1 Table The data used for fitting the magnetic levitation ball experiment. (XLSX)
